# Increasing Aridity May Threaten the Maintenance of a Plant Defence Polymorphism

**DOI:** 10.1111/ele.70039

**Published:** 2024-12-31

**Authors:** Lauren N. Carley, Tom Mitchell‐Olds, William F. Morris

**Affiliations:** ^1^ University Program in Ecology Duke University Durham North Carolina USA; ^2^ Department of Biology Duke University Durham North Carolina USA; ^3^ Rocky Mountain Biological Laboratory Gothic Colorado USA; ^4^ Ecology & Evolution Department University of Chicago Chicago Illinois USA

**Keywords:** balancing selection, drought, glucosinolates, herbivory, integral projection model, polymorphism, total fitness

## Abstract

It is unclear how environmental change influences standing genetic variation in wild populations. Here, we characterised environmental conditions that protect versus erode polymorphic chemical defences in *Boechera stricta* (Brassicaceae), a short‐lived perennial wildflower. By manipulating drought and herbivory in a 4‐year field experiment, we measured the effects of driver variation on vital rates of genotypes varying in defence chemistry and then assessed interacting driver effects on total fitness (estimated as each genotype's lineage growth rate, *λ*) using demographic models. Drought and herbivory interacted to shape vital rates, but contrasting defence genotypes had equivalent total fitness in many environments. Defence polymorphism thus may persist under a range of conditions; however, ambient field conditions fall close to the boundary of putatively polymorphic environment space, and increasing aridity may drive populations to monomorphism. Consequently, elevated intensity and/or frequency of drought under climate change may erode genetic variation for defence chemistry in *B. stricta* .

## Graphical abstract

It is unclear how environmental change influences standing genetic variation in wild populations. Here, we characterised environmental conditions that protect versus erode polymorphic chemical defences in a short‐lived perennial wildflower by manipulating drought and herbivory in a 4‐year field experiment. Contrasting defence genotypes had equivalent total fitness in many environments, indicating that defence polymorphism may persist under a range of conditions; however, increasing aridity may drive populations to monomorphism.

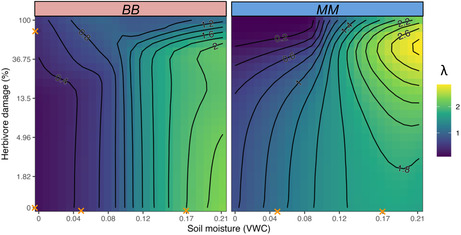

## 1 Introduction

Genetic variation determines whether populations can adapt to changing environments (Lande and Shannon 1996; Barrett and Schluter 2008) and has consequences for higher‐order ecological processes including trophic interactions (Wan et al. 2022), population persistence (e.g., Hanski and Saccheri 2006; Agashe 2009; Bozzuto et al. 2019; Carley et al. 2022), community composition and stability (Hughes and Stachowicz 2004; Barbour et al. 2015) and ecosystem function (Reusch and Hughes 2005; Hajjar, Jarvis, and Gemmill‐Herren 2008; Raffard et al. 2019). Understanding how genetic variation is maintained in wild populations is thus a central goal in evolutionary biology. Despite this, the ecological conditions that promote polymorphism are seldom explicitly characterised, and it is unclear how changing environments will affect standing genetic variation.

Plant defences are often particularly variable (Agrawal, Gorski, and Tallamy 1999; Iason et al. 2011; Züst et al. 2012) despite having consequences for fitness. This apparent paradox has motivated a large body of literature investigating why less effective defence strategies might persist. Evidence has thus emerged that plant defences can be costly and are often subject to fitness trade‐offs—for example, between resistance and tolerance (Stinchcombe and Rausher 2002), constitutive and induced defence (Bingham and Agrawal 2010), growth and defence (Züst, Rasmann, and Agrawal 2015), reproduction and defence (Strauss et al. 2002) and resistance to generalists versus specialists (Agrawal, Gorski, and Tallamy 1999). Trade‐offs help to explain how variation in herbivore pressure and resource availability may maintain multiple optimal defences across space and/or time. However, recent work demonstrates that other factors such as temperature (Hahn et al. 2019; Bont et al. 2020; Rotter, Christie, and Holeski 2022) and drought (Carley et al. 2021) influence selection on plant defences and that defence traits such as trichomes (Galdon‐Armero et al. 2018) and secondary metabolites (Hossain et al. 2013; Abuelsoud, Hirschmann, and Papenbrock 2016; Salehin et al. 2019) can mediate responses to abiotic stress. Thus, understanding the evolution of plant defence polymorphism may require explicit incorporation of abiotic contexts into more typical resource–economic approaches (Lin et al. 2023).

Whether defence traits experience selection by biotic interactions, abiotic conditions or both, quantitative relationships between selective drivers and fitness must be characterised to predict fates of defence polymorphisms in changing environments. This is rarely achieved empirically. For example, many studies of adaptation and evolution in wild populations test for local adaptation without identifying its drivers (Anderson et al. 2014). While some recent examples oppose this trend (Benning and Moeller 2019; Anderson and Wadgymar 2020), identification of selective drivers is usually achieved by manipulating putative drivers in binary treatments (e.g., ambient herbivory vs. herbivore exclusion; cf. Morris et al. 2020). This makes it challenging to predict evolutionary responses unless the effects of categorical driver manipulations (a) are quantified and (b) exactly match future conditions. Furthermore, the shape of organismal responses to environmental variation can be difficult to predict without sampling environmental space widely (Monroe, Cai, and Des Marais 2021). This challenge increases when selection is shaped by multiple drivers, which may covary and interact (Louthan et al. 2018; Hamann et al. 2020).

Although these obstacles limit precision when predicting evolutionary and genetic responses to environmental change, ecologists have made progress in quantifying population and community responses to identified drivers. For example, recent work has elucidated how continuous variation in geographic and environmental drivers alters population dynamics (Doak and Morris 2010, Sheth and Angert 2018, Campbell 2019, Morris et al. 2020), how interacting drivers influence population growth (Oldfather et al. 2021) and how life history mediates nonlinear responses to interacting drivers (O'Connell et al. 2024). Some of this progress has been stimulated by the development of integral projection models (IPMs; Ellner and Rees 2006; Merow et al. 2014), which allow straightforward modelling of vital rates in response to continuous driver variation. Demographic models such as IPMs integrate vital rates into estimates of total fitness or lineage growth rates (*λ*; Caswell 2001). This can be particularly useful in understanding net selection under fitness trade‐offs across vital rates and/or on perennial organisms for which it is difficult to quantify lifetime fitness directly (Schluter, Price, and Locke 1991). For example, some studies have shown the inferred magnitude or direction of selection to change depending on the fitness components considered (Ehrlén 2003; Gómez 2008; Ehrlén and Münzbergová 2009; Mojica and Kelly 2010). Estimates of total fitness such as *λ* integrate all vital rates (which are equivalent to the average fitness components across a lineage or population) and thus avoid such incomplete assessments of selection (Metcalf and Pavard 2007; Wadgymar et al. 2024).

Here, we combined population ecological and evolutionary genetic approaches to elucidate how biotic and abiotic variation shape selection on a focal polymorphism: defence chemical profiles in *Boechera stricta* (Brassicaceae). Previous work revealed that herbivory and drought both contribute to balancing selection on *B. stricta* defence chemistry; in addition to modulating herbivore defence, a gene‐controlling chemical defence influences survival under drought stress and plastic changes in morphology that influence water use (Carley et al. 2021). However, prior work focused on individual phenotypes and fitness components and did not manipulate drivers simultaneously; as such, the quantitative and potentially interactive effects of drought and herbivory on total fitness remain uknown. By extension, it is unclear which environmental conditions might protect or erode defence polymorphism. We transplanted genotypes conferring contrasting chemical profiles into a common garden where we manipulated drought and herbivory for 4 years, the full life span of the majority (~78%) of transplants. We then built genotype‐specific IPMs which we used to ask:

(Q1) How does natural selection driven by drought and herbivory shape total fitness of *B. stricta* genotypes differing in defensive chemistry?
(Q2) Which environmental conditions may maintain defence chemistry polymorphisms in *B. stricta* populations?
(Q3) What are the implications of biotic and abiotic environmental change for the maintenance of defence polymorphism?

## 2 Methods

### 2.1 Study System and Focal Trait

*Boechera stricta* (Brassicaceae) is a highly self‐pollinating perennial wild relative of Arabidopsis common across the western United States, and is a model system for evolutionary ecology and genetics (Rushworth et al. 2011, 2022). Like other Brassicaceae, *B. stricta* produces glucosinolates (GS), secondary metabolites that mediate interactions with herbivores and pathogens (Hopkins, van Dam, and van Loon 2009). One axis of GS variation, the proportion of GS derived from branched‐chain amino acids versus methionine (‘BC‐ratio’; Schranz et al. 2009), is controlled by the gene *BCMA1/3* (Prasad et al. 2012). Polymorphism in BC ratio and at the *BCMA1/3* locus have been maintained by balancing selection driven by both herbivory and drought (Carley et al. 2021). Near‐isogenic lines (NILs) homozygous at *BCMA1/3* for alleles conferring either branched‐chain or methionine‐derived GS (‘*BB*’ and ‘*MM*’ genotypes, respectively) have been generated via crossing and isolate the effects of these alleles in an otherwise homogenous genetic background (Prasad et al. 2012).

### 2.2 Manipulative Field Experiment

#### 2.2.1 Common Garden

We tested the fitness consequences of contrasting GS profiles by transplanting NILs in a common garden in Schofield, CO, USA (39.036°, −107.059°, 3145 m elevation). The garden was fenced aboveground to prevent trampling and ~0.3 m belowground to minimise intrusion by gophers.

We germinated and reared plants for 2 months under greenhouse conditions (Appendix S1) at Duke University (Durham, NC, USA) before shipping plants to Colorado. We transplanted 2300 juvenile plants into local vegetation in randomised, complete blocks containing 50 plants (25 *MM*, 25 *BB*) spaced at constant density over two cohorts: 2015 (*N* = 1500) and 2016 (*N* = 800; Table S1).

*B. stricta* seeds germinate in the springtime following snowmelt; surviving germinants live one season as vegetative rosettes, and after vernalisation may reproduce in the second year or later (Anderson and Wadgymar 2020). We transplanted rosettes autumn of each cohort, allowing for natural vernalisation shortly thereafter. We then monitored vital rates annually until 2019.

#### 2.2.2 Environmental Drivers

In this subalpine system, water availability during the growing season is driven primarily by snowmelt in the spring and monsoon rains in mid‐summer. This experiment spanned a range of ambient conditions, including substantial variation in both winter and summer precipitation (Table S2). Beyond this interannual variation in ambient conditions, we manipulated soil moisture and herbivory from 2017 to 2019. Manipulations spanned all field growing seasons for the 2016 cohort. The 2015 cohort experienced 1 year under ambient field conditions before manipulations began. The remaining sample size at the start of driver manipulations was 1605, ranging from 176 to 227 replicates per genotype per driver manipulation treatment (Table S3).

We manipulated soil moisture using rainout shelters to intercept precipitation (Yahdjian and Sala 2002; Shriver 2015) over ~50% of the surface area of each garden block. We included below‐ground barriers to reduce lateral movement of soil moisture (Appendix S1). In 2018, a year of low snowpack, early snowmelt and low summer precipitation (Table S2), we also supplemented soil moisture in control blocks by adding 5.7 L of water weekly in June. We monitored efficacy of this treatment by using a 12‐cm handheld soil moisture probe (Hydrosense, Campell Scientific, Logan, UT) to measure volumetric water content (VWC) in each block repeatedly in 2017–2018. We regressed these block‐level measurements upon soil moisture data collected on the same dates in a separate experiment (Anderson and Wadgymar 2020) to fit models from which to estimate daily block‐level soil moisture in our common garden from 2015 to 2019 (Appendix S1; Figures S1–S3). We monitored block‐level soil temperature and light availability repeatedly in 2017–2018 to test for effects of shelters on environmental conditions other than soil moisture (Appendix S1).

We manipulated herbivory at the individual plant level. Weekly throughout the growing seasons in 2017–2019, we sprayed a bacterial endotoxin insecticide (Thuricide BT Caterpillar Control, Southern Ag, Rubonia, FL) on each plant assigned to the reduced herbivory treatment (Appendix S1) according to the manufacturer's instructions, targeting plants with a plastic cone to minimise drift. We sprayed an equal volume of water onto control plants. BT has a general mechanism of action and is advertised to deter a variety of insects including larval butterflies, moths, beetles and flies. We estimated herbivore damage to aboveground tissue to determine the efficacy of herbivory manipulations (Appendix S1).

We tested the effects of rainout shelters on soil moisture, soil temperature and light availability (all measured at the block level) using linear models with census year, rainfall treatment and their interaction as fixed effects. We tested effects of insecticide treatment on herbivore damage (measured at the individual plant level) by first modelling the probability of receiving herbivory in response to fixed effects of insecticide treatment, drought treatment, census year and their interactions using a generalised linear model with a binomial distribution and logit link function. Second, among plants that received herbivory, we modelled per cent leaf area removed in response to insecticide treatment, drought treatment, census year and their interactions using a linear model. To improve model residuals, we log‐transformed the amount of herbivore damage (adding a small constant to avoid dropping zeros). For analyses of treatment efficacy, we used data spanning the driver manipulations (2017–2019). We tested significance of linear model effects using ANOVA, and of logistic regression effects using likelihood ratio tests (‘lmtest’ package in R; Zeileis and Hothorn 2002) comparing the full model to nested reduced models, dropping one effect at a time.

Treatments successfully manipulated the drivers of interest (Figures S4 and S5). Rainout shelters reduced soil moisture by 3.1%, a relative reduction of 27% compared to control blocks (Figure S5A) without affecting soil temperature or light availability (Figure S6). Insecticide treatment reduced the probability of herbivory by 6.8%, a relative reduction of 9.6% (Figure S5B); and the amount of herbivory by 0.33%, a relative reduction of 12.6% (Figure S5C).

#### 2.2.3 Plant Measurements

We censused plants at the end of each growing season from 2016 to 2019, scoring individuals for survival, size (height), reproduction and herbivore damage (Appendix S1). We measured total fruit length directly, and estimated seed set using a linear regression of seed number on fruit length using data from a separate nearby experiment (Appendix S1; Anderson and Wadgymar 2020).

### 2.3 Demographic Analyses

To answer our research questions, we used data from our field experiment to parametrise genotype‐specific and driver‐dependent demographic models.

#### 2.3.1 Vital Rate Regressions and Model Selection

For each NIL genotype, we estimated 10 vital rates describing the life cycle of *B. stricta* : germination, survival of ungerminated seeds in the seed bank, germinant survival in the first year of life, size of surviving first‐year recruits, adult survival, mean adult growth, variance in growth, probability of bolting, probability of reproducing and seed number. We modelled seven vital rates using census data from our manipulative common garden experiment and three (seed germination, ungerminated seed survival and germinant survival) using previously published data (Anderson and Wadgymar 2020). Vital rate models for plants older than seedlings included plant size and both drivers as potential predictors. We outline our general modelling approach here, with details provided in Appendix S2.

For size‐ and driver‐dependent vital rates, we began by fitting a global model with each vital rate as the dependent variable and fixed main effects of size, soil moisture and herbivory and all possible two‐ and three‐way interactions as independent variables. Although driver manipulations were implemented categorically, we used continuous values of herbivory and soil moisture (leveraging variation both within and across treatments; cf. Shriver 2015; Figure S7) and plant height as independent variables. We modelled probabilistic transitions using generalised linear models with a binomial distribution and logit link function, and continuous transitions using linear models.

We modelled vital rates allowing the effects of interacting drivers to differ in magnitude and direction across genotypes. Specifically, we fit separate vital rate regressions for each NIL genotype and then used AICc (‘MuMIn’ package; Barton 2019) to select among models containing all possible subsets of the global model (Appendix S3). If two or more models had similar AICc scores (ΔAICc < 2), we favoured the model with the lowest absolute AICc, which was generally the most parsimonious. Size was retained as a predictor of most vital rates, but which selective drivers, driver interactions and Driver × Size interactions were retained differed (Table S5).

Adult growth in *B. stricta* depends strongly on transitions between discrete reproductive states structured on bolting, a prerequisite to flowering and reproduction (Appendix S2; Figure S8). To accommodate this biologically relevant variation, we modelled growth and reproduction as dependent on a bolting/nonbolting state variable (Appendix S2).

#### 2.3.2 Integral Projection Modelling

To assess net effects of drivers on fitness, we built IPMs to calculate *λ* of each NIL. We constructed IPMs using standard methods (Ellner and Rees 2006; Merow et al. 2014), with modifications to accommodate state‐specific vital rates and to incorporate the seed bank into the discretised IPM kernel (Carley et al. 2022). Full model details are provided in Appendix S2.

We evaluated each IPM across a biologically relevant range of herbivory and soil moisture values: 0%–100% aboveground tissue removal by herbivores (just beyond the maximum observed value of herbivory of 90%), and 0 through 1.25× the maximum observed soil moisture value. We discretised each driver into 25 bins (equal bin width for soil moisture; log‐scale bin width for herbivory to better reflect the observed distribution of herbivory values; Figure S7), and estimated *λ* in each of these 25 × 25 = 625 environmental combinations. Outcomes when modelling *λ* across finer environmental resolutions were qualitatively identical. While the range of modelled conditions deliberately extends slightly beyond observed driver ranges, transplants in the experimental garden experienced most modelled combinations of drought and herbivory (Figure S9).

#### 2.3.3 Parameter Uncertainty

We accounted for parameter uncertainty by taking 1000 parametric bootstrap samples from the multivariate normal distributions of the vital rate parameters (Appendix S2). We calculated *λ* for each genotype across environment space using the sampled vital rate coefficients and used the distributions of *λ* to answer our research questions.

#### 2.3.4 Assessing Outcomes

##### 2.3.4.1 Q1: Net Effects of Drought and Herbivory on Total Fitness

We assessed the effects of drought and herbivory on fitness components by comparing the vital rate regressions of the two NILs. We approximated the contributions of each driver to *λ* as mediated by each vital rate by measuring the proportional change in *λ* when a driver's effects on each vital rate were removed one at a time (Appendix S2).

We assessed net effects of both drivers on genotype‐specific total fitness by evaluating the topography of *λ* across bivariate driver space. We assessed natural selection across environments by comparing *λ* of the two genotypes in each modelled environment, considering differences in *λ* to be significant if the 95% CI of differences across 1000 bootstrap samples did not overlap 0 (Appendix S2).

##### 2.3.4.2 Q2: Maintenance of Polymorphism

We assessed environmental conditions in which each genotype may persist by identifying environments where *λ* equalled or exceeded replacement (i.e., the 95% CI of the bootstrapped distribution overlapped or exceeded 1). We identified ‘putatively polymorphic environments’ as combinations of drought and herbivory levels in which both genotypes can persist at or above replacement (Criterion I) and the difference in lifetime fitness between NIL genotypes is not significantly different from 0 (Criterion II).

##### 2.3.4.3 Q3: Genetic Variation Under Environmental Change

To assess whether current combinations of soil moisture and herbivory may maintain GS polymorphism, we asked whether driver combinations observed in our common garden fell within putatively polymorphic environments (see Q2). We assessed effects of future changes in herbivory and drought on polymorphism by asking whether directional shifts in either or both drivers away from contemporary levels may move *B. stricta* populations out of putatively polymorphic environment space.

## 3 Results

### 3.1 Q1: Net Effects of Drought and Herbivory on Total Fitness

**FIGURE 1:**
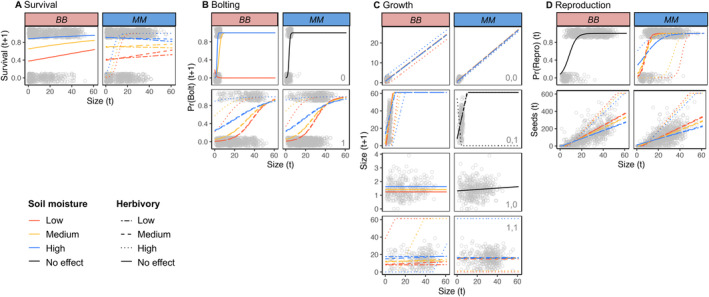
Drought and herbivory interact to shape size‐dependent *B. stricta* vital rates. Panels show variation in how vital rates respond to size (size *t*) across different soil moisture and herbivory levels for each glucosinolate genotype (*BB*: branched‐chain glucosinolate homozygote; *MM*: methionine glucosinolate homozygote). (A) Survival. (B) Bolting; state‐specific probabilities are shown across rows, with the bolting state in time *t* in grey text. (C) Growth. Across rows, state‐specific growth patterns are shown, with the bolting state in times *t, t +* 1 given in grey text. (D) Reproduction (probability of reproduction, upper; seed number produced conditional upon reproduction; lower). When retained following model selection (Methods), effects of herbivory and soil moisture (VWC: volumetric water content) on each vital rate are shown with separate lines fit onto data at different driver percentiles; colours represent soil moisture levels (0th percentile, red; 50th percentile, orange; 100th percentile, blue); and line types indicate herbivory levels (0th percentile, long dash; 50th percentile, dashed; 100th percentile, dotted). Black lines represent vital rates in which there was no effect of soil moisture following model selection, and solid lines represent vital rates in which there was no effect of herbivory following model selection. *Note:* Regression lines here show the predicted effect of size, herbivory and drought on vital rates as estimated by the mean parameter values in each vital rate regression following model selection. In full demographic models, uncertainty was accounted for by sampling parameter values from the variance–covariance matrix of parameters for each vital rate model (Methods; Appendix S2).

Our longitudinal, manipulative field study revealed variable effects of herbivory and drought on the vital rates of *B. stricta* GS genotypes (Figure 1; Tables S5 and S6; Appendix S3). Overall, *BB* was more sensitive to drought (with eight vital rates negatively affected by reduced soil moisture either at small or all sizes) and *MM* was more sensitive to herbivory (with seven vital rates responding negatively to herbivore damage, vs. two and four vital rates showing positive and neutral effects, respectively). The effects of herbivory on *BB* and drought on *MM* were mixed, with positive, negative and neutral effects on different vital rates (Table S6). Interactions among herbivory, drought and size also played important roles in shaping vital rates. After model selection, 22 interaction terms in 14 vital rate regressions were retained, and of 19 vital rate regressions that retained effects of drought and/or herbivory, 14 also retained interactions; 5 of these interactions were between drought and herbivory, while the other 9 were between plant size and one or both drivers. Thus, contrasting GS alleles in *B. stricta* are not maintained by a simple trade‐off of drought tolerance and herbivore defence; both drought and herbivory influence fitness in complex and interacting ways across the life cycle.

**FIGURE 2:**
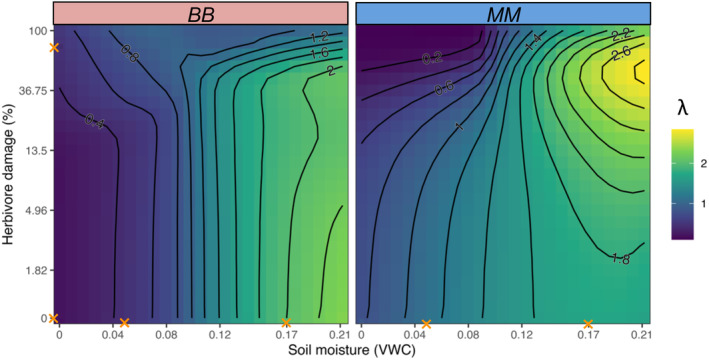
Drought and herbivory interact to shape total fitness. Total fitness (*λ*) was estimated by integrating longitudinal data on fitness components across the life cycle throughout the field experiment, separately for each focal genotype (*BB:* branched‐chain glucosinolate homozygote; *MM:* methionine glucosinolate homozygote). Total fitness of 1 indicates that a lineage's abundance is stable through time. Here, the median *λ* value from 1000 bootstrap samples of vital rate regression coefficients is shown; upper and lower confidence intervals of fitness surfaces, accounting for uncertainty in vital rate parameters, are shown in Figure S10. Orange x marks on the *x*‐ and *y*‐axes denote the minimum (left or lower x) and maximum (right or upper x) values of soil moisture and herbivory, respectively, observed during the field experiment.

Total fitness of both genotypes was shaped by both drivers (Figure 2). Both genotypes experienced strong net selection by drought, with fitness declining with decreasing soil moisture. However, the magnitude of drought‐mediated selection was greater for *BB* than for *MM*, as indicated by steeper changes in *λ* across the soil moisture axis. Accordingly, the proportional contributions of soil moisture to *λ* via vital rates were greater on average than the contributions of herbivory to *λ* (Table 1). The effects of drivers on *λ* were mediated predominantly by their effects on survival and bolting for both genotypes (Table 1).

**TABLE 1:** Approximate proportional contributions of each driver to *λ* as mediated by individual vital rates (*c;* Appendix S2).

Vital rate	Contribution of herbivory to *λ* via vital rate		Contribution of soil moisture to *λ* via vital rate	
	*BB*	*MM*	*BB*	*MM*
*s*	0	−0.00528	**0.66391**	**0.62828**
*b*_*0*_	0	0	0.05936	0
*b*_*1*_	0.00443	**0.00940**	0.07001	0.13741
*g*_*00*_	−0.00016	−0.00024	−0.00364	−0.00393
*g*_*01*_	−0.00364	0.00096	0.04092	0
*g*_*10*_	0	0	0.01082	0
*g*_*11*_	−**0.00489**	−0.00323	0.03364	0.01611
*v*_*00*_	0	−0.00010	0	−0.00306
*v*_*01*_	0	0	−0.01474	0
*v*_*10*_	0	0	−0.00190	0
*v*_*11*_	0	0.00181	0	−0.00286
*r*	0	−0.00119	0	−0.00319
*f*	0.000397	0.00062	−0.00420	−0.00504

*Note:* Vital rate regression names and functional forms are defined in Table S5; in general, they are as follows: Survival (*s*), bolting (*b*), growth (*g*), variance in growth (*v*), probability of reproduction (*r*) and fecundity (*f*; i.e., seed set). Among state‐dependent vital rates, single subscripts indicate the past bolting state and double subscripts indicate the past and present bolting state (1 = bolting; 0 = not bolting). The fill colour of each cell in the table corresponds to the strength of the proportional contribution of a driver to *λ* through that vital rate, with darker blue indicating stronger negative effects and darker red indicating stronger positive effects. Cells with grey backgrounds and text indicate vital rates in which the driver of interest was not retained in the best‐fit vital rate regression following model selection. The vital rate mediating the strongest contributions of each driver to *λ* for each genotype (largest absolute value of *c* per column) is marked with bold text.

In wetter environments, the fitness optima of the two genotypes occur at different herbivory levels; *BB* has highest fitness at low to moderate levels of herbivory, while *MM* has highest lifetime fitness at moderate to high levels of herbivory (indicating potential compensatory responses to herbivory in this genotype; Garcia and Eubanks 2019). Upper and lower confidence limits of the fitness surface showed the same basic patterns (Figure S10).

### 3.2 Q2: Maintenance of Polymorphism

**FIGURE 3:**
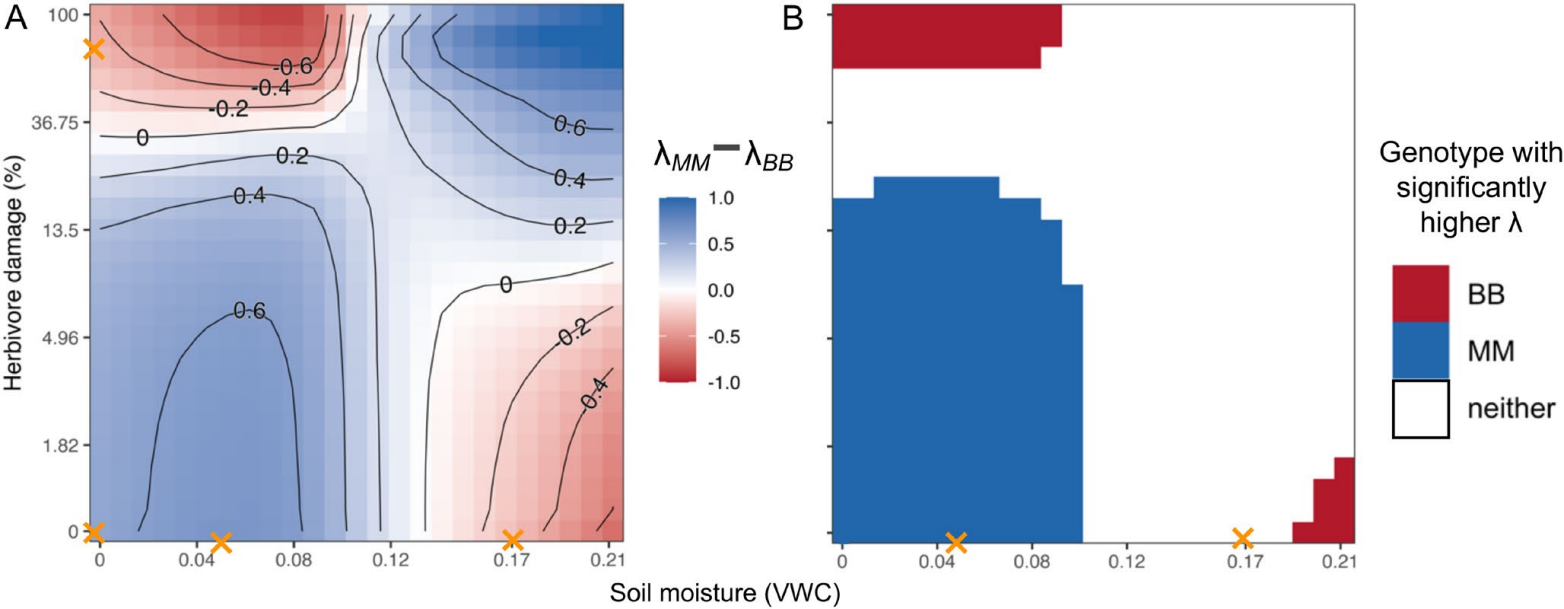
The defence alleles favoured by natural selection depend on biotic and abiotic conditions. (A) Median differences between *MM* and *BB* total fitness across 1000 bootstrap samples of vital rate parameter estimates show that *MM* has higher fitness in low soil moisture, low‐herbivory and high soil moisture, high‐herbivory environments; conversely, the *BB* genotype has higher lifetime fitness in high‐moisture, low‐herbivory and low‐moisture, high‐herbivory environments. In a ‘saddle’ of intermediate values, lifetime fitness is equivalent or nearly equivalent across genotypes. (B) Despite this, the bootstrapped distribution of the difference in lifetime fitness between genotypes overlapped 0 (i.e., was not significant; white shading) in many high soil moisture environments. In low soil moisture environments, bootstrapped lifetime fitness values were significantly higher for the *MM* genotype in low‐herbivory environments and for the *BB* genotype in high‐herbivory environments. Orange marks are as in Figure 2.

The *MM* genotype had relatively higher fitness when soil moisture and herbivory were both low or both high. Conversely, the *BB* genotype had relatively higher fitness when either herbivory or soil moisture, but not both, was high (Figure 3A). However, bootstrap simulations showed that at higher soil moisture levels, most genotypic differences in fitness were not significantly different from 0 (Figure 3B). Thus, despite contrasting genotypes showing differential and sometimes strong effects of drought and herbivory on vital rates, they have equivalent total fitness across medium to high soil moisture levels. In other words, net selection on GS variants may be weak unless soils are dry.

**FIGURE 4:**
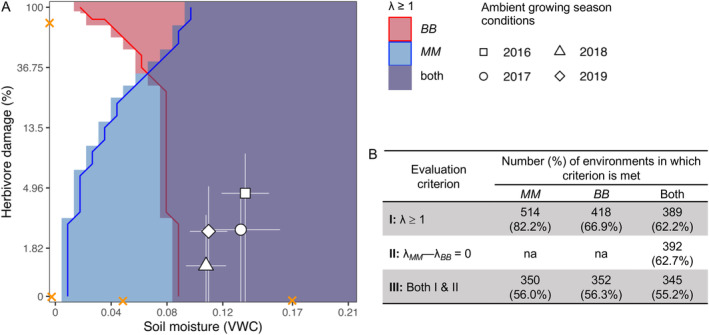
A wide range of environments supports demographic persistence of both defence alleles and thus may support polymorphism. (A) Any modelled environmental conditions in which lifetime fitness was equal to or greater than replacement (*λ* ≥ 1) are shaded (red shading: *λ* ≥ 1 for *BB*, the branched‐chain glucosinolate homozygote; blue shading: *λ* ≥ 1 for *MM*, the methionine glucosinolate homozygote). Environments in which both genotypes can persist above replacement are shaded purple. ‘Persist above replacement’ indicates that in 1000 bootstrapped simulations, the central 95% of the distribution of *λ* estimates overlapped with or exceeded 1. Overlaid white points and lines represent ambient environmental conditions in the field site across 4 years of study (mean ± 1 SD). Orange marks are as in Figure 2. (B) Across the range of modelled environment space, the number of cells in which each of three criteria is met is evaluated. Criterion I asks whether either or both glucosinolate genotypes can persist at or above demographic replacement (i.e., *λ* ≥ 1). Criterion II asks whether the difference in lifetime fitness between glucosinolate genotypes is statistically equivalent to 0 across bootstrap simulations. Criterion III asks whether both Criterion I and Criterion II are met, that is, whether glucosinolate polymorphism is expected to persist within populations.

Furthermore, many environmental combinations permit one or both genotypes to persist at or above replacement (*λ* ≥ 1; Figure 4). While *MM* can persist in a greater proportion of environmental space than *BB* (82.2% vs. 66.9% of 625 modelled environmental combinations, respectively), over half of tested environmental conditions (62.2%) can support persistence of both defence genotypes. Thus, a range of modelled environments satisfies both Criterion I (persistence of both genotypes is possible) and II (net fitness difference between genotypes is 0) for supporting polymorphism.

### 3.3 Q3: Genetic Variation Under Environmental Change

Unmanipulated levels of soil moisture and herbivory in the field fell within putatively polymorphic environmental space (Figure 4). However, natural conditions fell close to the boundary of polymorphism and monomorphism on the soil moisture axis. In environments drier than our study sites, *MM* alleles were selectively favoured under low‐to‐moderate herbivory, while *BB* alleles were favoured under high herbivory (Figure 3).

## 4 Discussion

### 4.1 Environmental Contexts of Balancing Selection on GS Polymorphism

Under anthropogenic change, whether the amount of standing genetic variation in populations is sufficient to permit adaptation to future environments has become an increasing concern (Etterson and Shaw 2001). Variation in traits underlying ecological interactions such as herbivore defence can also affect ecological communities (van Loon et al. 1992; Poelman, van Loon, and Dicke 2008; Sun et al. 2009; Johnson, Velland, and Stinchcombe 2009; Reznick 2013), so understanding selection of such polymorphisms is important from both ecological and evolutionary perspectives (Thompson 1998). To explore the ecological conditions that may protect defence polymorphism in *B. stricta* , we parameterised an IPM with two continuous environmental drivers: herbivory and drought. Past work showed effects of both drivers on individual fitness components (survival and reproduction) and traits that underlie them (herbivore defence and drought response). We expand upon prior studies to synthesise the long‐term effects of drivers on total fitness of two near‐isogenic genotypes differing in defensive chemistry.

Vital rate regressions show that GS genotypes respond differently to drought and herbivory, particularly for survival, bolting, juvenile growth and the probability of reproduction (Figure 1). In general, some patterns corroborate past findings; for example, the *MM* genotype, which adjusts morphology to use water more conservatively under drought (Carley et al. 2021), is insensitive to drought in many vital rates or, in some cases, is stimulated by drought while *BB* is not. However, vital rate regressions also revealed complex interactions among herbivory, drought and plant size that are difficult to intuit when assessing effects of these drivers individually. Ultimately, interacting and sometimes opposing effects of drivers on vital rates shape complex topographies when integrated into total fitness (Figure 2). Despite this, both GS genotypes maintain sufficiently high fitness to persist at or above replacement under most biologically relevant driver combinations that we evaluated (Figure 4). In many of these environments (62.7% of all modelled environments or 88.9% of environments where both genotypes persist above replacement), genotypic differences in total fitness, after accounting for parameter uncertainty, are zero (Figures 3 and 4). Thus, genetic differences expressed at the level of fitness components do not preclude the possibility of contrasting defence strategies persisting within populations, potentially at similar frequencies. In other words, integrated metrics like *λ* which incorporate organisms' variable responses to drivers across life history may be necessary for understanding net natural selection.

### 4.2 Environmental Change, Driver Covariance and Polymorphism

Although we identified a range of drought and herbivory combinations that may support *B. stricta* populations that are polymorphic for defence chemistry, natural conditions observed in the field during our study fell close to the boundary of putatively polymorphic environment space (Figure 4). Climate models predict decreased snowfall and earlier snowmelt in the western United States (Higgins and Shi 2001) and shifts in the timing of monsoon rains (Seth et al. 2011), which may lengthen and intensify early summer drought in our study region (Sloat et al. 2015). Increasing drought across years, and/or increasing variation in soil moisture across microhabitats within years, may threaten the maintenance of GS polymorphism in *B. stricta* populations.

Our field manipulations also revealed that drought exacerbates herbivory; across 3 years of driver manipulations, drought treatment increased the amount of herbivory on damaged plants (Figure S5C) and block‐level soil moisture and herbivory were negatively correlated (Figure S5E). Correlations among climate variables and herbivory are frequently documented (Hamann et al. 2020), especially across broad latitudinal (Salazar and Marquis 2012) and altitudinal (Rasmann et al. 2014) gradients. Our results, reflecting variation at the scale of meters within a single garden, demonstrate that abiotic and biotic drivers of selection can covary significantly at the microgeographic scale. Thus, changes in drought may elicit concomitant shifts in herbivory (e.g., via changes in abundance, composition or metabolism of arthropods; Chown and Gaston 1999) and/or herbivory's effects on plant fitness (e.g., via effects of drought on the expression of resistance; Tariq et al. 2013; Nguyen et al. 2016, Pezzola, Mancuso, and Karban 2017). This supports the general finding that environmental change may alter natural selection and population dynamics both directly through abiotic shifts and indirectly by modifying biotic interactions (Louthan et al. 2018).

In the case of *B. stricta* , increased drought should reduce GS polymorphism (Figure 4), but herbivory should determine which allele is favoured in drier environments (Figure 3B). Specifically, correlated changes in drought and herbivory may favour branched‐chain GS, since *B* alleles have higher total fitness in low‐moisture, high‐herbivory environments (Figure 3A). However, almost all environments in which *B* alleles were significantly favoured fell outside of the range of observed combinations of drivers used to parametrise our models (see axis markings on Figures 3 and 4 and Figure S9). Thus, while our models suggest that low‐moisture, high‐herbivory environments favour branched‐chain GS, empirical data sampling broader environment space is needed to corroborate this. Conversely, shifts in soil moisture only may favour methionine‐derived GS, since *M* alleles have higher lifetime fitness in lower‐moisture, low‐herbivory environments.

Without evolution of drought tolerance or avoidance, decreases in soil moisture may also compromise viability of *B. stricta* populations; *λ* of both focal genotypes declined with decreasing soil moisture (Figure 2), and the *BB* genotype had *λ* < 1 when VWC was below ~8% (Figure 4). This corroborates other studies describing *B. stricta* population growth rates consistently below replacement in lower‐elevation environments more arid than our focal site (Anderson and Wadgymar 2020) and negative effects of declining soil moisture on population growth rates of other alpine plant species (Oldfather et al. 2021).

Even if conditions do not become consistently dry enough to threaten *B. stricta* population viability, temporal fluctuations in soil moisture may erode genetic variation for defence chemistry. In dry years, when selection on GS is stronger, allele frequencies may shift towards monomorphism in declining populations. In wet years, populations may recover, but with allele frequencies perturbed due to selection in dry years. This may increase the likelihood of stochastic fixation of alleles favoured during drought even if selection is weak or relaxed entirely in wetter years. This possibility is consistent with the population genetic expectation that temporal fluctuations in selection should erode rather than promote polymorphism in finite populations (Hedrick 1976).

### 4.3 Ecological Approaches to Understanding Selection and Variation: Strengths and Limitations

Ultimately, to fully understand the fate of genetic polymorphism, population genetic parameters such as (non‐)random mating, migration and dispersal, rates of mutation and genetic drift and allelic dominance must be considered in addition to fitness effects of alleles in different environments (Hedrick 1986, 1999). We could not explicitly characterise these parameters, but we encourage future research on these topics. Nevertheless, demographic approaches offer advantages, including straightforward integration of fitness in perennial organisms with overlapping generations. *B. stricta* is also highly self‐pollinating (*F*_IS_: 0.74–0.97) and rarely heterozygous (Song et al. 2006). Thus, characterising *λ* for homozygous genotypes is useful both because of its ability to integrate sometimes contrasting effects of selection across life‐history stages and because it is a reasonable biological approximation of the persistence of inbred, self‐pollinating genetic lineages under different environmental conditions.

Leveraging regression‐based approaches also facilitates inference about the effects of continuous environmental drivers on fitness, rather than fitness differences across discrete environments (Merow et al. 2014). Empirical studies characterising effects of continuously varying ecological drivers on fitness are still uncommon, but are increasing because they are particularly important for understanding responses to selection under future climates.

Finally, while we argue that equating *λ* with total fitness is useful given both our research questions and the biology of our study organism, our demographic approach also has limitations. Particularly, we provide density‐independent estimates of *λ* without effects of conspecific or heterospecific competition or facilitation. With experimental genotypes planted in randomised, complete‐block designs at constant density, this is not unreasonable. However, density dependence and neighbour interactions likely contribute to more complex selection and population dynamics in natural populations. We also model selection on defence alleles as frequency independent, but this is consistent with past findings (Carley et al. 2021).

## 5 Conclusions and Implications

We show that GS profiles experience selection by both herbivory and drought, as evidenced by significant driver effects on vital rates and variation in the magnitude and direction of those effects across genotypes. We also show that GS genotypes should persist polymorphically under a range of environmental parameter space. However, increasing aridity may erode defence polymorphism and, in more extreme cases, threaten population viability. While there are some limitations to the evolutionary inferences that can be drawn from demographic models, we provide a transferrable framework for assessing effects of continuous environmental variation on the maintenance of genetic and phenotypic diversity within and across populations. In addition, while ecologically important traits in other species may be shaped by selective drivers different from those identified here, our results highlight generally that biotic and abiotic effects interact to shape selection in ways that may be difficult to intuit in single‐driver studies.

## Author Contributions

L.N.C. designed the experiment, collected the data and analysed the data. T.M.‐O. provided genetic resources and support for the field experiment. W.F.M. contributed to data analysis and experimental design. L.N.C. wrote the first draft of the manuscript, and all authors contributed to manuscript revisions.

## Conflicts of Interest

The authors declare no conflicts of interest.

### Peer Review

The peer review history for this article is available at https://www.webofscience.com/api/gateway/wos/peer‐review/10.1111/ele.70039.

## Supporting information


Data S1.

## Data Availability

Data and code supporting this manuscript are publicly available in the Dryad digital repository (DOI: 10.5061/dryad.18931zd4s).

## References

[ele70039-bib-0001] Abuelsoud, W. , F. Hirschmann , and J. Papenbrock . 2016. “Sulfur Metabolism and Drought Stress Tolerance in Plants.” Drought Stress in Plants, edited by M. A. Hossain , S. H. Wani , S. Bhattacharjee , D. J. Burritt , and L.‐S. P. Tran , vol. 1, 227–248. Switzerland: Springer International Publishing.

[ele70039-bib-0002] Agashe, D. 2009. “The Stabilizing Effect of Intraspecific Genetic Variation on Population Dynamics in Novel and Ancestral Habitats.” American Naturalist 174: 255–267. 10.1086/600085 19519220

[ele70039-bib-0003] Agrawal, A. A. , P. M. Gorski , and D. W. Tallamy . 1999. “Polymorphism Im Plant Defense Against Herbivory: Constitutive and Induced Resistance in *Cucumis sativus* .” Journal of Chemical Ecology 25: 2285–2304.

[ele70039-bib-0004] Anderson, J. T. , and S. M. Wadgymar . 2020. “Climate Change Disrupts Local Adaptation and Favours Upslope Migration.” Ecology Letters 23: 181–192. 31729141 10.1111/ele.13427

[ele70039-bib-0005] Anderson, J. T. , M. R. Wagner , C. A. Rushworth , K. V. S. K. Prasad , and T. Mitchell‐Olds . 2014. “The Evolution of Quantitative Traits in Complex Environments.” Heredity 112: 4–12. 23612691 10.1038/hdy.2013.33 PMC3860162

[ele70039-bib-0006] Barbour, M. A. , M. A. Rodriguez‐Cabal , E. T. Wu , et al. 2015. “Multiple Plant Traits Shape the Genetic Basis of Herbivore Community Assembly.” Functional Ecology 29: 995–1006.

[ele70039-bib-0007] Barrett, R. D. H. , and D. Schluter . 2008. “Adaptation From Standing Genetic Variation.” Trends in Ecology & Evolution 23: 38–44. 18006185 10.1016/j.tree.2007.09.008

[ele70039-bib-0080] Barton, K. 2019. “MuMIn: Multi‐Model Inference.” R Package. http://r‐forge.r‐project.org/projects/mumin/.

[ele70039-bib-0008] Benning, J. W. , and D. A. Moeller . 2019. “Maladaptation Beyond a Geographic Range Limit Driven by Antagonistic and Mutualistic Biotic Interactions Across an Abiotic Gradient.” Evolution 73: 2044–2059. 31435931 10.1111/evo.13836

[ele70039-bib-0009] Bingham, R. A. , and A. A. Agrawal . 2010. “Specificity and Trade‐Offs in the Induced Plant Defence of Common Milkweed *Asclepias syriaca* to Two Lepidopteran Herbivores.” Journal of Ecology 98: 1014–1022.

[ele70039-bib-0010] Bont, Z. , T. Züst , C. C. M. Arce , M. Huber , and M. Erb . 2020. “Heritable Variation in Root Secondary Metabolites Is Associated With Recent Climate.” Journal of Ecology 108: 2611–2624.

[ele70039-bib-0011] Bozzuto, C. , I. Biebach , S. Muff , A. R. Ives , and L. F. Keller . 2019. “Inbreeding Reduces Long‐Term Growth of Alpine Ibex Populations.” Nature Ecology & Evolution 3: 159–1364. 31477848 10.1038/s41559-019-0968-1

[ele70039-bib-0012] Campbell, D. R. 2019. “Early Snowmelt Projected to Cause Population Decline in a Subalpine Plant.” Proceedings of the National Academy of Sciences 116: 12901–12906. 10.1073/pnas.1820096116 PMC6600911 31182600

[ele70039-bib-0013] Carley, L. N. , J. P. Mojica , B. Wang , et al. 2021. “Ecological Factors Influence Balancing Selection on Leaf Chemical Profiles in a Wildflower.” Nature Ecology & Evolution 5: 1135–1144. 34140651 10.1038/s41559-021-01486-0 PMC8325631

[ele70039-bib-0014] Carley, L. N. , W. F. Morris , D. Riebe , R. Walsh , and T. Mitchell‐Olds . 2022. “Are Genetic Variation and Demographic Performance Linked?” Evolutionary Applications 15: 1888–1906. 36426131 10.1111/eva.13487 PMC9679243

[ele70039-bib-0015] Caswell, H. 2001. Matrix Population Models: Construction, Analysis, and Interpretation. United Kingdom: Sinauer Associates.

[ele70039-bib-0016] Chown, S. L. , and K. J. Gaston . 1999. “Exploring Links Between Physiology and Ecology at Macro‐Scales: The Role of Respiratory Metabolism in Insects.” Biological Reviews 74: 87–120.

[ele70039-bib-0017] Doak, D. , and W. F. Morris . 2010. “Demographic Compensation and Tipping Points in Climate‐Induced Range Shifts.” Nature 467: 959–962. 20962844 10.1038/nature09439

[ele70039-bib-0018] Ehrlén, J. 2003. “Fitness Components vs. Total Demographic Effects: Evaluating Herbivore Impacts on a Perennial Herb.” American Naturalist 162: 796–810. 10.1086/379350 14737716

[ele70039-bib-0019] Ehrlén, J. , and Z. Münzbergová . 2009. “Timing of Flowering: Opposed Selection on Different Fitness Components and Trait Covariation.” American Naturalist 173: 819–830. 10.1086/598492 19335224

[ele70039-bib-0020] Ellner, S. P. , and M. Rees . 2006. “Integral Projection Models for Species With Complex Demography.” American Naturalist 167: 410–428. 10.1086/499438 16673349

[ele70039-bib-0021] Etterson, J. R. , and R. G. Shaw . 2001. “Constraint to Adaptive Evolution in Response to Global Warming.” Science 294: 151–154. 11588260 10.1126/science.1063656

[ele70039-bib-0022] Galdon‐Armero, J. , M. Fullana‐Pericas , P. A. Mulet , M. A. Conesa , C. Martin , and J. Galmes . 2018. “The Ratio of Trichomes to Stomata Is Associated With Water Use Efficiency in *Solanum lycopersicum* (Tomato).” Plant Journal 96: 607–619. 10.1111/tpj.14055 PMC6321981 30066411

[ele70039-bib-0081] Garcia, L. C. , and M. D. Eubanks . 2019. “Overcompensation for Insect Herbivory: A Review and Meta‐Analysis of the Evidence.” Ecology 100, no. 3: e02585. 10.1002/ecy.2585. 30554427

[ele70039-bib-0023] Gómez, J. M. 2008. “Sequential Conflicting Selection due to Multispecific Interactions Triggers Evolutionary Trade‐Offs in a Monocarpic Herb.” Evolution 62: 668–679. 18182075 10.1111/j.1558-5646.2007.00312.x

[ele70039-bib-0024] Hahn, P. G. , A. A. Agrawal , K. I. Sussman , and J. L. Maron . 2019. “Population Variation, Environmental Gradients, and the Evolutionary Ecology of Plant Defense Against Herbivory.” American Naturalist 193: 20–34. 10.1086/700838 30624107

[ele70039-bib-0025] Hajjar, R. , D. I. Jarvis , and B. Gemmill‐Herren . 2008. “The Utility of Crop Genetic Diversity in Maintaining Ecosystem Services.” Agriculture, Ecosystems & Environment 123: 261–270.

[ele70039-bib-0026] Hamann, E. , C. Blevins , S. J. Franks , M. Inam Jameel , and J. T. Anderson . 2020. “Climate Change Alters Plant‐Herbivore Interactions.” New Phytologist 229: 1894–1910. 33111316 10.1111/nph.17036

[ele70039-bib-0027] Hanski, I. , and I. Saccheri . 2006. “Molecular‐Level Variation Affects Population Growth in a Butterfly Metapopulation.” PLoS Biology 4: e129. 16620151 10.1371/journal.pbio.0040129 PMC1440940

[ele70039-bib-0028] Hedrick, P. W. 1976. “Genetic Variation in a Heterogeneous Environment. II. Temporal Heterogeneity and Directional Selection.” Genetics 84: 145–157. 992363 10.1093/genetics/84.1.145 PMC1213561

[ele70039-bib-0029] Hedrick, P. W. 1986. “Genetic Polymorphism in Heterogeneous Environments: A Decade Later.” Annual Review of Ecology and Systematics 17: 535–566.

[ele70039-bib-0030] Hedrick, P. W. 1999. “Antagonistic Pleiotropy and Genetic Polymorphism: A Perspective.” Heredity 82: 126–133.

[ele70039-bib-0031] Higgins, R. W. , and W. Shi . 2001. “Intercomparison of the Principal Modes of Interannual and Intraseasonal Variability of the North American Monsoon System.” Journal of Climate 14: 403–417.

[ele70039-bib-0032] Hopkins, R. J. , N. M. van Dam , and J. J. A. van Loon . 2009. “Role of Glucosinolates in Insect‐Plant Relationships and Multitrophic Interactions.” Annual Review of Entomology 54: 57–83. 10.1146/annurev.ento.54.110807.090623 18811249

[ele70039-bib-0033] Hossain, M. S. , W. Ye , M. A. Hossain , et al. 2013. “Glucosinolate Degradation Products, Isothiocyanates, Nitriles, and Thiocynates, Induce Stomatal Closure Accompanied by Peroxidase‐Mediated Reactive Oxygen Species Production in *Arabidopsis thaliana* .” Bioscience, Biotechnology, and Biochemistry 77: 977–983. 23649257 10.1271/bbb.120928

[ele70039-bib-0034] Hughes, A. R. , and J. J. Stachowicz . 2004. “Genetic Diversity Enhances the Resistance of a Seagrass System to Disturbance.” Proceedings of the National Academy of Sciences 101: 8998–9002. 10.1073/pnas.0402642101 PMC428461 15184681

[ele70039-bib-0035] Iason, G. R. , J. M. O'Reilly‐Wapstra , M. J. Brewer , R. W. Summers , and B. D. Moore . 2011. “Do Multiple Herbivores Maintain Chemical Diversity of Scots Pine Monoterpenes?” Philosophical Transactions of the Royal Society, B: Biological Sciences 366: 1337–1345. 10.1098/rstb.2010.0236 PMC3081569 21444308

[ele70039-bib-0036] Johnson, M. T. J. , M. Velland , and J. R. Stinchcombe . 2009. “Evolution in Plant Populations as a Driver of Ecological Changes in Arthropod Communities.” Philosophical Transactions of the Royal Society B 364: 1593–1605. 10.1098/rstb.2008.0334 PMC2690498 19414473

[ele70039-bib-0037] Lande, R. , and S. Shannon . 1996. “The Role of Genetic Variation in Adaptation and Population Persistence in a Changing Environment.” Evolution 50: 434–437. 28568879 10.1111/j.1558-5646.1996.tb04504.x

[ele70039-bib-0038] Lin, P.‐A. , J. Kansman , W.‐P. Chuang , C. Robert , M. Erb , and G. W. Felton . 2023. “Water Availability and Plant‐Herbivore Interactions.” Journal of Experimental Botany 74: 2811–2828. 36477789 10.1093/jxb/erac481

[ele70039-bib-0039] Louthan, A. M. , R. M. Pringle , J. R. Goheen , T. M. Palmer , W. F. Morris , and D. F. Doak . 2018. “Aridity Weakens Population‐Level Effects of Multiple Species Interactions on *Hibiscus meyeri* .” Proceedings of the National Academy of Sciences 115: 543–548. 10.1073/pnas.1708436115. PMC5776961 29284748

[ele70039-bib-0040] Merow, C. , J. P. Dahlgren , C. J. E. Metcalf , et al. 2014. “Advancing Population Ecology With Integral Projection Models: A Practical Guide.” Methods in Ecology and Evolution 5: 99–110.

[ele70039-bib-0041] Metcalf, J. C. E. , and S. Pavard . 2007. “Why all Evolutionary Biologists Should Be Demographers.” Trends in Ecology & Evolution 22: 205–212. 17174004 10.1016/j.tree.2006.12.001

[ele70039-bib-0042] Mojica, J. P. , and J. K. Kelly . 2010. “Viability Selection Prior to Trait Expression Is an Essential Component of Natural Selection.” Proceedings of the Royal Society B: Biological Sciences 277: 2945–2950. 10.1098/rspb.2010.0568 PMC2982025 20462906

[ele70039-bib-0043] Monroe, J. G. , H. Cai , and D. L. Des Marais . 2021. “Diversity in Nonlinear Responses to Soil Moisture Shapes Evolutionary Constraints in *Brachypodium* .” G3: Genes, Genomes, Genetics 11: jkab334. 34570202 10.1093/g3journal/jkab334 PMC8664479

[ele70039-bib-0077] Morris, W. F. , J. Ehrlén , J. P. Dahlgren , A. K. Loomis , and A. M. Louthan . 2020. “Biotic and Anthropogenic Forces Rival Climatic/Abiotic Factors in Determining Global Plant Population Growth and Fitness.” Proceedings of the National Academy of Sciences of the United States of America 117, no. 2: 1107–1112. 10.1073/pnas.1918363117. 31888999 PMC6969536

[ele70039-bib-0044] Nguyen, D. , I. Rieu , C. Mariani , and N. M. van Dam . 2016. “How Plants Handle Multiple Stresses: Hormonal Interactions Underlying Responses to Abiotic Stress and Insect Herbivory.” Plant Molecular Biology 91: 727–740. 27095445 10.1007/s11103-016-0481-8 PMC4932144

[ele70039-bib-0045] O'Connell, R. D. , D. F. Doak , C. C. Horvitz , J. B. Pascarella , and W. F. Morris . 2024. “Nonlinear Life Table Response Experiment Analysis: Decomposing Nonlinear and Nonadditive Population Growth Responses to Changes in Environmental Drivers.” Ecology Letters 27: e14417. 38549264 10.1111/ele.14417

[ele70039-bib-0046] Oldfather, M. F. , M. J. Koontz , D. F. Doak , and D. A. Ackerly . 2021. “Range Dynamics Mediated by Compensatory Life Stage Responses to Experimental Climate Manipulations.” Ecology Letters 24: 772–780. 33559296 10.1111/ele.13693

[ele70039-bib-0047] Pezzola, E. , S. Mancuso , and R. Karban . 2017. “Precipitation Affects Plant Communication and Defense.” Ecology 98: 1693–1699. 28376291 10.1002/ecy.1846

[ele70039-bib-0048] Poelman, E. H. , J. J. A. van Loon , and M. Dicke . 2008. “Consequences of Variation in Plant Defense for Biodiversity at Higher Trophic Levels.” Trends in Plant Science 13: 534–541. 18774329 10.1016/j.tplants.2008.08.003

[ele70039-bib-0049] Prasad, K. V. S. K. , B.‐H. Song , C. Olson‐Manning , et al. 2012. “A Gain‐Of‐Function Polymorphism Controlling Complex Traits and Fitness in Nature.” Science 337: 1081–1084. 22936775 10.1126/science.1221636 PMC3872477

[ele70039-bib-0050] Raffard, A. , F. Santoul , J. Cucherousset , and S. Blanchet . 2019. “The Community and Ecosystem Consequences of Intraspecific Diversity: A Meta‐Analysis.” Biological Reviews 94: 648–661. 30294844 10.1111/brv.12472

[ele70039-bib-0051] Rasmann, S. , L. Pellissier , E. Defossez , H. Jactel , and G. Kunstler . 2014. “Climate‐Driven Change in Plant–Insect Interactions Along Elevation Gradients.” Functional Ecology 28: 46–54.

[ele70039-bib-0052] Reusch, T. B. , and A. R. Hughes . 2005. “The Emerging Role of Genetic Diversity for Ecosystem Functioning: Estuarine Macrophytes as Models.” Estuaries and Coasts 29: 159–164.

[ele70039-bib-0053] Reznick, D. N. 2013. “A Critical Look at Reciprocity in Ecology and Evolution: Introduction to the Symposium.” American Naturalist 181: S1–S8. 10.1086/670030 23598355

[ele70039-bib-0054] Rotter, M. C. , K. Christie , and L. M. Holeski . 2022. “Climate and the Biotic Community Structure Plant Resistance Across Biogeographic Groups of Yellow Monkeyflower.” Ecology and Evolution 12: e9520. 36440318 10.1002/ece3.9520 PMC9682197

[ele70039-bib-0055] Rushworth, C. A. , B.‐H. Song , C.‐R. Lee , and T. Mitchell‐Olds . 2011. “ *Boechera*, a Model System for Ecological Genomics.” Molecular Ecology 20: 4843–4857. 22059452 10.1111/j.1365-294X.2011.05340.x PMC3222738

[ele70039-bib-0056] Rushworth, C. A. , M. R. Wagner , T. Mitchell‐Olds , and J. T. Anderson . 2022. “The *Boechera* Model System for Evolutionary Ecology.” American Journal of Botany 109: 1939–1961. 36371714 10.1002/ajb2.16090

[ele70039-bib-0057] Salazar, D. , and R. J. Marquis . 2012. “Herbivore Pressure Increases Toward the Equator.” Proceedings of the National Academy of Sciences 109: 12616–12620. 10.1073/pnas.1202907109 PMC3411992 22802664

[ele70039-bib-0058] Salehin, M. , B. Li , M. Tang , et al. 2019. “Auxin‐Sensitive Aux/IAA Proteins Mediate Drought Tolerance in *Arabidopsis* by Regulating Glucosinolate Levels.” Nature Communications 10: 4021. 10.1038/s41467-019-12002-1 PMC6731224 31492889

[ele70039-bib-0059] Schluter, D. , T. D. Price , and R. Locke . 1991. “Conflicting Selection Pressures and Life History Trade‐Offs.” Proceedings of the Royal Society of London B: Biological Sciences 246: 11–17.

[ele70039-bib-0060] Schranz, M. E. , A. Manzaneda , A. Windsor , M. J. Clauss , and T. Mitchell‐Olds . 2009. “Ecological Genomics of *Boechera stricta* : Identification of a QTL Controlling the Allocation of Methionine‐ Vs Branched‐Chain Amino Acid‐Derived Glucosinolates and Levels of Insect Herbivory.” Heredity 102: 465–474. 19240753 10.1038/hdy.2009.12 PMC2775550

[ele70039-bib-0061] Seth, A. , S. A. Rauscher , M. Rojas , A. Giannini , and S. J. Camargo . 2011. “Enhanced Spring Convective Barrier for Monsoons in a Warmer World?” Climatic Change 104: 403–414.

[ele70039-bib-0063] Sheth, S. N. , and A. L. Angert . 2018. “Demographic Compensation Does Not Rescue Populations at a Trailing Range.” Proceedings of the National Academy of Sciences 115: 2413–2418. 10.1073/pnas.1715899115. PMC5878003 29463711

[ele70039-bib-0064] Shriver, R. K. 2015. “Quantifying How Short‐Term Environmental Variation Leads to Long‐Term Demographic Responses to Climate Change.” Journal of Ecology 104: 65–78.

[ele70039-bib-0065] Sloat, L. L. , A. N. Henderson , C. Lamnna , and B. J. Enquist . 2015. “The Effect of Foresummer Drought on Carbon Exchange in Subalpine Meadows.” Ecosystems 18: 533–545.

[ele70039-bib-0066] Song, B.‐H. , M. J. Clauss , A. Pepper , and T. Mitchell‐Olds . 2006. “Geographic Patterns of Microsatellite Variation in *Boechera stricta*, a Close Relative of *Arabidopsis* .” Molecular Ecology 15: 357–369. 16448406 10.1111/j.1365-294X.2005.02817.x

[ele70039-bib-0067] Stinchcombe, J. R. , and M. D. Rausher . 2002. “The Evolution of Tolerance to Deer Herbivory: Modifications Caused by the Abundance of Insect Herbivores.” Proceedings of the Royal Society of London B 269: 1241–1246. 10.1098/rspb.2002.2015 PMC1691020 12065040

[ele70039-bib-0068] Strauss, S. Y. , J. A. Rudgers , J. A. Lau , and R. E. Erwin . 2002. “Direct and Ecological Costs of Resistance to Herbivory.” Trends in Ecology & Evolution 17: 278–285.

[ele70039-bib-0069] Sun, J. Y. , I. E. Sønderby , B. A. Halkier , G. Jander , and M. de Vos . 2009. “Non‐Volatile Intact Indole Glucosinolates Are Host Recognition Cues for Ovipositing *Plutella xylostella* .” Journal of Chemical Ecology 35: 1427–1436. 20054620 10.1007/s10886-009-9723-4

[ele70039-bib-0070] Tariq, M. , J. T. Rossiter , D. J. Wright , and J. T. Staley . 2013. “Drought Alters Interactions Between Root and Foliar Herbivores.” Oecologia 172: 1095–1104. 23292454 10.1007/s00442-012-2572-9

[ele70039-bib-0071] Thompson, J. N. 1998. “Rapid Evolution as an Ecological Process.” Trends in Ecology & Evolution 13: 329–332. 21238328 10.1016/s0169-5347(98)01378-0

[ele70039-bib-0072] van Loon, J. J. A. , A. Blaakmeer , F. C. Griepink , T. A. van Beek , L. M. Schoonhoven , and A. de Groot . 1992. “Leaf Surface Compound From *Brassica oleracea* (Cruciferae) Induces Oviposition by *Pieris brassicae* (Lepidoptera: Pieridae).” Chemoecology 3: 39–44.

[ele70039-bib-0073] Wadgymar, S. M. , S. Sheth , E. Josephs , M. DeMarche , and J. Anderson . 2024. “Defining Fitness in Evolutionary Ecology.” International Journal of Plant Sciences 185: 218–227. 39035046 10.1086/729360 PMC11257499

[ele70039-bib-0074] Wan, N.‐F. , L. Fu , M. Dainese , et al. 2022. “Plant Genetic Diversity Affects Multiple Trophic Levels and Trophic Interactions.” Nature Communications 13: 7312. 10.1038/s41467-022-35087-7 PMC9701765 36437257

[ele70039-bib-0078] Yahdjian, L. , and O. E. Sala . 2002. “A Rainout Shelter Design for Intercepting Different Amounts of Rainfall.” Oecologia 133, no. 2: 95–101. 10.1007/s00442-002-1024-3. 28547315

[ele70039-bib-0079] Zeileis, A. , and T. Hothorn . 2002. “Diagnostic Checking in Regression Relationships.” R News 2, no. 3: 7–10. https://CRAN.R‐project.org/doc/Rnews/.

[ele70039-bib-0075] Züst, T. , C. Heichinger , U. Grossniklaus , R. Harrington , D. J. Kliebenstein , and L. A. Turnbull . 2012. “Natural Enemies Drive Geographic Variation in Plant Defenses.” Science 338: 116–119. 23042895 10.1126/science.1226397

[ele70039-bib-0076] Züst, T. , S. Rasmann , and A. A. Agrawal . 2015. “Growth‐Defense Tradeoffs for Two Major Anti‐Herbivore Traits of the Common Milkweed *Asclepias syriaca* .” Oikos 124: 1404–1415.

