# Supplementary material for: Increasing Aridity May Threaten the Maintenance of a Plant Defence Polymorphism
Source: Ecol Lett. 2024 Dec 31;28(1):e70039. doi: 10.1111/ele.70039 (PMC11687350; doi:10.1111/ele.70039)
Supplement: Supplementary file 1 — Data S1. [file ELE-28-0-s001.pdf]

Supporting Information

Increasing aridity may threaten the maintenance of a plant defense polymorphism

Lauren N. Carley*, Tom Mitchell-Olds, and William F. Morris
*lcarley@uchicago.edu

Contents:

Appendix S1: Detailed field methods……………………………………………………….2
Appendix S2: Detailed demographic modeling methods……………………………………6
Appendix S3: Outcomes of demographic model selection……………………………….. 16
Supplementary Figures………………………………………………………………………. 35
Supplementary Tables………………………………………………………………………...49
References occurring in the Supporting Information……………………………………… 56

Appendix S1: Detailed field methods

Genetic materials

Independent crossing lineages (i.e. “family lines”) nested within NIL genotype accounted for possible effects of rare segregating loci outside of the *BCMA1/3* region (Carley et al. 2021). In each field block, the 25 replicates per genotype were distributed evenly across independent family lines (**Supplementary Table 1**).

Plant care

Prior to transplanting in the field, we reared juvenile plants in the greenhouse at Duke University (Durham, NC) under uniform conditions, as previously described (Carley et al. 2021). Greenhouse conditions were as follows: 18–21 °C daytime temperature; 13–16 °C nighttime temperature; 16 h day length; watering to saturation daily; fertilization at 300 ppm N weekly. Plants were grown for ~8 weeks post-germination under greenhouse conditions before shipping to the field for transplanting.

Rainout shelters

Using 0.5” diameter PVC pipes, we constructed rectangular prism-shaped frames sized to cover one randomized complete block of 50 transplants plus a 10 cm buffer around the perimeter (**Supplementary Figure 12**). Rectangular faces for the top and bottom of the frames were 120 cm × 70 cm in size, and the faces were connected with vertical pipes 100 cm tall on one side and 110 cm tall on the other. This creates a slanted surface above each garden block. We fitted the slanted upper surface of the rectangular prism with clear corrugated polycarbonate slats (Suntuf®, Palram Americas Ltd., Kutztown, PA) to intercept rainfall above 50% of the surface area of each block. To reduce lateral flow of moisture among blocks, we also installed strips of 6 mil thickness (~0.15 mm) polyethylene film (Moisture Barricade, Roberts Consolidated Industries Inc., Boca Raton, FL) vertically to 10 cm depth in narrow trenches around the perimeter of each rainout shelter.

Following shelter deployment in 2017 and 2018, we tracked the efficacy of the reduced rainfall treatment by measuring soil moisture (volumetric water content, i.e., VWC) every 2-4 days at three points within each garden block for the duration of the growing season, using a Hydrosense™ handheld soil moisture measurement system (Campbell Scientific Inc., Logan, UT) with 12 cm probes. To monitor potential non-target effects of the shelters, we also measured soil temperature in the center of each garden block every 2-4 days during the 2017 and 2018 growing seasons using a probe thermometer. Finally, we measured light transmission at ground level in each garden block on three dates in 2017 using a handheld quantum sensor (MQ-200, Apogee Instruments, Logan, UT).

Inferring unobserved soil moisture data

To build driver-sensitive demographic models, we sought to include estimates of both soil moisture and herbivore pressure as predictors of survival, growth, and reproduction during each year of the experiment. However, due to logistical constraints, we were unable to measure soil moisture in garden blocks during the 2019 growing season. Therefore, we combined our garden-specific measurements of soil moisture from 2017 and 2018 with similar data from a nearby field site 0.5 km NW (**Supplementary Figures 2-3;** J. T. Anderson, *unpubl. data*) to predict unobserved block-level measurements of soil moisture in 2016 and 2019, as well as on unmeasured days in 2017 and 2018. Relative consistency of block-level soil moisture across years within our experimental garden justifies this decision (**Supplementary Figure 1**)

To fit a predictive relationship between off-site garden mean soil moisture and block-specific values in our garden, we focused on data from the 2018 field season, which had the best temporal resolution and range in our garden. For off-site data, we first calculated a daily garden mean value from continuous datalogger measurements taken at 15-minute intervals in 5 locations at 10 cm depth under ambient environmental conditions (Decagon 5TM sensors, now ECH20 5TM, Meter Environment, Pullman, WA; J. T. Anderson, *unpubl. data*). For our direct in-garden measurements, we calculated block-level means from three measurements per block on each observation day. We then paired the off-site and block-level means by observation day. We used a linear model to regress our observed daily block-level means upon the off-site daily means, accounting for a fixed effect of garden block and a block × off-site mean interaction. This approach yielded block-specific coefficient estimates that relate block-level treatment and microhabitat conditions in our experimental garden to off-site mean values. Off-site means significantly predicted in-garden values (F = 3,703.88, $P < 2.2 \times 10^{-16}$, N = 1,180), and in-garden blocks varied significantly in both the intercepts (garden block main effect: F = 10.33, $P < 2.2 \times 10^{-16}$) and slopes (block × off-site interaction: F = 1.07, $P < 2.2 \times 10^{-16}$) describing that relationship. The full model explained approximately 80% of the variance in the observed in-garden block level measurements (multiple R^2 = 0.8001, adjusted R^2 = 0.7834).

Because we directly measured soil moisture in our experimental garden in 2017 as well, we tested the accuracy of our 2018-parameterized predictive model against observed data in 2017. A univariate linear model regressing observed upon predicted values revealed a strong positive correlation among them (r = 0.7654, F = 908.55, $P < 2.2 \times 10^{-16}$), although visualizing the relationship revealed that the model tended to over-predict block-level soil moisture (**Supplementary Figure 13**). Adding a constant value ($\beta 0_{\text{year}}$) to the intercept equal to the growing season mean VWC deviation from the 2018 growing season mean centered the observed vs. predicted values around the 1:1 line (**Supplementary Figure 13**). As such, we ultimately predicted daily unobserved values using the following general formula, for each block b in each year y:

$$VWC_{block,year} = \beta 0_{year} + \beta 0_{block} + \beta 1_{block} \times VWC_{Mean}$$

where VWC_{Mean} is the off-site daily mean VWC from Anderson et al. (*unpubl. data*; **Supplementary Figure 2**). We used the same formula to predict unobserved values during the 2016 growing season; however, in that season, we added back a constant value to blocks assigned to the rainout treatment equal to the mean difference in VWC among rainout and control plots in the 2017 and 2018 observations. In this way, we estimated block-level variation in soil moisture prior to the start of the environmental manipulations, which began in 2017.

This approach yielded daily estimates of block-level VWC variation that were concordant with observed values, but at higher and more even temporal resolution, and expanded to include estimated values for the 2016 and 2019 growing seasons (**Supplementary Figure 3**). From these predicted values, we calculated the block-level mean VWC across the growing season (spanning from snowmelt date through September 1; **Supplementary Table 2**). We used these block-specific annual growing season means as a drought-related environmental driver in our demographic models.

Herbivory manipulations

Because some plants died prior to the start of manipulations, we censused early-season survival in 2017 to determine the number of surviving individuals per genotype and per block at the start of environmental manipulations (**Supplementary Table 3**). Then, within each block, we randomly assigned 50% of the surviving individuals of each genotype to ambient or reduced herbivory treatments (**Supplementary Table 4**). We marked each individual in the reduced herbivory treatment with a plastic toothpick to identify treatment assignments within the randomized garden blocks throughout the growing season. Insecticide treatment application methods are described in the main text.

Plant measurements

During censuses, we scored each individual as alive or dead, and measured the height of each living plant (for juvenile plants, the longest leaf length; for reproductive individuals, the height of the flowering stalk). We scored living plants for their life-history stage, which we binned into reproductive (had produced flowers or fruits) or non-reproductive (no flowers or fruits) categories. For plants with fruits, we recorded the number and average length of fruits to estimate total fruit size.

We measured herbivore damage by counting the total number of leaves and the number of damaged leaves per plant and estimating the average percent damage per damaged leaf in 5% bins. We used these raw data to estimate total proportion leaf area removed (LAR) by herbivores as follows (*sensu* Carley et al. 2021):

$$\%\,LAR = \frac{\#\,damaged\ leaves}{\#\,leaves} \times\ average\ \%\ damage\ per\ damaged\ leaf$$

Estimating total reproductive output

Our NIL genotypes are derived from crosses containing genetic material from a local Colorado population as well as a distant Idaho population (Carley et al. 2021). Because of this, our research permit precluded us from allowing the fruits of experimental plants to mature and disperse seeds. Thus, to estimate reproductive output in the demographically relevant unit of total seeds per plant, we used direct counts of mature seeds per plant from a different *B. stricta* common garden experiment conducted at a nearby field site 0.5 km NW of our garden (Anderson 2019a) and measurements of fruit number and length from the same individuals (J. T. Anderson, *unpubl. data*). We modeled seed number in response to total fruit length (the sum of the lengths of all fruits) using a univariate linear model. Total fruit length was strongly correlated with seed number in this location ($r = 0.742$; $P = 1.03 \times 10^{-12}$, N = 66). We used the fitted regression to predict seed number per plant in our own experimental transplants using the following equation, where fruit length is measured in cm:

$$Seeds = -0.3984 + 10.2584 \times total\ fruit\ length$$

We rounded predicted values to the nearest integer and censored any negative predictions to 0 if necessary.

Appendix S2: Detailed demographic modeling methods

Vital rate regressions and model selection

Data handling

Block effects

Because the resolution of our soil moisture measurements was at the block level and thus confounded with block identity, we did not include block as a random effect in vital rate models.

Year effects

We chose not to include year as a model effect in our vital rate regressions, instead pooling data across years. Mean soil moisture and herbivory varied significantly across years during our study. We were predominantly interested in the net effects of this variation in drivers on vital rates, not the residual block-to-block variation in soil moisture after accounting for interannual variation. Because of this, we could not include year as a fixed effect in vital rate regressions. With only four annual transitions represented in our demographic dataset, this is a relatively small number of years to account for as a random effect. Finally, we had no *a priori* hypotheses about whether year ought to be modeled as affecting only intercepts vs. intercepts and slopes of vital rates. With vital rate regressions already including interactions between up to three continuous variables (drought, herbivory, and plant size), including additional model effects may cause overfitting, and make interpretation of model selection more complicated.

State-specific vital rates

Each individual's bolting history strongly influenced growth patterns (**Supplementary Figure 8**). As such, for individuals of a given size experiencing a given level of herbivory and soil moisture, we modeled the probability of bolting with separate functions for plants that had or had not bolted previously. Similarly, we modeled growth and variance in growth with distinct size- and driver-dependent functions for each of four bolting histories. We then used model selection, as described in the main text, to determine the best-fit vital rate regression for each genotype in each of these discrete bolting state-spaces (**Supplementary Table 5; Appendix S3**). Linear models for growth had Normally-distributed residuals.

Reproductive vital rates

Because the various components of reproduction happen sequentially, we modeled each reproduction-related vital rate as conditional upon the achievement of the preceding component. Specifically, we fit models for the probability of reproduction conditional upon bolting, using only individuals that successfully bolted. Similarly, we modeled seed set as conditional upon reproduction using only individuals that successfully produced fruits.

Juvenile vital rates

Because we were required to prevent incursion of non-local genotypes through seed dispersal, we used transplanted rosettes in the experiment, and thus did not directly estimate germination, seed dormancy, or germinant survival in the field. To account for these life-history transitions in our model, we used size-, driver-, and genotype-independent values previously reported in another demographic study on transplanted *B. stricta* in nearby sites.

A prior study reported seed dormancy estimates for transplanted seeds in five common gardens around Gunnison Valley, CO (Anderson 2019b). That experiment estimated dormancy in these sites using seeds from local populations in each test site. One of the test sites was nearby to our common garden; however, dormancy rates observed in the most nearby site were anomalous compared to the other four test sites, and the authors of the other study interpret them with caution. Thus, in our model, we used the average of dormancy estimates from all other four test sites (0.0318) as the proportion of seeds produced that would remain dormant in the seed bank for one year.

To estimate germination, we extracted previously published data from one common garden experiment conducted 0.5 km NW of our study (Anderson 2019b). This dataset reports a total of 138 germinants arising from 1220 transplanted seeds in the test site nearest to our transplant garden. These seeds were sourced from 5 populations in the same general region as our transplant garden, but spanning an elevation gradient from ~6000' to ~11000'. We pooled all data across source populations and estimated the mean germination proportion as 0.113. The same study (Anderson & Wadgymar 2020, Anderson 2019b) reported that 89 of the 138 germinants survived until the end of the second growing season (one year following seed dispersal), across 5 seed sources transplanted into their nearby common garden. We used these pooled data across source populations to estimate the mean germinant survival proportion as 0.645.

Some of these previously published data come from experiments involving manipulation of snowpack and/or precipitation. For datasets indicating these experimental treatments, we only used data from control plots (ambient environmental conditions), since snowpack and precipitation manipulations in those experiments do not exactly match rainfall or herbivory manipulations implemented in this study.

Recruit size

For propagules that successfully germinated and established, we assigned new recruits to juvenile size values drawn from genotype-specific Normal distributions. We parameterized these distributions by calculating the mean and standard deviation of plant size for juvenile transplants at the time of transplanting in the field (approx. 2 months old, similar to the age of new recruits at the end of the first growing season following spring germination).

Summary

An overview of the vital rates parameterized in this study is provided in **Supplementary Tables 5-6**. A visual representation, including indications of which vital rates are modeled as size- and driver-dependent and which are not, is provided in **Supplementary Figure 14**.

Integral projection model

We provide a brief narrative overview of our IPM construction, with full mathematical equations provided below.

Overview of multi-matrix structure

We applied state-specific vital rate functions for growth and variance in growth to the *G* megamatrix. Because survival did not vary depending on state, we applied the same survival functions across all sub-matrices of the *S* megamatrix. We used state-specific bolting functions to populate a bolting megamatrix that models the probability of individual plants transitioning from one discrete bolting state to another. We then calculated the full survival/growth kernel as the product of these three megamatrices.

Reproduction did not vary considerably depending on past bolting history, but individuals that do not bolt in the current year cannot reproduce. So, we applied our fecundity-related vital rate functions to the top half of our fecundity megamatrix and populated the lower half with 0s. As with our growth/survival megamatrix, we multiplied the survival/growth kernel by our bolting matrix to account for transitions of individuals from one discrete bolting state to another. Finally, we calculated the full IPM kernel as the sum of the survival-growth-bolting and the fecundity megamatrices, each multiplied by the bin width.

Kernel construction

Because of state-dependency of some vital rates (see above), we modeled λ using a population vector separately estimating the contributions of previously bolting vs. non-bolting (*b*) individuals to each lineage's growth rate. Following Merow et al. (2014) and Ellner & Rees (2006), we incorporated all vital rates (**Supplementary Table 5**) into an IPM using two state-specific kernels. The survival and growth kernel predicts the number of individuals of size *z'* at time *t*+1 produced by individuals of size *z* in the population at time *t*. We also modeled growth and survival dependent on herbivory and soil moisture.

Our fecundity kernel integrated life-history transitions regarding reproduction and recruitment: the probability of bolting, the probability of fruiting given bolting, the number of seeds produced given reproduction, the germination rate, the dormancy rate, the germinant survival rate, and the size distribution of established recruits. The first three transitions were size- and driver-dependent, while the latter four were size- and driver-independent. In other words, the net recruitment of new individuals at time *t*+1 is modeled as a function of all seeds produced plus seeds that remain viable in the seed bank multiplied by the probability of germinating and surviving the first year of growth

and the probability of reaching size z' in the first year. Following Anderson & Wadgymar (2020), we allow seeds to persist in the seed bank for one year. Germinants that survive and establish are assigned a size using a genotype-specific Normal distribution (renormalized to avoid downward eviction).

Evaluation of the IPM

We evaluated the kernels over size ranges spanning from 0.9× the minimum size through 1.1× the maximum size values observed in our longitudinal dataset. We defined the number of kernel meshpoints as 100, yielding a meshpoint width of approximately 0.6 cm. In other words, while our vital rate regressions were parameterized using continuous data, we used a discretized matrix representation of the continuous kernel to compute population growth rate. We evaluated the IPM at higher and lower numbers of kernel meshpoints to ensure that our outcomes were not sensitive to the meshpoint resolution; outcomes at different meshpoint resolutions were identical. We applied vital rate functions across the meshpoints and state spaces as described above. For any individuals whose growth was projected to exceed the size bounds of the model, we corrected for "eviction" by re-normalizing each column of each growth sub-matrix (i.e., dividing the values of each row in a column by the column sum). To incorporate the one-year seed bank, we added to the matrix that is the discretized version of the IPM kernel an extra row at top and column at left to reflect input into and outflow from the seed bank, respectively. We then took the dominant eigenvalue of this expanded matrix as the lineage growth rate (λ).

Because our vital rates were modeled as driver-dependent, we evaluated each IPM across a biologically relevant range of herbivory and soil moisture values: 0-100% aboveground tissue removal by herbivores, and 0 through 1.25× the maximum observed soil moisture value across the 3-year experiment. We discretized these driver ranges into 25 bins (equal bin width for soil moisture; log-scale bin width for herbivory, to better reflect the distribution of herbivory values observed in the field; **Supplementary Figure 7**), and estimated λ in each of these 25×25=625 environmental combinations. We tested outcomes inferred by modeling driver variation at coarser and finer scales, and found that patterns were quantitatively similar and qualitatively identical at driver resolutions higher than 25×25, so we chose this increment as a reasonable tradeoff between environmental resolution and computation time.

Because some modeled driver values exceeded those observed empirically during the study, and because variance in growth was modeled as size- and driver-dependent, it is possible at some driver values to predict that growth variance is 0 or negative (e.g. if driver values are 0 and there is a strong driver-by-size interaction influencing variance in growth). To account for this, we set a minimum variance level of 0.01; if vital rate regressions predicted variance lower than this, those values were overridden by this minimum value. We tested the outcomes inferred from models using higher and lower minimum variance tolerances (0.1, 0.001) and found patterns to be quantitatively similar and qualitatively identical.

IPM equations

Notation rules

- Single letters are used for each vital rate function
- Subscripts are used for discrete classifying variables (bolting states and time)
- Function notation (e.g., $f(z)$) is used to indicate that a vital rate is a function of a continuous predictor (e.g., size)
- Dependence on continuous environmental variables (herbivory and soil moisture) is suppressed, to simplify notation and because most vital rates are functions of one or more of these variables (**Supplementary Tables 5-6**)
- Lower-case Greek letters (i.e., γ, σ, δ) are used for size-independent vital rates pertaining to the seed bank

Definitions

- Bolting state 0 ≡ non-bolters
- Bolting state 1 ≡ bolters
- S_{min}, S_{max} ≡ minimum and maximum plant size in the IPM
- $n_{i,t}(z)$ ≡ density of plants of (continuous) size z in bolting state i at time t, $i \in (0,1)$
- $n_{i,j}$ ≡ density of plants in bolting state i and size bin j of the discretized size distribution (dependence on time t is suppressed here)
- Φ_t ≡ seed bank density in year t
- $s(z)$ ≡ survival probability for plants of size z (independent of bolting state)
- $b_i(z)$ ≡ probability that a plant of size z currently in bolting state i will bolt next year, $i \in (0,1)$
- $g_{ji}(z'|z)$ ≡ probability that a plant currently of size z in bolting state i that transitions to bolting state j will be of size z' next year; $i, j \in (0,1)$
- γ ≡ probability a seed in the soil survives winter and germinates in spring
- σ ≡ probability a germinant survives its first summer
- $c(z)$ ≡ probability a germinant that survives its first summer is size z
- $r(z)$ ≡ probability a bolting individual of size z produces any seeds
- $f(z)$ ≡ number of seeds produced by a bolting individual of size z conditional on producing any seeds
- δ ≡ one-year survival probability of seeds in the seed bank

Recursion equations

The lineage growth rate for each genotype arises from the following three components.

Component 1: Non-bolters at time $t + 1$

Non-bolters of size z' at time $t + 1$ contributed by survival of aboveground individuals existing at time t:

$$n'_{0,t+1}(z') = \int_{z=S_{min}}^{z=S_{max}} \big[g_{00}(z'|z)\ (1 - b_0(z))\ s(z)\ n_{0,t}(z) + g_{01}(z'|z)\ (1 - b_1(z))\ s(z)\ n_{1,t}(z)\big] dz$$

(*Eq. S1*)

The first term in the integral reflects non-bolters that remain non-bolters, and the second term reflects bolters that become non-bolters.

Component 2: Bolters at time $t + 1$

Bolters of size z' at time $t + 1$ contributed by survival of above-ground individuals existing at time t:

$$n_{1,t+1}(z') = \int_{z=S_{min}}^{z=S_{max}} \big[g_{10}(z'|z)\ b_0(z)\ s(z)\ n_{0,t}(z) + g_{11}(z'|z)\ b_1(z)\ s(z)\ n_{1,t}(z)\big] dz$$

(Eq. S2)

Here, the first term in the integral reflects bolters that were previously non-bolters, and the second term reflects bolters that were previously bolters.

Component 3: New non-bolters from recruitment

New non-bolters of size z' at time $t + 1$ contributed by reproduction of individuals existing at time t and by germination from the seed bank:

$$n''_{0,t+1}(z') = \int_{z=S_{min}}^{z=S_{max}} c(z')\ \sigma\ \gamma\ f(z)\ r(z)\ n_{1,t}(z) dz\ +\ c(z')\ \sigma\ \gamma\ \Phi_t$$

(Eq. S3)

Here, the first term in the integral represents the recruitment of non-bolters from seeds dispersed in the preceding year, and the second term represents the recruitment of non-bolters from seeds in the seed bank. Note that new recruits do not bolt in their first year, so there is no immediate contribution of recruitment to bolters.

Combining components

The total non-bolters of size z' at time $t + 1$ is thus defined as

$$n_{0,t+1}(z') = n'_{0,t+1}(z') + n''_{0,t+1}(z')$$

(Eq. S4)

where the first component of the sum is the number of non-bolters arising through survival of existing plants, and the second component is non-bolters arising from recruitment.

The size of the seedbank at time $t + 1$ is defined as

$$\Phi_{t+1} = \delta\,(1 - \gamma) \int_{z=S_{min}}^{z=S_{max}} f(z)\, r(z)\, n_{1,t}(z)\, dz$$

(Eq. S5)

reflecting efflux from the seed bank through mortality and germination, and influx to the seed bank via reproduction. As mentioned above, we are assuming that seeds can spend at most a single year in the seed bank, following Anderson and Wadgymar (2020).

Numerical integration

Our aim is to construct a discrete matrix that numerically approximates the IPM integrals and represents the dynamics of both the density of seeds in the seed bank and the density of above-ground plants in each size bin and bolting state. The matrix discretizes the continuous size range into nmp mesh points with a bin width $h = (S_{max} - S_{min})/nmp$ and bin midpoints $z_i = S_{min} + h \times \left(i - \frac{1}{2}\right)$ for $i = 1\ to\ nmp$. The population vector at time t corresponding to the matrix takes the form:

$$\boldsymbol{n}_t = \begin{bmatrix} \Phi \\ n_{0,1} \\ \vdots \\ n_{0,nmp} \\ n_{1,1} \\ \vdots \\ n_{1,nmp} \end{bmatrix}_t$$

where Φ is the seed bank density and $n_{i,j}$ is the density of plants in bolting state *i* and size bin *j*, with non-bolters $(i = 0)$ stacked above bolters $(i = 1)$. Corresponding to this vector, the matrix we will construct has vital rates pertaining to the seed bank in the first row and column, and the remainder of the matrix (which governs above-ground transitions and direct reproduction not involving the seed bank) is divided into four quadrants corresponding to the bolting states in successive years.

Construction and analysis of the matrix follows these steps:

1. Fill the rows of a nmp x nmp matrix with identical vectors containing the values of the (bolting-state independent) survival function $s(z)$ evaluated at the bin midpoints $z_1 \dots z_{nmp}$, and then copy the matrix into the four quadrants of the $2nmp$ x $2nmp$ survival megamatrix $\mathbf{S}$:

$$\mathbf{S} = \begin{bmatrix} \begin{bmatrix} s(z_1) & \dots & s(z_{nmp}) \\ \vdots & \ddots & \vdots \\ s(z_1) & \dots & s(z_{nmp}) \end{bmatrix} & \begin{bmatrix} s(z_1) & \dots & s(z_{nmp}) \\ \vdots & \ddots & \vdots \\ s(z_1) & \dots & s(z_{nmp}) \end{bmatrix} \\ \begin{bmatrix} s(z_1) & \dots & s(z_{nmp}) \\ \vdots & \ddots & \vdots \\ s(z_1) & \dots & s(z_{nmp}) \end{bmatrix} & \begin{bmatrix} s(z_1) & \dots & s(z_{nmp}) \\ \vdots & \ddots & \vdots \\ s(z_1) & \dots & s(z_{nmp}) \end{bmatrix} \end{bmatrix}$$

2. Construct the following two nmp x nmp matrices using the bolting probability functions $b_0(z)$ and $b_1(z)$ evaluated at the bin midpoints

$$\mathbf{B_0} = \begin{bmatrix} b_0(z_1) & \cdots & b_0(z_{nmp}) \\ \vdots & \ddots & \vdots \\ b_0(z_1) & \cdots & b_0(z_{nmp}) \end{bmatrix} \quad \text{and} \quad \mathbf{B_1} = \begin{bmatrix} b_1(z_1) & \cdots & b_1(z_{nmp}) \\ \vdots & \ddots & \vdots \\ b_1(z_1) & \cdots & b_1(z_{nmp}) \end{bmatrix}$$

and then use them to make the $2nmp$ x $2nmp$ bolting megamatrix $\mathbf{B}$ as follows

$$\mathbf{B} = \begin{bmatrix} 1 - \mathbf{B_0} & 1 - \mathbf{B_1} \\ \mathbf{B_0} & \mathbf{B_1} \end{bmatrix}$$

3. Use the growth functions $g_{ji}(z'|z)$ to construct the $2nmp$ x $2nmp$ growth megamatrix $\mathbf{G}$ as follows:

$$\mathbf{G} = h \begin{bmatrix} \begin{bmatrix} g_{00}(z_1|z_1) & \cdots & g_{00}(z_1|z_{nmp}) \\ \vdots & \ddots & \vdots \\ g_{00}(z_{nmp}|z_1) & \cdots & g_{00}(z_{nmp}|z_{nmp}) \end{bmatrix} & \begin{bmatrix} g_{01}(z_1|z_1) & \cdots & g_{01}(z_1|z_{nmp}) \\ \vdots & \ddots & \vdots \\ g_{01}(z_{nmp}|z_1) & \cdots & g_{01}(z_{nmp}|z_{nmp}) \end{bmatrix} \\ \begin{bmatrix} g_{10}(z_1|z_1) & \cdots & g_{10}(z_1|z_{nmp}) \\ \vdots & \ddots & \vdots \\ g_{10}(z_{nmp}|z_1) & \cdots & g_{10}(z_{nmp}|z_{nmp}) \end{bmatrix} & \begin{bmatrix} g_{11}(z_1|z_1) & \cdots & g_{11}(z_1|z_{nmp}) \\ \vdots & \ddots & \vdots \\ g_{11}(z_{nmp}|z_1) & \cdots & g_{11}(z_{nmp}|z_{nmp}) \end{bmatrix} \end{bmatrix}$$

4. where h is the bin width. Note that the upper left quadrant of $\mathbf{G}$ describes growth of plants that did not bolt either this year or last, lower left is plants that bolted this year but not last year, etc. (Note: each of the submatrices of $\mathbf{G}$ should be assessed for eviction by identifying columns that do not sum to one, and then renormalizing those columns. This is the procedure followed in the R code that accompanies this paper. For simplicity, we assume below that the $\mathbf{G}$ matrix has been corrected for eviction.) Construct the $2nmp$ x $2nmp$ megamatrix $\mathbf{P}$ that governs size transitions of existing above-ground plants in the discretized IPM,
$$\mathbf{P} = \mathbf{S} \circ \mathbf{B} \circ \mathbf{G}$$
where ∘ denotes the Hadamard product (i.e., element-wise multiplication).

5. Use the vital rate functions describing reproduction to construct the nmp x nmp matrix $\mathbf{F}'$,

$$\mathbf{F}' = \sigma\gamma \begin{bmatrix} c(z_1)\, f(z_1)\, r(z_1) & \cdots & c(z_1)\, f(z_{nmp})\, r(z_{nmp}) \\ \vdots & \ddots & \vdots \\ c(z_{nmp})\, f(z_1)\, r(z_1) & \cdots & c(z_{nmp})\, f(z_{nmp})\, r(z_{nmp}) \end{bmatrix}$$

and then use $\mathbf{F}'$ to construct the $2nmp$ x $2nmp$ megamatrix $\mathbf{F}$ that governs production of new plants through the production by existing plants of seeds that germinate in their first year:

$$\mathbf{F} = h \begin{bmatrix} \mathbf{0} & \mathbf{F'} \\ \mathbf{0} & \mathbf{0} \end{bmatrix}$$

where $\mathbf{0}$ denotes a nmp x nmp matrix of zeros. h is again the bin width.

6. Construct the vector $\boldsymbol{p}$ describing new above-ground plants coming from seeds in the seed bank, which by definition are non-bolters (following the second term on the right-hand side of Eq. S3):

$$\boldsymbol{p} = \sigma\,\gamma \begin{bmatrix} c(z_1) \\ \vdots \\ c(z_{nmp}) \\ 0 \\ \vdots \\ 0 \end{bmatrix}$$

7. Construct the vector that describes the contributions of plants in each size bin to next year's seed bank (following Eq. S5):

$$\boldsymbol{q} = \delta(1 - \gamma) \begin{bmatrix} 0 \\ \vdots \\ 0 \\ f(z_1)\, r(z_1) \\ \vdots \\ f(z_{nmp})\, r(z_{nmp}) \end{bmatrix}$$

8. Assemble the matrices and vectors described above into the desired matrix $\mathbf{A}$ describing the above- and below-ground dynamics:

$$\mathbf{A} = \begin{bmatrix} 0 & \boldsymbol{q}^{\mathrm{T}} \\ \boldsymbol{p} & \mathbf{P} + \mathbf{F} \end{bmatrix}$$

   where $\boldsymbol{q}^{\mathrm{T}}$ is the row vector representing the transpose of the column vector $\boldsymbol{q}$. The asymptotic lineage growth rate is the dominant eigenvalue of $\mathbf{A}$.

   (Note: if we had assumed that seeds could spend more than one year in the seed bank, the upper left element of $\mathbf{A}$ would not have been zero.)

Bootstrap simulations

In each of 1,000 bootstrap runs, we parameterized size- and/or driver-dependent vital rate regressions by independently sampling coefficient estimates from the multivariate Normal distribution defined by the best estimates of the regression coefficients and the variance-covariance matrix of fixed effects in each regression (Wilbur et al. 2016, Visser et al. 2018, Carley et al. 2022). For size- and driver-independent vital rates, details on how we accounted for parameter uncertainty are as follows.

We sampled dormancy rates from a Normal distribution defined by the empirical mean of four estimates reported in Anderson & Wadgymar 2020 (**Supplementary Table 5**) and a standard deviation equal to the standard error of the empirical mean. Since the Normal distribution is not bounded, occasionally it is possible to draw dormancy rates outside of the 0-1 range. To account for this, we filtered any outcomes during bootstrapping such that rates estimated as <0 or >1 were corrected to 0 and 1, respectively. (Given the empirical mean and standard error, this occurs very rarely, in less than 0.001% of sampling events.)

We sampled germination from a binomial distribution defined by the number of successes (138 germinants) and the number of trials (1220 seeds planted) reported in Anderson et al. (2020b). We divided the sampled number of successful germinants by the number of trials to calculate a sampled germination proportion.

We sampled germinant survival as described for germination, here with the number of successes as 89 surviving germinants and the number of trials as 138 initial germinants (Anderson et al. 2020b).

We also sampled the parameters used to define the genotype-specific Normal distributions of new recruit size. We sampled the mean recruit size for each genotype by drawing from a Normal distribution defined by the empirical mean and variance of 400 juvenile plants per genotype (**Supplementary Table 5**). We sampled the mean of the Normal recruit size distribution using the empirical mean and its standard error, as described above. We sampled the standard deviation of the Normal recruit size distribution by multiplying the empirical variance by a sampled value from a chi-square distribution parameterized by the sample size minus one (here, 399 for each genotype). This approach follows Dennis et al. (1991) and Carley et al. (2022).

Driver and vital rate contributions to λ

We approximated the proportional contributions of each driver to λ, and which vital rates these contributions are mediated through. To do so, we first computed λ_{med} for each genotype at median driver values using the best-fit vital rate coefficients following model selection. We then calculated λ^*_{med} using the same parameters, except setting the coefficients linking one driver to one vital rate function (both through main effects and interactive effects) to 0. We calculated λ^*_{med} for all combinations of the 13 driver-sensitive vital rates and 2 drivers for each genotype. We then calculated the proportional contribution of each driver to λ, mediated by each vital rate, as follows:

$$c = \frac{\lambda_{med} - \lambda^*_{med}}{\lambda_{med}}$$

Positive values of *c* indicate that “removing” the effect of a driver on a vital rate decreased λ; in other words, inclusion of that driver’s effect on that vital rate increases λ (when the median values of both drivers are allowed to affect every other vital rate in the IPM). Conversely, negative values indicate that inclusion of that driver’s effect on that vital rate decreases λ.

Appendix S3: Outcomes of demographic model selection

Tables S1-S18 in this Appendix report the outcomes of model selection for each genotype- and state-specific vital rate included in our demographic model. Specifically, the coefficient estimates of each maximal model (including effects of herbivory, soil moisture, size, and all 2- and 3-way interactions on the vital rate) and nested reduced models are presented, in order from the lowest to highest AICc values. We compared AICc to perform model selection and choose which drivers to retain in the vital rate functions that parameterized each genotype's IPM (**Methods; Appendix S2**). For each table, the genotype and the vital rate that is the response variable in a given set of models are named in the table legend. Vital rates named in the table legends match those defined in **Supplementary Table 5.**

For all tables S1-S18, column names are as follows:

Int: coefficient estimate for the intercept

h: coefficient estimate for effect of herbivory on the vital rate

w: coefficient estimate for the effect of soil moisture on the vital rate

z: coefficient estimate for the effect of plant size on the vital rate

h×w: coefficient estimate for the effect of the herbivory-soil moisture interaction on the vital rate

h×z: coefficient estimate for the effect of the herbivory-size interaction on the vital rate

w×z: coefficient estimate for the effect of the soil moisture-size interaction on the vital rate

w×h×z: coefficient estimate for the effect of the three-way soil moisture, herbivory, and plant size interaction on the vital rate

df: degrees of freedom in the model in a given row

logLik: the log likelihood of the model in a given row

AICc: the AICc value of the model in a given row

delta: the change in AICc between the model in a given row and the model in the row above it (i.e. the model with the next-lowest AICc value)

Table S1. Model selection comparisons: *S(z, h, w)*, *BB* genotype.

Int.	*h*	*w*	*z*	*h*×*w*	*h*×*z*	*w*×*z*	*w*×*h*×*z*	df	logLik	AICc	delta
-1.498	NA	20.589	0.017	NA	NA	NA	NA	3.000	-1099.687	2205.387	0.000
-1.465	-0.009	20.513	0.018	NA	NA	NA	NA	4.000	-1099.077	2206.175	0.788
-1.620	NA	21.752	0.030	NA	NA	-0.117	NA	4.000	-1099.396	2206.813	1.426
-1.580	-0.009	21.610	0.030	NA	NA	-0.111	NA	5.000	-1098.820	2207.671	2.284
-1.527	0.006	21.054	0.018	-0.134	NA	NA	NA	5.000	-1098.939	2207.909	2.522
-1.449	-0.013	20.460	0.017	NA	0.000	NA	NA	5.000	-1098.996	2208.022	2.635
-1.627	0.005	22.015	0.030	-0.118	NA	-0.104	NA	6.000	-1098.716	2209.475	4.088
-1.564	-0.013	21.553	0.029	NA	0.000	-0.110	NA	6.000	-1098.741	2209.524	4.137
-1.510	0.002	20.996	0.017	-0.132	0.000	NA	NA	6.000	-1098.860	2209.764	4.377
-1.611	0.001	21.965	0.028	-0.118	0.000	-0.104	NA	7.000	-1098.636	2211.329	5.942
-1.586	-0.007	21.733	0.025	-0.044	0.001	-0.074	-0.007	8.000	-1098.595	2213.263	7.876
-1.428	NA	21.655	NA	NA	NA	NA	NA	2.000	-1108.248	2220.502	15.115
-1.414	-0.004	21.643	NA	NA	NA	NA	NA	3.000	-1108.152	2222.316	16.929
-1.475	0.012	22.183	NA	-0.136	NA	NA	NA	4.000	-1108.019	2224.058	18.672
0.663	-0.011	NA	0.025	NA	NA	NA	NA	3.000	-1164.825	2335.662	130.275
0.633	NA	NA	0.024	NA	NA	NA	NA	2.000	-1165.880	2335.766	130.379
0.692	-0.021	NA	0.022	NA	0.001	NA	NA	4.000	-1164.164	2336.349	130.962
0.888	NA	NA	NA	NA	NA	NA	NA	1.000	-1184.114	2370.231	164.844
0.901	-0.004	NA	NA	NA	NA	NA	NA	2.000	-1183.997	2372.000	166.613

Table S2. Model selection comparisons: *S(z, h, w)*, *MM* genotype.

Int.	*h*	*w*	*z*	*h*×*w*	*h*×*z*	*w*×*z*	*w*×*h*×*z*	df	logLik	AICc	delta
-1.327	-0.001	21.392	-0.004	-0.535	0.005	NA	NA	6.000	-1202.100	2416.239	0.000
-1.176	-0.053	19.954	-0.003	NA	0.005	NA	NA	5.000	-1203.238	2416.504	0.265
-1.398	-0.051	22.080	0.015	NA	0.005	-0.172	NA	6.000	-1202.558	2417.154	0.915
-1.509	-0.003	23.144	0.012	-0.499	0.005	-0.150	NA	7.000	-1201.593	2417.238	0.999
-1.597	0.038	23.985	0.023	-0.916	0.001	-0.250	0.038	8.000	-1201.013	2418.092	1.853
-1.395	NA	20.805	0.013	NA	NA	NA	NA	3.000	-1217.433	2440.876	24.637
-1.641	NA	23.207	0.034	NA	NA	-0.196	NA	4.000	-1216.555	2441.128	24.889
-1.404	0.003	20.845	0.012	NA	NA	NA	NA	4.000	-1217.388	2442.795	26.556
-1.660	0.004	23.317	0.034	NA	NA	-0.201	NA	5.000	-1216.474	2442.976	26.737
-1.436	0.013	21.137	0.013	-0.098	NA	NA	NA	5.000	-1217.343	2444.713	28.473
-1.662	0.005	23.334	0.034	-0.009	NA	-0.200	NA	6.000	-1216.474	2444.986	28.747
-1.308	NA	21.468	NA	NA	NA	NA	NA	2.000	-1222.134	2448.273	32.034
-1.341	0.008	21.539	NA	NA	NA	NA	NA	3.000	-1221.723	2449.457	33.218
-1.337	0.007	21.501	NA	0.013	NA	NA	NA	4.000	-1221.722	2451.463	35.224
0.878	-0.076	NA	0.000	NA	0.006	NA	NA	4.000	-1269.509	2547.036	130.797
0.698	NA	NA	0.019	NA	NA	NA	NA	2.000	-1291.444	2586.894	170.655
0.708	-0.005	NA	0.019	NA	NA	NA	NA	3.000	-1291.260	2588.531	172.292
0.929	NA	NA	NA	NA	NA	NA	NA	1.000	-1303.010	2608.022	191.783
0.918	0.004	NA	NA	NA	NA	NA	NA	2.000	-1302.913	2609.832	193.593

Table S3. Model selection comparisons: *B0(z, h, w)*, *BB* genotype.

Int.	*h*	*w*	*z*	*h*×*w*	*h*×*z*	*w*×*z*	*w*×*h*×*z*	df	logLik	AICc	delta
0.957	NA	-50.613	-2.429	NA	NA	33.062	NA	4.000	-89.538	187.182	0.000
0.960	-0.006	-50.586	-2.411	NA	NA	32.956	NA	5.000	-89.522	189.204	2.022
-4.179	NA	NA	0.982	NA	NA	NA	NA	2.000	-93.270	190.571	3.389
-5.226	NA	11.851	0.907	NA	NA	NA	NA	3.000	-92.287	190.638	3.457
1.103	-0.044	-50.984	-2.494	NA	0.019	33.230	NA	6.000	-89.453	191.131	3.949
0.972	-0.025	-50.727	-2.392	0.218	NA	32.744	NA	6.000	-89.515	191.254	4.073
-4.166	-0.012	NA	0.996	NA	NA	NA	NA	3.000	-93.182	192.427	5.245
-5.193	-0.007	11.577	0.917	NA	NA	NA	NA	4.000	-92.255	192.616	5.434
1.130	-0.088	-51.073	-2.463	0.422	0.023	32.744	NA	7.000	-89.426	193.152	5.970
-4.078	-0.049	NA	0.954	NA	0.017	NA	NA	4.000	-93.132	194.370	7.188
-5.007	-0.074	9.681	0.907	0.762	NA	NA	NA	5.000	-92.165	194.490	7.309
-5.113	-0.037	11.527	0.880	NA	0.014	NA	NA	5.000	-92.216	194.592	7.411
0.911	0.017	-48.565	-2.362	-0.774	-0.024	31.554	0.545	8.000	-89.415	195.217	8.035
-4.188	NA	16.254	NA	NA	NA	NA	NA	2.000	-95.769	195.570	8.389
-4.772	-0.158	8.801	0.822	1.085	0.028	NA	NA	6.000	-92.044	196.312	9.130
-2.573	NA	NA	NA	NA	NA	NA	NA	1.000	-97.488	196.986	9.805
-4.194	0.001	16.282	NA	NA	NA	NA	NA	3.000	-95.769	197.601	10.420
-2.561	-0.004	NA	NA	NA	NA	NA	NA	2.000	-97.477	198.987	11.805
-3.921	-0.108	13.376	NA	1.191	NA	NA	NA	4.000	-95.601	199.307	12.126

Table S4. Model selection comparisons: *B0(z, h, w)*, *MM* genotype.

Int.	*h*	*w*	*z*	*h*×*w*	*h*×*z*	*w*×*z*	*w*×*h*×*z*	df	logLik	AICc	delta
-6.308	NA	NA	1.894	NA	NA	NA	NA	2.000	-61.442	126.917	0.000
-6.430	0.020	NA	1.913	NA	NA	NA	NA	3.000	-61.173	128.412	1.495
-5.722	NA	-6.642	1.914	NA	NA	NA	NA	3.000	-61.240	128.545	1.628
-5.880	0.019	-6.252	1.934	NA	NA	NA	NA	4.000	-60.992	130.096	3.178
-3.943	NA	-26.674	1.146	NA	NA	8.608	NA	4.000	-61.057	130.225	3.307
-6.357	0.002	NA	1.873	NA	0.010	NA	NA	4.000	-61.153	130.416	3.499
-4.278	0.018	-24.185	1.245	NA	NA	7.700	NA	5.000	-60.847	131.861	4.943
-6.109	0.054	-3.951	1.947	-0.435	NA	NA	NA	5.000	-60.900	131.967	5.050
-5.754	-0.006	-6.566	1.880	NA	0.014	NA	NA	5.000	-60.956	132.079	5.161
-5.997	0.019	-2.489	1.811	-0.869	0.041	NA	NA	6.000	-60.705	133.644	6.727
-4.564	0.050	-21.142	1.291	-0.404	NA	7.307	NA	6.000	-60.771	133.775	6.858
-3.993	-0.014	-26.041	1.123	NA	0.018	8.325	NA	6.000	-60.789	133.813	6.895
-4.132	0.009	-22.793	0.999	-0.926	0.048	8.786	NA	7.000	-60.522	135.357	8.440
-3.690	-0.095	-27.995	0.754	0.397	0.103	11.605	-0.675	8.000	-60.497	137.396	10.479
-2.905	NA	NA	NA	NA	NA	NA	NA	1.000	-74.704	151.419	24.501
-2.956	0.012	NA	NA	NA	NA	NA	NA	2.000	-74.572	153.178	26.261
-2.402	NA	-5.427	NA	NA	NA	NA	NA	2.000	-74.579	153.191	26.274
-2.510	0.011	-4.747	NA	NA	NA	NA	NA	3.000	-74.478	155.023	28.105
-2.508	0.010	-4.765	NA	0.004	NA	NA	NA	4.000	-74.478	157.067	30.150

Table S5. Model selection comparisons: *B1(z, h, w)*, *BB* genotype.

Int.	*h*	*w*	*z*	*h*×*w*	*h*×*z*	*w*×*z*	*w*×*h*×*z*	df	logLik	AICc	delta
-5.960	0.037	28.412	0.146	NA	NA	-0.535	NA	5.000	-305.931	621.967	0.000
-5.603	NA	26.377	0.140	NA	NA	-0.487	NA	4.000	-307.697	623.463	1.496
-4.224	0.033	14.144	0.081	NA	NA	NA	NA	4.000	-307.736	623.542	1.575
-5.843	-0.003	27.377	0.145	0.403	NA	-0.533	NA	6.000	-305.834	623.815	1.847
-5.987	0.044	28.464	0.147	NA	0.000	-0.537	NA	6.000	-305.905	623.957	1.990
-4.048	NA	13.486	0.080	NA	NA	NA	NA	3.000	-309.217	624.476	2.509
-4.110	-0.009	13.112	0.080	0.436	NA	NA	NA	5.000	-307.629	625.362	3.395
-4.240	0.039	14.140	0.082	NA	0.000	NA	NA	5.000	-307.720	625.544	3.577
-5.845	-0.006	26.969	0.148	0.609	-0.001	-0.537	NA	7.000	-305.724	625.644	3.677
-5.356	-0.209	22.260	0.126	2.594	0.008	-0.340	-0.081	8.000	-305.222	626.697	4.730
-4.101	-0.012	12.659	0.083	0.616	-0.001	NA	NA	6.000	-307.542	627.231	5.264
-2.605	NA	NA	0.086	NA	NA	NA	NA	2.000	-317.080	638.181	16.214
-2.682	0.024	NA	0.086	NA	NA	NA	NA	3.000	-316.239	638.519	16.552
-2.701	0.031	NA	0.087	NA	0.000	NA	NA	4.000	-316.216	640.502	18.535
-2.411	-0.096	16.357	NA	1.305	NA	NA	NA	4.000	-368.840	745.749	123.782
-2.734	0.035	19.453	NA	NA	NA	NA	NA	3.000	-369.966	745.974	124.007
-2.573	NA	18.880	NA	NA	NA	NA	NA	2.000	-371.662	747.346	125.378
-0.352	NA	NA	NA	NA	NA	NA	NA	1.000	-392.478	786.963	164.995
-0.411	0.022	NA	NA	NA	NA	NA	NA	2.000	-391.736	787.493	165.525

Table S6. Model selection comparisons: *B1(z, h, w)*, *MM* genotype.

Int.	*h*	*w*	*z*	*h×w*	*h×z*	*w×z*	*w×h×z*	df	logLik	AICc	delta
-5.621	0.041	25.929	0.175	NA	NA	-0.635	NA	5.000	-405.312	820.705	0.000
-5.432	-0.021	25.461	0.167	NA	0.003	-0.623	NA	6.000	-404.614	821.341	0.636
-5.411	NA	24.937	0.170	NA	NA	-0.597	NA	4.000	-406.797	821.647	0.942
-5.623	0.042	25.943	0.175	-0.005	NA	-0.635	NA	6.000	-405.312	822.737	2.032
-5.628	0.043	27.546	0.168	-0.800	0.004	-0.644	NA	7.000	-404.307	822.765	2.060
-3.840	0.036	10.240	0.102	NA	NA	NA	NA	4.000	-407.594	823.242	2.537
-3.746	NA	10.184	0.101	NA	NA	NA	NA	3.000	-408.815	823.663	2.958
-3.686	-0.025	10.057	0.095	NA	0.003	NA	NA	5.000	-406.800	823.681	2.976
-5.538	-0.002	26.653	0.164	-0.357	0.006	-0.604	-0.020	8.000	-404.281	824.757	4.052
-3.798	0.018	9.863	0.101	0.176	NA	NA	NA	5.000	-407.568	825.216	4.511
-3.787	0.023	11.264	0.094	-0.628	0.004	NA	NA	6.000	-406.616	825.345	4.639
-2.744	-0.035	NA	0.102	NA	0.004	NA	NA	4.000	-412.814	833.682	12.977
-2.898	0.034	NA	0.110	NA	NA	NA	NA	3.000	-413.845	833.722	13.017
-2.812	NA	NA	0.109	NA	NA	NA	NA	2.000	-415.010	834.035	13.330
-2.261	NA	19.062	NA	NA	NA	NA	NA	2.000	-488.189	980.394	159.689
-2.331	0.027	19.158	NA	NA	NA	NA	NA	3.000	-487.215	980.462	159.757
-2.160	-0.051	17.473	NA	0.780	NA	NA	NA	4.000	-486.585	981.223	160.518
-0.134	NA	NA	NA	NA	NA	NA	NA	1.000	-518.192	1038.390	217.685
-0.185	0.023	NA	NA	NA	NA	NA	NA	2.000	-517.425	1038.865	218.160

Table S7. Model selection comparisons: *G00(z', z, h, w)*, *BB* genotype.

Int.	*h*	*w*	*z*	*h×w*	*h×z*	*w×z*	*w×h×z*	df	logLik	AICc	delta
1.007	-0.051	-2.088	0.383	0.466	NA	NA	NA	6.000	-285.167	582.567	0.000
1.056	-0.060	-2.180	0.358	0.474	0.004	NA	NA	7.000	-284.643	583.598	1.031
0.768	-0.057	0.149	0.539	0.536	NA	-1.440	NA	7.000	-284.787	583.886	1.319
0.743	-0.070	0.852	0.564	0.571	0.005	-1.969	NA	8.000	-283.973	584.348	1.781
0.789	-0.011	NA	0.402	NA	NA	NA	NA	4.000	-288.624	585.358	2.792
0.649	-0.040	1.812	0.608	0.267	-0.006	-2.421	0.109	9.000	-283.844	586.191	3.625
0.823	-0.018	NA	0.380	NA	0.004	NA	NA	5.000	-288.240	586.646	4.080
0.843	-0.011	-0.637	0.406	NA	NA	NA	NA	5.000	-288.494	587.154	4.587
0.884	-0.018	-0.697	0.384	NA	0.004	NA	NA	6.000	-288.084	588.402	5.835
0.916	-0.011	-1.309	0.363	NA	NA	0.394	NA	6.000	-288.460	589.154	6.587
0.905	-0.018	-0.895	0.372	NA	0.004	0.117	NA	7.000	-288.081	590.475	7.908
0.782	NA	NA	0.383	NA	NA	NA	NA	3.000	-293.335	592.736	10.169
0.810	NA	-0.336	0.385	NA	NA	NA	NA	4.000	-293.299	594.709	12.142
0.854	NA	-0.744	0.359	NA	NA	0.240	NA	5.000	-293.287	596.741	14.174
1.453	-0.076	-0.796	NA	0.813	NA	NA	NA	5.000	-322.715	655.597	73.030
1.177	NA	2.079	NA	NA	NA	NA	NA	3.000	-332.161	670.388	87.821
1.202	-0.005	1.997	NA	NA	NA	NA	NA	4.000	-331.233	670.576	88.009
1.377	NA	NA	NA	NA	NA	NA	NA	2.000	-333.308	670.650	88.083
1.396	-0.006	NA	NA	NA	NA	NA	NA	3.000	-332.296	670.658	88.091

Table S8. Model selection comparisons: *G00(z', z, h, w)*, *MM* genotype.

Int.	*h*	*w*	*z*	*h*×*w*	*h*×*z*	*w*×*z*	*w*×*h*×*z*	df	logLik	AICc	delta
0.972	-0.008	-2.208	0.425	NA	NA	NA	NA	5.000	-292.086	594.343	0.000
0.787	-0.008	NA	0.408	NA	NA	NA	NA	4.000	-293.435	594.984	0.641
1.020	-0.014	-2.343	0.401	NA	0.003	NA	NA	6.000	-291.543	595.326	0.983
0.818	-0.012	NA	0.387	NA	0.003	NA	NA	5.000	-293.052	596.275	1.932
0.884	-0.008	-1.392	0.479	NA	NA	-0.483	NA	6.000	-292.040	596.320	1.977
0.984	-0.011	-2.308	0.423	0.024	NA	NA	NA	6.000	-292.069	596.377	2.034
0.995	-0.008	-1.995	0.398	-0.098	0.005	NA	NA	7.000	-291.364	597.049	2.706
0.886	-0.014	-1.082	0.483	NA	0.004	-0.753	NA	7.000	-291.433	597.187	2.844
0.892	-0.011	-1.463	0.479	0.027	NA	-0.508	NA	7.000	-292.018	598.357	4.014
0.770	NA	NA	0.398	NA	NA	NA	NA	3.000	-296.410	598.888	4.545
0.855	-0.008	-0.678	0.483	-0.100	0.005	-0.782	NA	8.000	-291.246	598.905	4.562
0.926	NA	-1.872	0.413	NA	NA	NA	NA	4.000	-295.448	599.009	4.666
0.716	0.015	0.699	0.584	-0.310	-0.013	-1.743	0.159	9.000	-290.723	599.965	5.622
0.732	NA	-0.072	0.533	NA	NA	-1.075	NA	5.000	-295.218	600.608	6.265
1.436	-0.030	-0.401	NA	0.289	NA	NA	NA	5.000	-332.232	674.634	80.291
1.405	-0.005	NA	NA	NA	NA	NA	NA	3.000	-334.568	675.205	80.862
1.385	NA	NA	NA	NA	NA	NA	NA	2.000	-335.591	675.215	80.872
1.279	NA	1.114	NA	NA	NA	NA	NA	3.000	-335.302	676.671	82.328
1.313	-0.005	0.968	NA	NA	NA	NA	NA	4.000	-334.350	676.814	82.471

Table S9. Model selection comparisons: *G01(z', z, h, w)*, *BB* genotype.

Int.	*h*	*w*	*z*	*h×w*	*h×z*	*w×z*	*w×h×z*	df	logLik	AICc	delta
-14.492	-0.534	169.528	10.750	NA	NA	-35.138	NA	6.000	-1342.986	2698.212	0.000
-4.579	0.242	88.232	6.142	-5.395	NA	NA	NA	6.000	-1343.094	2698.427	0.216
-1.791	-0.152	45.645	6.931	NA	-0.095	NA	NA	6.000	-1343.181	2698.603	0.391
2.957	-0.088	NA	7.287	NA	-0.107	NA	NA	5.000	-1344.233	2698.636	0.424
-0.051	-0.529	52.320	6.263	NA	NA	NA	NA	5.000	-1344.329	2698.829	0.617
-5.490	0.467	77.860	6.732	-4.699	-0.081	NA	NA	7.000	-1342.262	2698.846	0.634
-15.064	0.062	173.518	9.747	-4.169	NA	-28.016	NA	7.000	-1342.300	2698.920	0.708
-12.799	-0.253	140.366	10.319	NA	-0.070	-27.880	NA	7.000	-1342.409	2699.139	0.927
5.773	-0.513	NA	6.573	NA	NA	NA	NA	4.000	-1345.727	2699.568	1.356
-13.470	0.280	146.463	9.419	-3.887	-0.064	-21.829	NA	8.000	-1341.814	2700.042	1.831
-16.410	1.079	168.446	10.256	-9.545	-0.267	-28.008	1.430	9.000	-1341.729	2701.977	3.765
1.409	NA	NA	6.583	NA	NA	NA	NA	3.000	-1384.229	2774.527	76.315
1.092	NA	2.779	6.566	NA	NA	NA	NA	4.000	-1384.226	2776.566	78.354
-8.547	NA	80.746	9.566	NA	NA	-23.472	NA	5.000	-1383.747	2777.664	79.452
0.457	0.935	233.699	NA	-10.490	NA	NA	NA	5.000	-1394.488	2799.146	100.934
9.625	-0.565	168.217	NA	NA	NA	NA	NA	4.000	-1398.032	2804.177	105.965
32.395	-0.515	NA	NA	NA	NA	NA	NA	3.000	-1409.710	2825.488	127.276
11.352	NA	121.155	NA	NA	NA	NA	NA	3.000	-1432.372	2870.811	172.599
28.058	NA	NA	NA	NA	NA	NA	NA	2.000	-1437.591	2879.217	181.005

Table S10. Model selection comparisons: *G01(z', z, h, w)*, *MM* genotype.

Int.	*h*	*w*	*z*	*h×w*	*h×z*	*w×z*	*w×h×z*	df	logLik	AICc	delta
7.907	0.513	NA	5.440	NA	-0.207	NA	NA	5.000	-1354.206	2718.578	0.000
4.248	0.476	38.659	5.051	NA	-0.198	NA	NA	6.000	-1353.247	2718.726	0.148
-9.812	0.482	151.792	8.856	NA	-0.200	-29.836	NA	7.000	-1352.263	2718.836	0.258
-30.715	3.426	319.920	12.981	-23.389	-0.771	-63.353	4.589	9.000	-1350.372	2719.246	0.668
0.797	1.007	71.612	4.779	-4.729	-0.168	NA	NA	7.000	-1352.550	2719.411	0.834
-13.216	1.011	184.347	8.576	-4.712	-0.170	-29.762	NA	8.000	-1351.567	2719.535	0.957
3.141	0.623	93.221	3.533	-7.204	NA	NA	NA	6.000	-1354.580	2721.391	2.814
-10.424	0.622	202.903	7.201	-7.221	NA	-28.878	NA	7.000	-1353.664	2721.638	3.061
9.647	-0.384	44.336	3.615	NA	NA	NA	NA	5.000	-1356.338	2722.842	4.264
-3.837	-0.388	153.378	7.264	NA	NA	-28.741	NA	6.000	-1355.440	2723.112	4.535
14.154	-0.386	NA	3.989	NA	NA	NA	NA	4.000	-1357.586	2723.282	4.705
8.401	0.856	163.854	NA	-8.723	NA	NA	NA	5.000	-1370.137	2750.440	31.863
16.464	-0.364	106.345	NA	NA	NA	NA	NA	4.000	-1372.515	2753.140	34.563
7.094	NA	48.179	3.406	NA	NA	NA	NA	4.000	-1373.761	2755.631	37.054
11.977	NA	NA	3.811	NA	NA	NA	NA	3.000	-1375.102	2756.269	37.692
-3.274	NA	131.877	6.204	NA	NA	-22.052	NA	5.000	-1373.279	2756.723	38.145
31.085	-0.364	NA	NA	NA	NA	NA	NA	3.000	-1380.075	2766.216	47.638
13.666	NA	106.590	NA	NA	NA	NA	NA	3.000	-1386.975	2780.015	61.438
28.318	NA	NA	NA	NA	NA	NA	NA	2.000	-1394.007	2792.047	73.470

Table S11. Model selection comparisons: *G10(z', z, h, w)*, *BB* genotype.

Int.	*h*	*w*	*z*	*h×w*	*h×z*	*w×z*	*w×h×z*	df	logLik	AICc	delta
1.085	NA	3.198	NA	NA	NA	NA	NA	3.000	-277.072	560.215	0.000
1.063	NA	3.012	0.002	NA	NA	NA	NA	4.000	-276.679	561.477	1.262
1.095	-0.002	3.157	NA	NA	NA	NA	NA	4.000	-277.020	562.159	1.944
1.187	-0.041	2.261	NA	0.397	NA	NA	NA	5.000	-276.198	562.576	2.361
0.907	NA	4.389	0.011	NA	NA	-0.075	NA	5.000	-276.277	562.733	2.518
1.074	-0.003	2.961	0.002	NA	NA	NA	NA	5.000	-276.610	563.399	3.184
1.164	-0.040	2.118	0.002	0.380	NA	NA	NA	6.000	-275.855	563.963	3.748
0.919	-0.003	4.332	0.011	NA	NA	-0.074	NA	6.000	-276.212	564.676	4.461
0.990	-0.044	3.661	0.012	0.422	NA	-0.089	NA	7.000	-275.295	564.928	4.713
1.053	0.006	2.994	0.003	NA	0.000	NA	NA	6.000	-276.456	565.163	4.948
1.147	-0.033	2.002	0.004	0.458	-0.001	NA	NA	7.000	-275.436	565.210	4.995
0.972	-0.037	3.546	0.014	0.500	-0.001	-0.089	NA	8.000	-274.875	566.185	5.970
0.902	0.005	4.337	0.012	NA	0.000	-0.073	NA	7.000	-276.073	566.484	6.269
1.364	NA	NA	0.004	NA	NA	NA	NA	3.000	-280.892	567.856	7.641
1.068	-0.079	2.701	0.009	0.877	0.001	-0.047	-0.017	9.000	-274.668	567.881	7.666
1.435	NA	NA	NA	NA	NA	NA	NA	2.000	-282.000	568.035	7.820
1.377	-0.006	NA	0.004	NA	NA	NA	NA	4.000	-280.614	569.348	9.133
1.448	-0.005	NA	NA	NA	NA	NA	NA	3.000	-281.755	569.582	9.367
1.365	0.000	NA	0.004	NA	0.000	NA	NA	5.000	-280.539	571.259	11.044

Table S12. Model selection comparisons: *G10(z', z, h, w*), MM genotype.

Int.	*h*	*w*	*z*	*h×w*	*h×z*	*w×z*	*w×h×z*	df	logLik	AICc	delta
1.317	NA	NA	0.005	NA	NA	NA	NA	3.000	-314.341	634.742	0.000
1.411	NA	NA	NA	NA	NA	NA	NA	2.000	-315.908	635.846	1.104
1.266	NA	0.596	0.004	NA	NA	NA	NA	4.000	-314.144	636.389	1.647
1.308	0.003	NA	0.005	NA	NA	NA	NA	4.000	-314.224	636.549	1.807
1.304	NA	1.030	NA	NA	NA	NA	NA	3.000	-315.264	636.588	1.846
1.333	-0.009	NA	0.003	NA	0.001	NA	NA	5.000	-313.255	636.663	1.921
1.073	NA	2.425	0.015	NA	NA	-0.095	NA	5.000	-313.656	637.465	2.723
1.406	0.002	NA	NA	NA	NA	NA	NA	3.000	-315.852	637.765	3.024
1.330	-0.029	-0.165	0.004	0.355	NA	NA	NA	6.000	-312.829	637.873	3.131
1.372	-0.030	0.252	NA	0.361	NA	NA	NA	5.000	-313.961	638.075	3.333
1.256	0.003	0.609	0.005	NA	NA	NA	NA	5.000	-314.018	638.189	3.447
1.286	-0.009	0.541	0.003	NA	0.001	NA	NA	6.000	-313.093	638.400	3.658
1.297	0.003	1.049	NA	NA	NA	NA	NA	4.000	-315.186	638.474	3.733
1.141	-0.027	1.618	0.014	0.340	NA	-0.091	NA	7.000	-312.386	639.058	4.316
1.051	0.004	2.543	0.016	NA	NA	-0.100	NA	6.000	-313.479	639.172	4.430
1.288	-0.099	0.033	0.004	1.179	0.005	0.008	-0.052	9.000	-310.454	639.370	4.628
1.101	-0.008	2.275	0.013	NA	0.001	-0.089	NA	7.000	-312.663	639.612	4.870
1.324	-0.026	0.006	0.004	0.262	0.000	NA	NA	7.000	-312.723	639.732	4.990
1.141	-0.024	1.727	0.014	0.259	0.000	-0.088	NA	8.000	-312.302	640.973	6.231

Table S13. Model selection comparisons: *G11(z', z, h, w)*, *BB* genotype.

Int.	*h*	*w*	*z*	*h×w*	*h×z*	*w×z*	*w×h×z*	df	logLik	AICc	delta
4.301	1.034	78.187	0.004	-14.488	0.026	NA	NA	7.000	-809.162	1632.809	0.000
10.910	-1.119	13.367	-0.244	7.406	0.110	2.264	-0.786	9.000	-807.491	1633.768	0.958
5.558	1.027	67.937	-0.035	-14.432	0.026	0.311	NA	8.000	-809.138	1634.902	2.092
6.982	NA	40.205	0.076	NA	NA	NA	NA	4.000	-814.376	1636.924	4.114
9.258	NA	41.983	NA	NA	NA	NA	NA	3.000	-816.200	1638.502	5.693
8.853	NA	24.878	0.018	NA	NA	0.474	NA	5.000	-814.321	1638.899	6.090
6.886	0.016	40.484	0.076	NA	NA	NA	NA	5.000	-814.357	1638.972	6.163
5.350	0.540	54.052	0.081	-5.261	NA	NA	NA	6.000	-813.406	1639.174	6.365
7.463	-0.133	40.605	0.046	NA	0.009	NA	NA	6.000	-813.485	1639.333	6.523
11.863	NA	NA	0.081	NA	NA	NA	NA	3.000	-817.210	1640.523	7.713
9.218	0.008	42.120	NA	NA	NA	NA	NA	4.000	-816.196	1640.562	7.753
8.674	0.013	25.930	0.021	NA	NA	0.448	NA	6.000	-814.308	1640.979	8.169
8.020	0.460	53.959	NA	-4.555	NA	NA	NA	5.000	-815.489	1641.235	8.425
6.654	0.532	43.427	0.042	-5.206	NA	0.323	NA	7.000	-813.381	1641.246	8.436
9.555	-0.138	23.623	-0.019	NA	0.009	0.523	NA	7.000	-813.418	1641.321	8.512
14.535	NA	NA	NA	NA	NA	NA	NA	2.000	-819.250	1642.551	9.741
11.866	-0.001	NA	0.081	NA	NA	NA	NA	4.000	-817.210	1642.592	9.782
12.452	-0.148	NA	0.051	NA	0.009	NA	NA	5.000	-816.376	1643.009	10.200
14.565	-0.010	NA	NA	NA	NA	NA	NA	3.000	-819.242	1644.586	11.777

Table S14. Model selection comparisons: *G11(z', z, h, w)*, *MM* genotype.

Int.	*h*	*w*	*z*	*h×w*	*h×z*	*w×z*	*w×h×z*	df	logLik	AICc	delta
16.447	-1.272	-4.625	NA	10.748	NA	NA	NA	5.000	-1182.843	2375.860	0.000
16.016	-4.397	0.927	0.012	35.281	0.114	-0.143	-0.887	9.000	-1179.337	2377.203	1.343
9.740	-1.252	50.279	0.235	10.626	NA	-1.897	NA	7.000	-1181.566	2377.459	1.599
15.947	NA	NA	NA	NA	NA	NA	NA	2.000	-1186.728	2377.490	1.630
13.752	NA	18.221	NA	NA	NA	NA	NA	3.000	-1185.713	2377.496	1.636
16.203	-1.264	-5.106	0.010	10.680	NA	NA	NA	6.000	-1182.807	2377.858	1.998
10.254	-1.512	52.791	0.214	9.805	0.011	-1.940	NA	8.000	-1181.149	2378.721	2.861
6.948	NA	72.679	0.241	NA	NA	-1.902	NA	5.000	-1184.401	2378.977	3.117
15.210	NA	NA	0.025	NA	NA	NA	NA	3.000	-1186.512	2379.094	3.234
16.818	-1.506	-3.939	-0.014	9.917	0.010	NA	NA	7.000	-1182.448	2379.223	3.364
13.402	NA	17.267	0.015	NA	NA	NA	NA	4.000	-1185.630	2379.376	3.516
15.851	0.040	NA	NA	NA	NA	NA	NA	3.000	-1186.655	2379.379	3.519
13.658	0.040	18.213	NA	NA	NA	NA	NA	4.000	-1185.641	2379.397	3.538
6.799	0.044	73.184	0.242	NA	NA	-1.919	NA	6.000	-1184.313	2380.870	5.011
15.131	0.038	NA	0.024	NA	NA	NA	NA	4.000	-1186.446	2381.009	5.149
7.879	-0.480	74.264	0.211	NA	0.016	-1.979	NA	7.000	-1183.407	2381.142	5.282
13.321	0.039	17.286	0.015	NA	NA	NA	NA	5.000	-1185.562	2381.299	5.439
16.335	-0.482	NA	-0.014	NA	0.016	NA	NA	5.000	-1185.563	2381.301	5.441
14.547	-0.462	16.638	-0.021	NA	0.016	NA	NA	6.000	-1184.741	2381.727	5.867

Table S15. Model selection comparisons: *Pr(z, h, w)*, *BB* genotype.

Int.	*h*	*w*	*z*	*h*×*w*	*h*×*z*	*w*×*z*	*w*×*h*×*z*	df	logLik	AICc	delta
-2.425	NA	NA	0.294	NA	NA	NA	NA	2.000	-184.892	373.798	0.000
-2.716	NA	2.465	0.293	NA	NA	NA	NA	3.000	-184.715	375.459	1.661
-2.425	0.000	NA	0.294	NA	NA	NA	NA	3.000	-184.892	375.812	2.015
-3.440	NA	8.394	0.369	NA	NA	-0.612	NA	4.000	-184.470	376.989	3.191
-2.552	0.023	NA	0.307	NA	-0.002	NA	NA	4.000	-184.626	377.300	3.502
-2.721	-0.002	2.621	0.293	NA	NA	NA	NA	4.000	-184.704	377.456	3.658
-3.476	-0.003	8.851	0.372	NA	NA	-0.635	NA	5.000	-184.444	378.961	5.163
-2.792	0.019	2.234	0.305	NA	-0.002	NA	NA	5.000	-184.493	379.059	5.261
-2.645	-0.023	1.973	0.294	0.142	NA	NA	NA	5.000	-184.681	379.436	5.638
-3.435	0.014	7.716	0.371	NA	-0.002	-0.552	NA	6.000	-184.305	380.711	6.914
-3.398	-0.028	8.193	0.375	0.175	NA	-0.648	NA	6.000	-184.411	380.925	7.127
-2.806	0.023	2.329	0.305	-0.022	-0.002	NA	NA	6.000	-184.492	381.087	7.289
-5.110	0.416	20.159	0.534	-2.859	-0.037	-1.783	0.260	8.000	-182.471	381.118	7.321
-3.418	0.007	7.607	0.372	0.043	-0.002	-0.559	NA	7.000	-184.303	382.742	8.944
-1.664	0.272	26.209	NA	-2.038	NA	NA	NA	4.000	-350.559	709.166	335.368
-0.473	NA	16.730	NA	NA	NA	NA	NA	2.000	-357.386	718.786	344.988
-0.459	-0.010	17.069	NA	NA	NA	NA	NA	3.000	-357.068	720.165	346.367
1.628	NA	NA	NA	NA	NA	NA	NA	1.000	-370.008	742.020	368.222
1.642	-0.003	NA	NA	NA	NA	NA	NA	2.000	-369.987	743.988	370.190

Table S16. Model selection comparisons: *Pr(z, h, w)*, *MM* genotype.

Int.	*h*	*w*	*z*	*h*×*w*	*h*×*z*	*w*×*z*	*w*×*h*×*z*	df	logLik	AICc	delta
-4.078	-0.251	18.706	0.601	1.573	NA	-2.765	NA	6.000	-268.129	548.333	0.000
-4.052	-0.258	18.873	0.597	1.537	0.001	-2.764	NA	7.000	-268.089	550.279	1.946
-4.538	-0.039	22.169	0.567	NA	NA	-2.518	NA	5.000	-270.473	551.000	2.667
-3.880	-0.314	17.612	0.583	1.950	0.005	-2.667	-0.030	8.000	-268.065	552.259	3.926
-4.457	-0.073	22.626	0.562	NA	0.002	-2.561	NA	6.000	-270.151	552.377	4.044
-4.596	NA	21.613	0.563	NA	NA	-2.522	NA	4.000	-273.296	554.627	6.294
-0.449	-0.169	-10.150	0.241	0.970	NA	NA	NA	5.000	-276.884	563.821	15.488
-0.943	-0.042	-6.289	0.241	NA	NA	NA	NA	4.000	-277.895	563.826	15.493
-1.624	-0.045	NA	0.237	NA	NA	NA	NA	3.000	-279.389	564.799	16.465
-0.878	-0.057	-6.311	0.237	NA	0.001	NA	NA	5.000	-277.835	565.723	17.390
-0.436	-0.172	-10.112	0.240	0.962	0.000	NA	NA	6.000	-276.879	565.833	17.500
-1.569	-0.058	NA	0.233	NA	0.001	NA	NA	4.000	-279.341	566.718	18.385
-0.971	NA	-7.106	0.236	NA	NA	NA	NA	3.000	-281.000	568.020	19.687
-1.750	NA	NA	0.231	NA	NA	NA	NA	2.000	-282.951	569.912	21.579
0.674	NA	9.562	NA	NA	NA	NA	NA	2.000	-442.944	889.899	341.566
0.698	-0.018	10.050	NA	NA	NA	NA	NA	3.000	-442.114	890.250	341.917
1.089	-0.114	6.959	NA	0.743	NA	NA	NA	4.000	-441.284	890.604	342.271
1.853	NA	NA	NA	NA	NA	NA	NA	1.000	-447.923	897.850	349.517
1.912	-0.012	NA	NA	NA	NA	NA	NA	2.000	-447.500	899.011	350.678

Table S17. Model selection comparisons: *R(z, h, w)*, *BB* genotype.

Int.	*h*	*w*	*z*	*h*×*w*	*h*×*z*	*w*×*z*	*w*×*h*×*z*	df	logLik	AICc	delta
-45.659	-0.971	233.350	7.619	NA	0.080	-18.094	NA	7.000	-3874.306	7762.776	0.000
-12.211	-0.916	NA	5.078	NA	0.077	NA	NA	5.000	-3876.768	7763.624	0.848
2.557	-0.817	-132.583	5.173	NA	0.075	NA	NA	6.000	-3875.937	7763.996	1.220
-37.432	-2.751	169.731	7.643	13.051	0.080	-18.180	NA	8.000	-3873.984	7764.178	1.403
10.809	-2.554	-196.358	5.186	12.735	0.075	NA	NA	7.000	-3875.631	7765.426	2.650
-55.596	1.047	302.514	8.530	-13.912	-0.104	-24.615	1.310	9.000	-3873.622	7765.507	2.731
-46.420	0.827	167.250	7.686	NA	NA	-15.664	NA	6.000	-3877.583	7767.289	4.514
-4.257	0.856	-148.554	5.546	NA	NA	NA	NA	5.000	-3878.806	7767.698	4.923
-21.141	0.811	NA	5.454	NA	NA	NA	NA	4.000	-3879.845	7767.748	4.972
-38.328	-0.922	104.566	7.710	12.838	NA	-15.745	NA	7.000	-3877.275	7768.713	5.937
3.881	-0.857	-211.544	5.559	12.574	NA	NA	NA	6.000	-3878.510	7769.143	6.367
-46.649	NA	215.130	7.796	NA	NA	-16.948	NA	5.000	-3880.416	7770.920	8.144
-15.462	NA	NA	5.402	NA	NA	NA	NA	3.000	-3882.583	7771.201	8.426
-0.906	NA	-125.722	5.478	NA	NA	NA	NA	4.000	-3881.839	7771.736	8.961
48.746	NA	596.586	NA	NA	NA	NA	NA	3.000	-4102.823	8211.681	448.905
49.479	-0.229	600.299	NA	NA	NA	NA	NA	4.000	-4102.707	8213.471	450.696
37.869	2.182	686.455	NA	-17.674	NA	NA	NA	5.000	-4102.399	8214.886	452.110
127.328	NA	NA	NA	NA	NA	NA	NA	2.000	-4112.324	8228.666	465.890
127.919	-0.111	NA	NA	NA	NA	NA	NA	3.000	-4112.298	8230.630	467.854

Table S18. Model selection comparisons: *R(z, h, w)*, *MM* genotype.

Int.	*h*	*w*	*z*	*h×w*	*h×z*	*w×z*	*w×h×z*	df	logLik	AICc	delta
-39.965	-1.094	314.501	6.797	NA	0.102	-19.790	NA	7.000	-5326.713	10667.543	0.000
-38.674	-1.495	303.680	6.809	3.131	0.101	-19.837	NA	8.000	-5326.688	10669.524	1.982
3.013	-1.029	NA	4.083	NA	0.099	NA	NA	5.000	-5330.447	10670.957	3.414
11.781	-0.955	-88.125	4.176	NA	0.097	NA	NA	6.000	-5329.689	10671.465	3.922
-41.221	-0.740	322.444	6.933	-2.342	0.065	-20.740	0.260	9.000	-5326.677	10671.540	3.998
12.803	-1.244	-96.640	4.180	2.263	0.097	NA	NA	7.000	-5329.676	10673.467	5.924
-44.826	1.339	276.512	7.029	NA	NA	-18.349	NA	6.000	-5331.477	10675.040	7.497
-41.289	0.218	248.757	7.055	8.295	NA	-18.508	NA	7.000	-5331.296	10676.707	9.165
-6.241	1.326	NA	4.486	NA	NA	NA	NA	4.000	-5334.921	10677.883	10.340
3.517	1.365	-96.435	4.581	NA	NA	NA	NA	5.000	-5334.019	10678.101	10.558
6.986	0.383	-123.609	4.586	7.273	NA	NA	NA	6.000	-5333.881	10679.848	12.306
-46.871	NA	337.057	7.235	NA	NA	-19.790	NA	5.000	-5341.330	10692.722	25.179
-1.318	NA	NA	4.531	NA	NA	NA	NA	3.000	-5344.631	10695.287	27.744
5.306	NA	-64.494	4.595	NA	NA	NA	NA	4.000	-5344.232	10696.505	28.963
41.098	1.472	484.835	NA	NA	NA	NA	NA	4.000	-5544.044	11096.129	428.587
37.995	2.341	508.368	NA	-6.436	NA	NA	NA	5.000	-5543.973	11098.009	430.466
43.151	NA	521.202	NA	NA	NA	NA	NA	3.000	-5551.784	11109.592	442.050
101.635	1.712	NA	NA	NA	NA	NA	NA	3.000	-5560.704	11127.433	459.890
109.399	NA	NA	NA	NA	NA	NA	NA	2.000	-5570.919	11145.851	478.309

Supplementary Figures

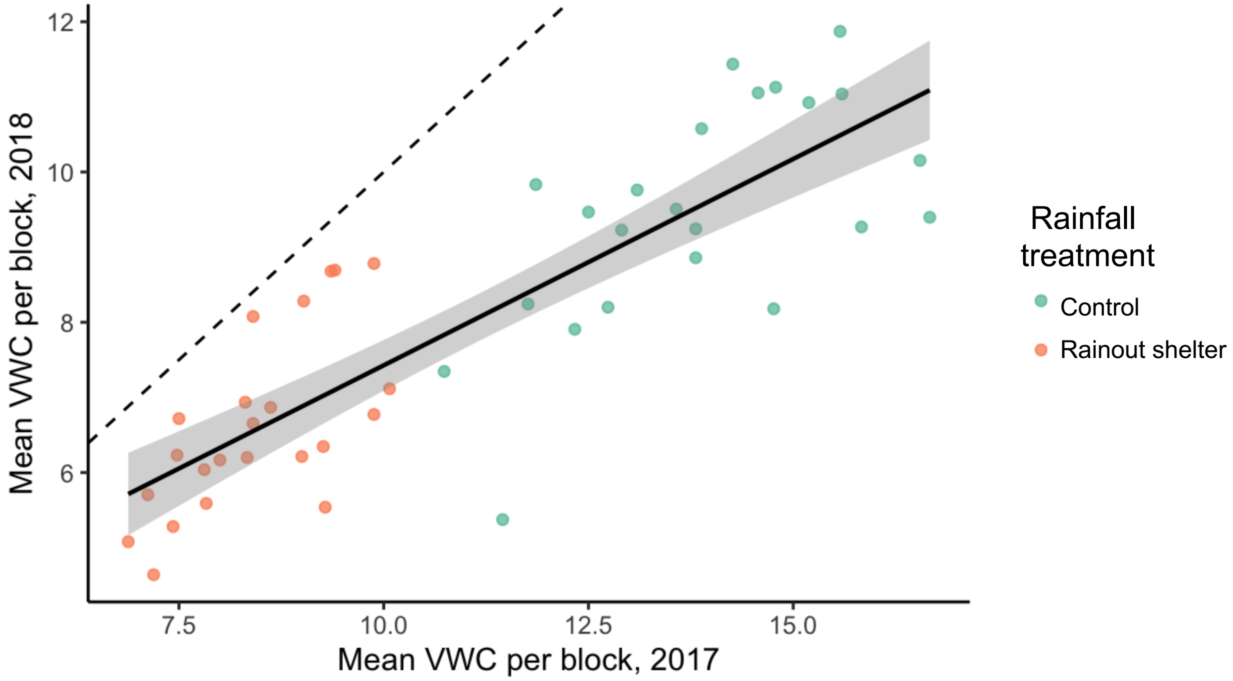

Supplementary Figure 1. Justification for predicting unobserved soil moisture values. Within our experimental garden, individual blocks retained similar dryness rank across years, even if overall mean VWC differed from year to year (*e.g.,* here, 2018 was drier than 2017, as all points fall below the dashed 1:1 line). Block-level consistency in rank soil moisture within the garden permits approximation of unobserved values from off-site mean VWC values. Here, each point represents one block's mean soil moisture (VWC: volumetric water content) in 2017 (x-axis) and 2018 (y-axis), and the color of the point indicates whether the block was subject to rainfall exclusion using rainout shelters vs. the control treatment.

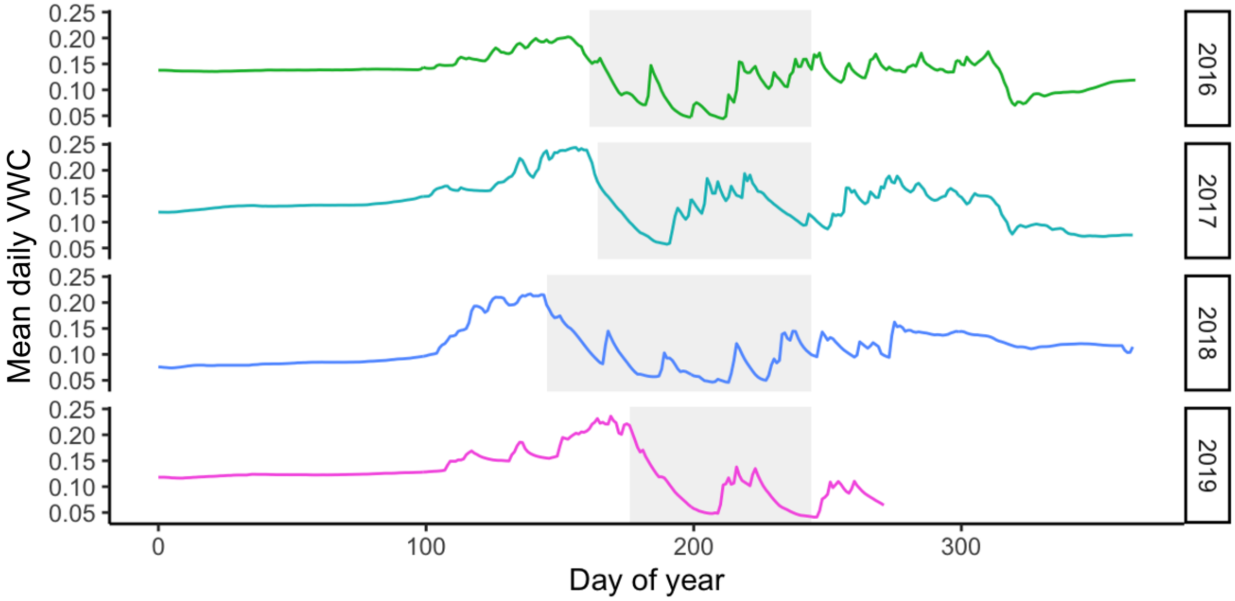

Supplementary Figure 2. Off-site daily mean soil moisture across four growing seasons. Dataloggers deployed at another research site 0.5 km from our transplant garden collected continuous data on soil moisture (VWC: volumetric water content) throughout the duration of our 4-year study (J. T. Anderson, *unpubl. data*). In each row, which represents one year of this experiment, traces show the daily VWC estimated as the mean of all VWC measurements taken at each time point throughout the day. Gray boxes denote the time periods considered to be the growing season for the purposes of this experiment, beginning on the date of snowmelt and ending on September 1. Within each growing season, we used the daily mean off-site VWC as the independent variable in our linear models to predict soil moisture in our transplant blocks on days in which it was not directly observed.

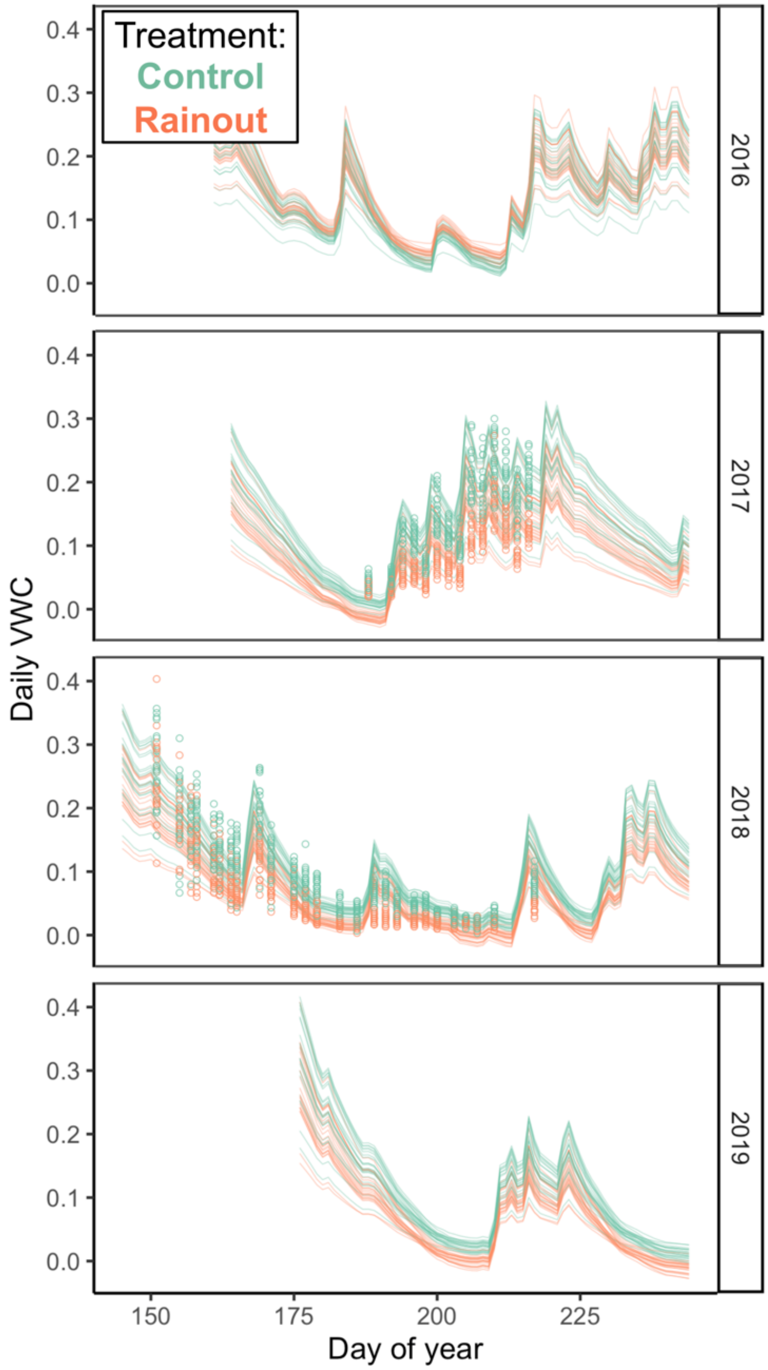

Supplementary Figure 3. Seasonal variation in block-level soil moisture across experimental treatments. Solid lines show daily values of soil moisture (VWC: volumetric water content) predicted from models using continuous variation in soil moisture at a nearby site (**Supplementary Figure 2**; **Appendix S1**). Open circles show VWC values directly measured in each garden block during the four growing seasons of this study (date of snowmelt through September 1). Colored lines and points denote blocks that were subject to rainfall reduction using rainout shelters (orange) vs. controls (green). Rainout shelter manipulations spanned 2017-2019, so there is no effect of rainfall treatment in 2016.

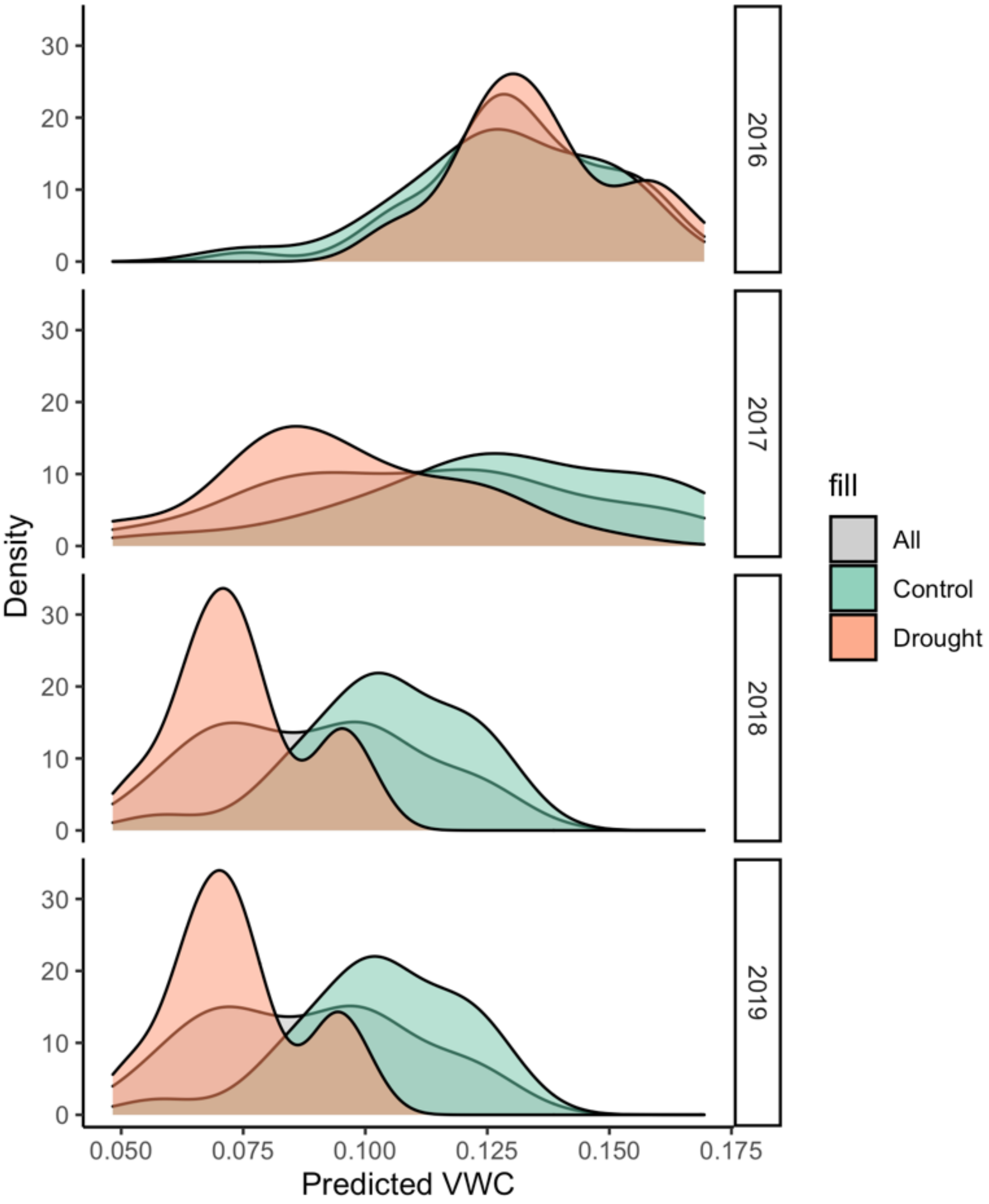

Supplementary Figure 4. Distribution of growing season mean soil moisture (volumetric water content; VWC) across blocks in the common garden. Distributions are shown separately for blocks with different rainfall treatment assignments (green: control blocks; orange: blocks with rainout shelters) with the overall distribution regardless of treatment identity shown in gray. Rainout treatments began in 2017, so distributions are heavily overlapping in 2016.

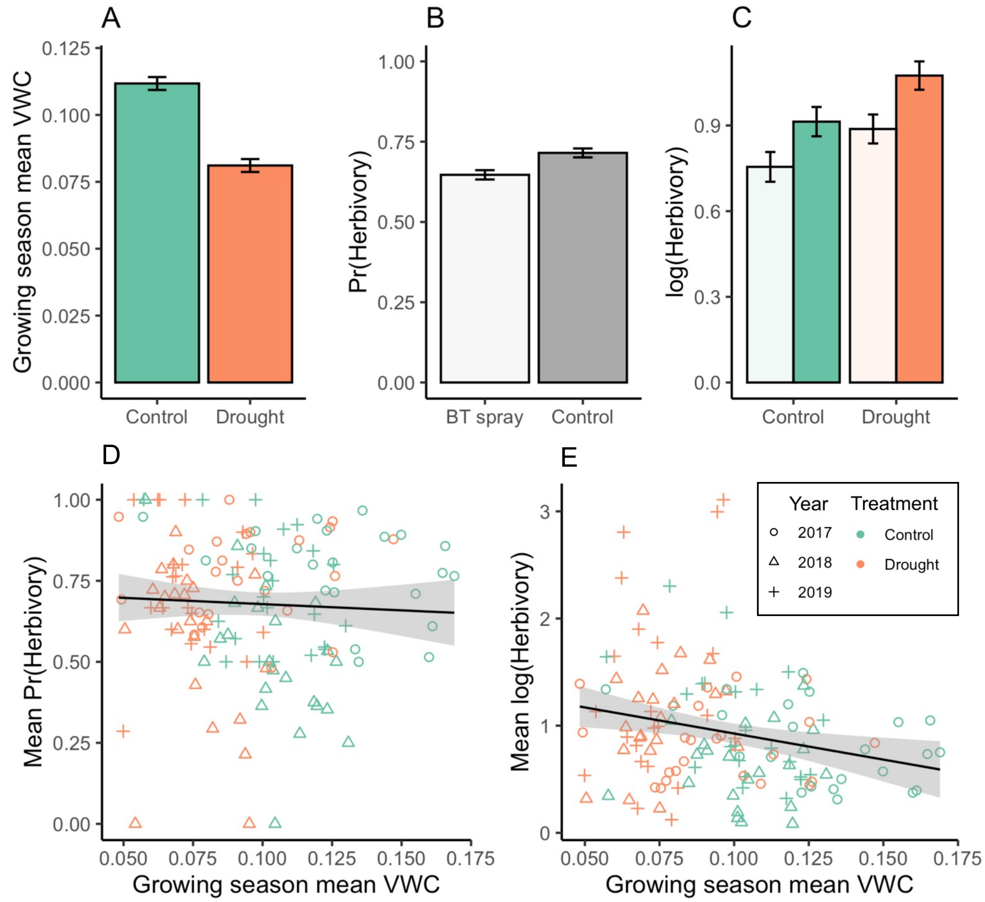

Supplementary Figure 5. Experimental manipulations significantly altered environmental drivers of selection on defensive chemistry. A: Rainout shelters reduced growing season soil moisture from ambient levels (main effect of drought over three manipulated field seasons: F = 80.3481, P = 2.626 × 10^{-15}, N = 138). **B:** Application of BT spray, a general insecticide, reduced the probability that individual plants received herbivore damage (main effect of insecticide treatment over three manipulated field seasons: χ^2 = 13.639, P = 0.0002, N = 2,474). **C:** Among plants that received herbivore damage, BT spray also reduced the amount of tissue lost to herbivore consumption (main effect of insecticide treatment over three manipulated field seasons; F = 12.4199, P = 0.0004, N = 1,729). On average, herbivory levels were also higher in plants subjected to elevated drought stress. **D:** Across treatments, the mean probability of individuals in an experimental block receiving herbivory was not correlated with block-level mean soil moisture. **E:** The mean amount of herbivore damage per individual in experimental blocks was negatively correlated with block-level mean soil moisture (r = -0.2375, F = 7.8935, P = 0.0057, N = 134). In all panes, the color of bars or symbols indicates the rainfall treatment (green: control blocks; orange: rainout shelters). In B and C, the opacity of the bars represents the herbivore pressure treatment (dark shading: ambient conditions; light shading: herbivory reduction using insecticide spray). In D and E, the symbol shapes denote the year of data collection during the field experiment, including only years in which driver manipulations were implemented (2017-2019).

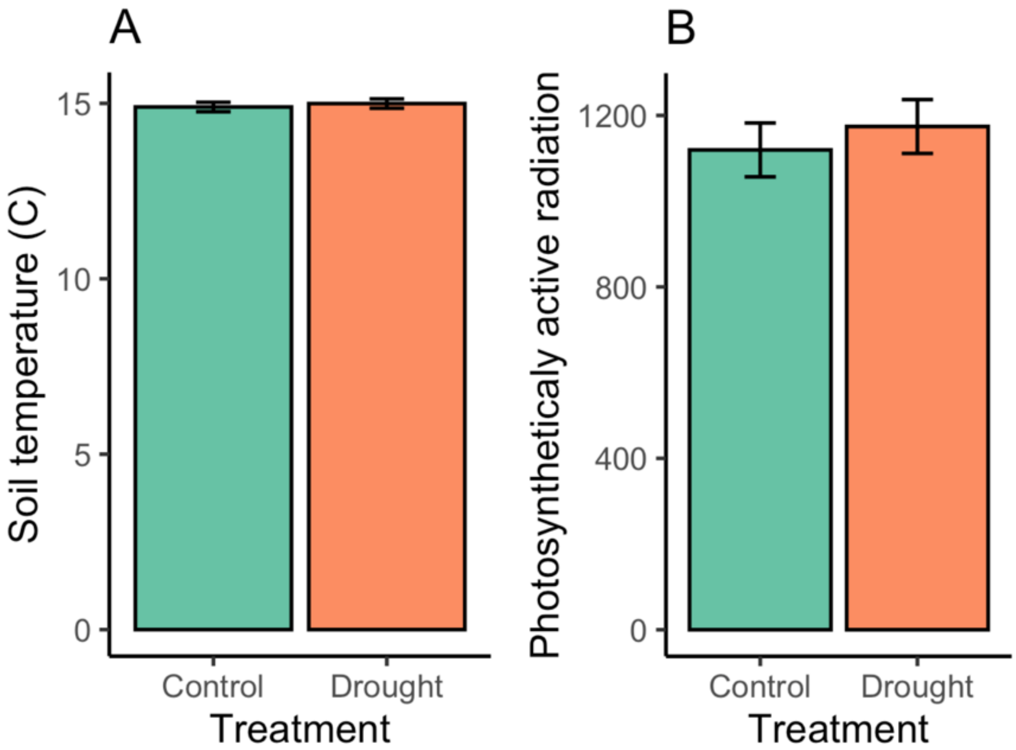

Supplementary Figure 6. Experimental manipulations of soil moisture did not alter soil temperature (A) or light availability (B). Main effect of rainout shelter treatment on soil temperature: $F = 0.1251$, $P = 0.7236$, N = 1,824. Main effect of rainout shelter treatment on light availability: $F = 0.3790$, $P = 0.5392$, N = 138.

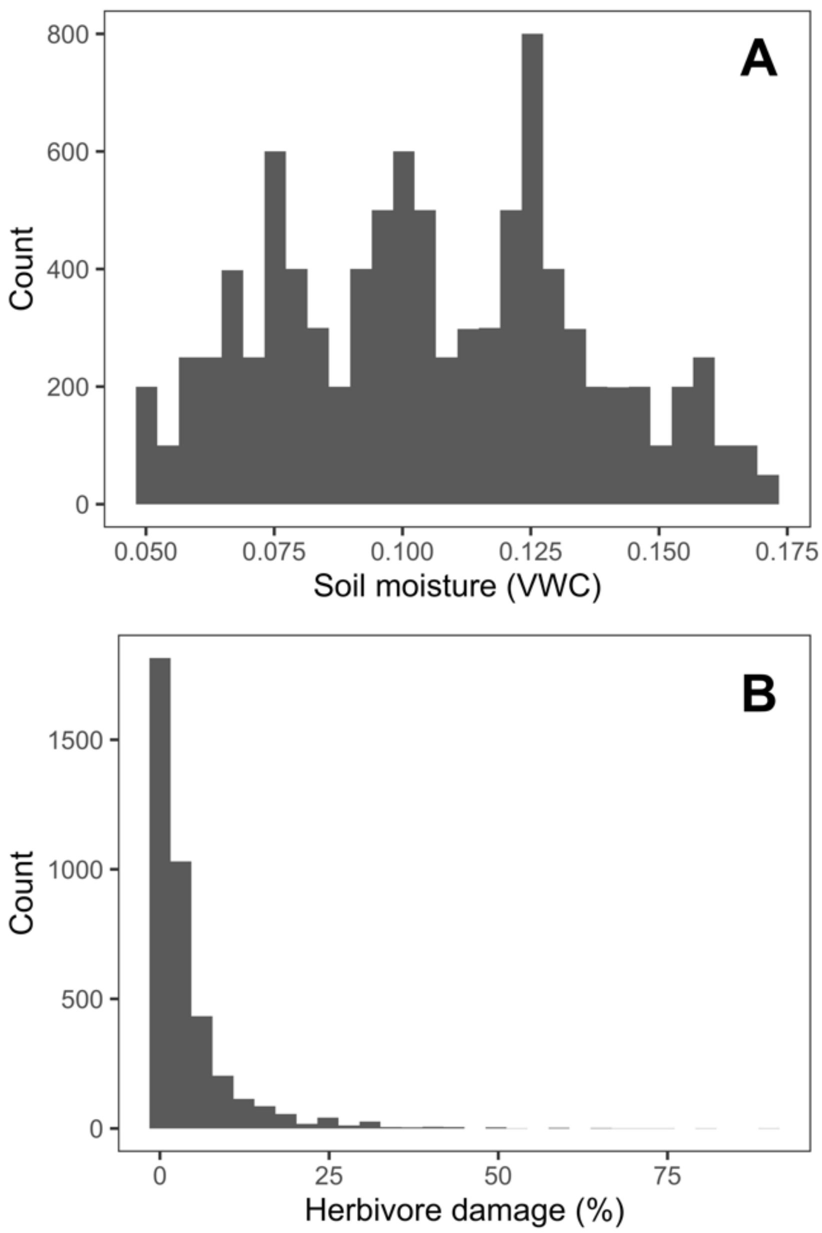

Supplementary Figure 7. Distributions of driver values in the field experiment. A: Soil moisture was generally Normally distributed, with multiple modes reflecting variation induced by rainout shelter treatments. **B**: Herbivory was skewed toward low values, although observed damage levels ranged up to 90% total leaf area removal.

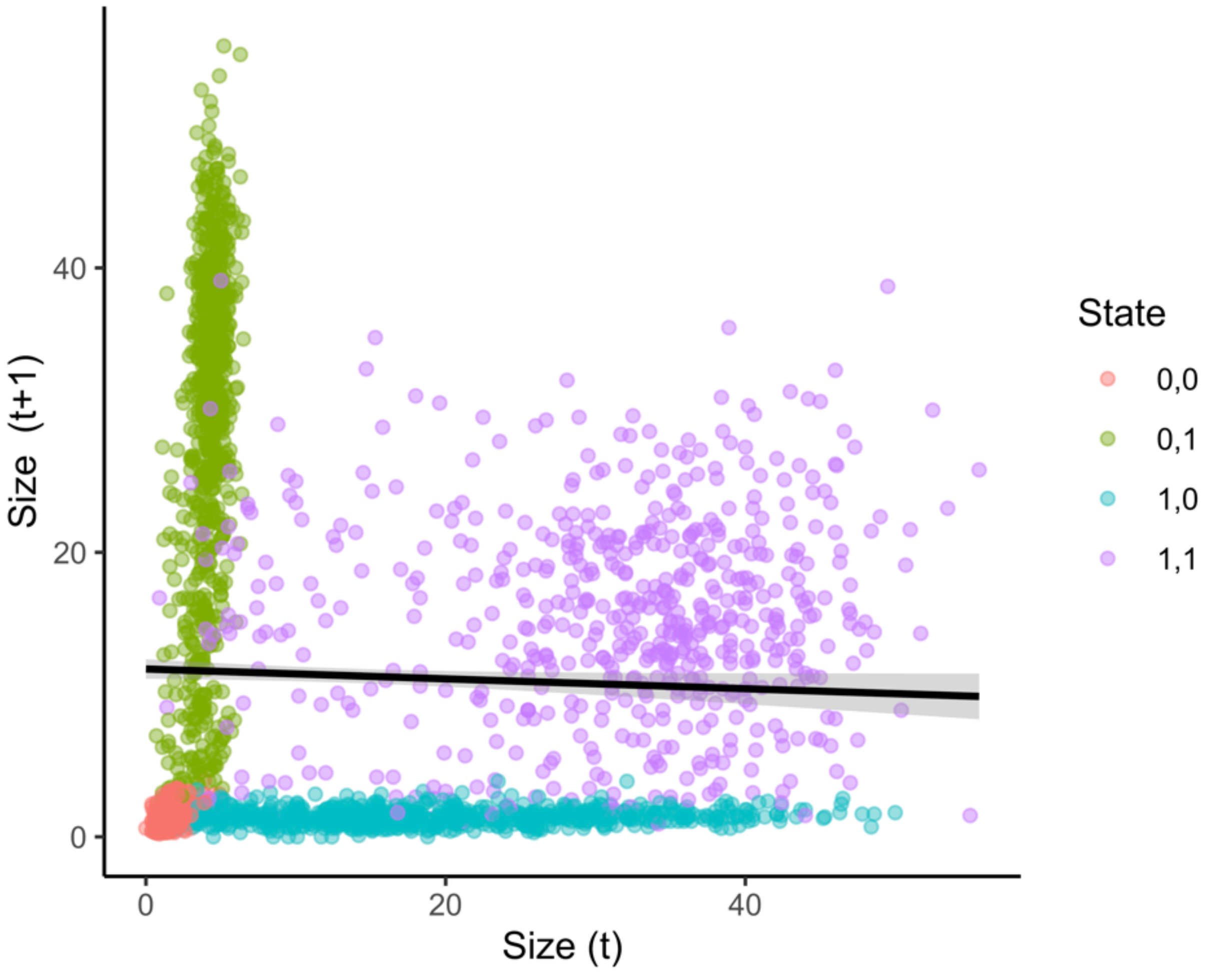

Supplementary Figure 8. A single growth function across all bolting states yields poor fit and wide variance in growth residuals. Color of points represents bolting states at times *t, t*+1.

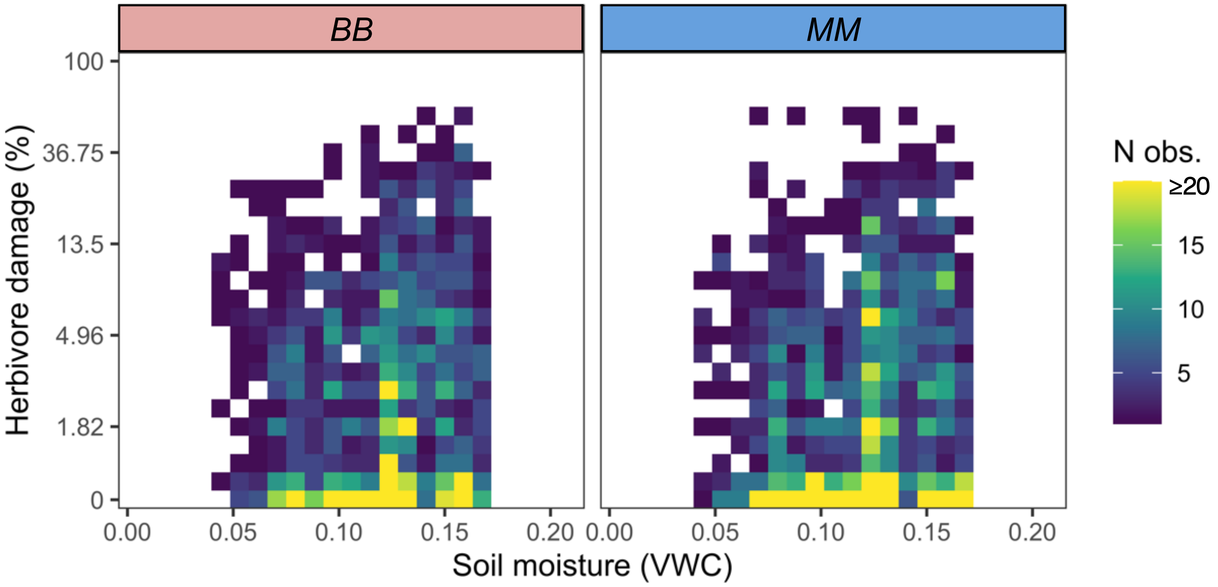

Supplementary Figure 9. Heatmap showing combinations of drought and herbivory that experienced by transplants during the common garden experiment. Higher numbers of plant-year observations experiencing a given combination of drought and herbivory are indicated in yellow, and lower observed numbers of plant-years experiencing a given combination of drought and herbivory are indicated in blue. Modeled combinations of drought and herbivory that were not experienced by transplants during our common garden experiment are shown in white.

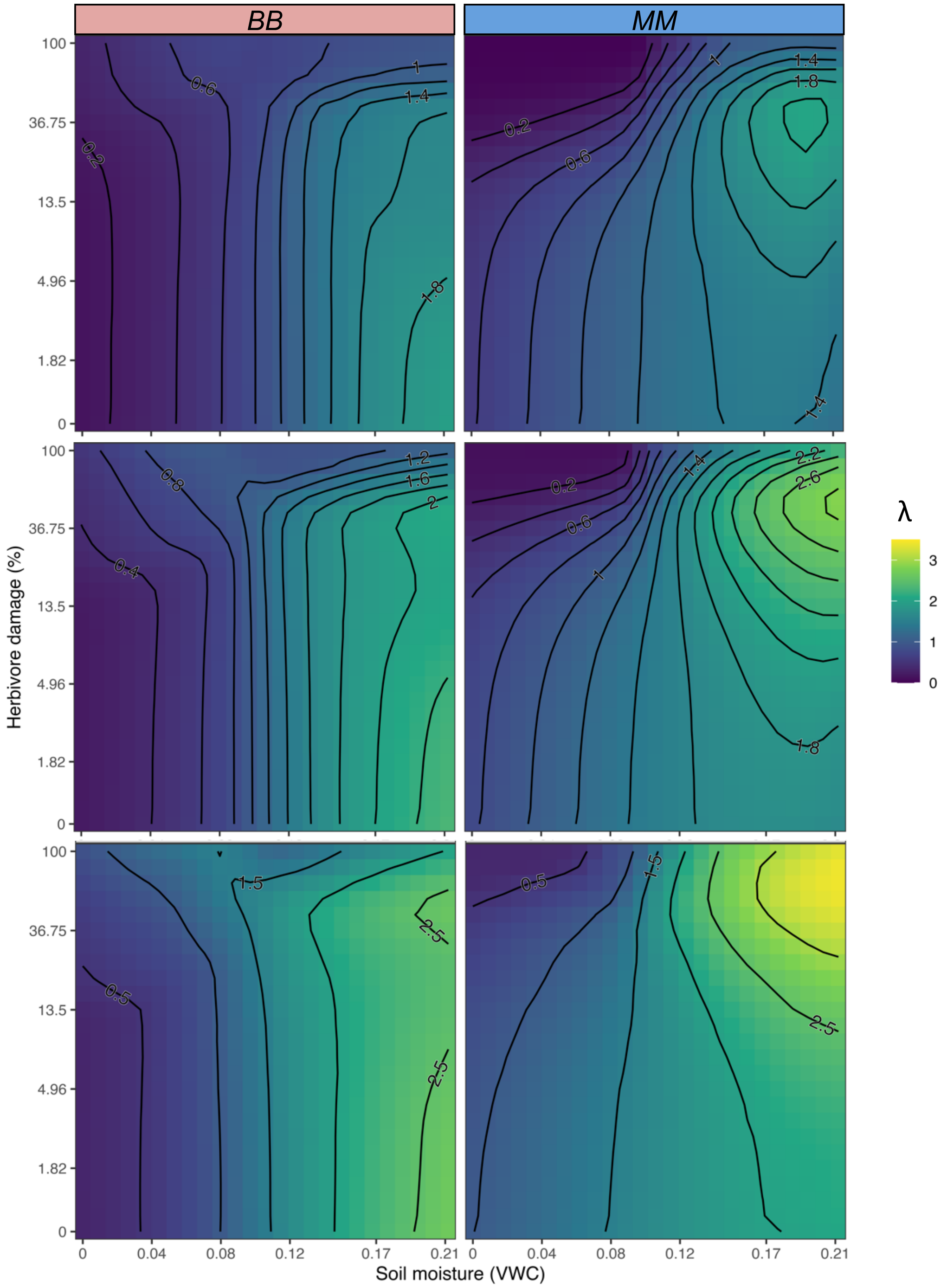

Supplementary Figure 10. Total fitness topographies for each glucosinolate NIL, accounting for uncertainty in vital rate regression parameters. Heatmaps show variation in the lower 95th confidence limit (top), median (middle), and upper 95th confidence limit (bottom) of lifetime fitness (λ) across 1,000 bootstrapped simulations sampling vital rate parameters from their distributions. λ was integrated across the life cycle over 4 years under field conditions, separately for each focal genotype (left: *BB* = branched-chain glucosinolate homozygote; right: *MM* = methionine glucosinolate homozygote). A total fitness of 1 equates to demographic replacement.

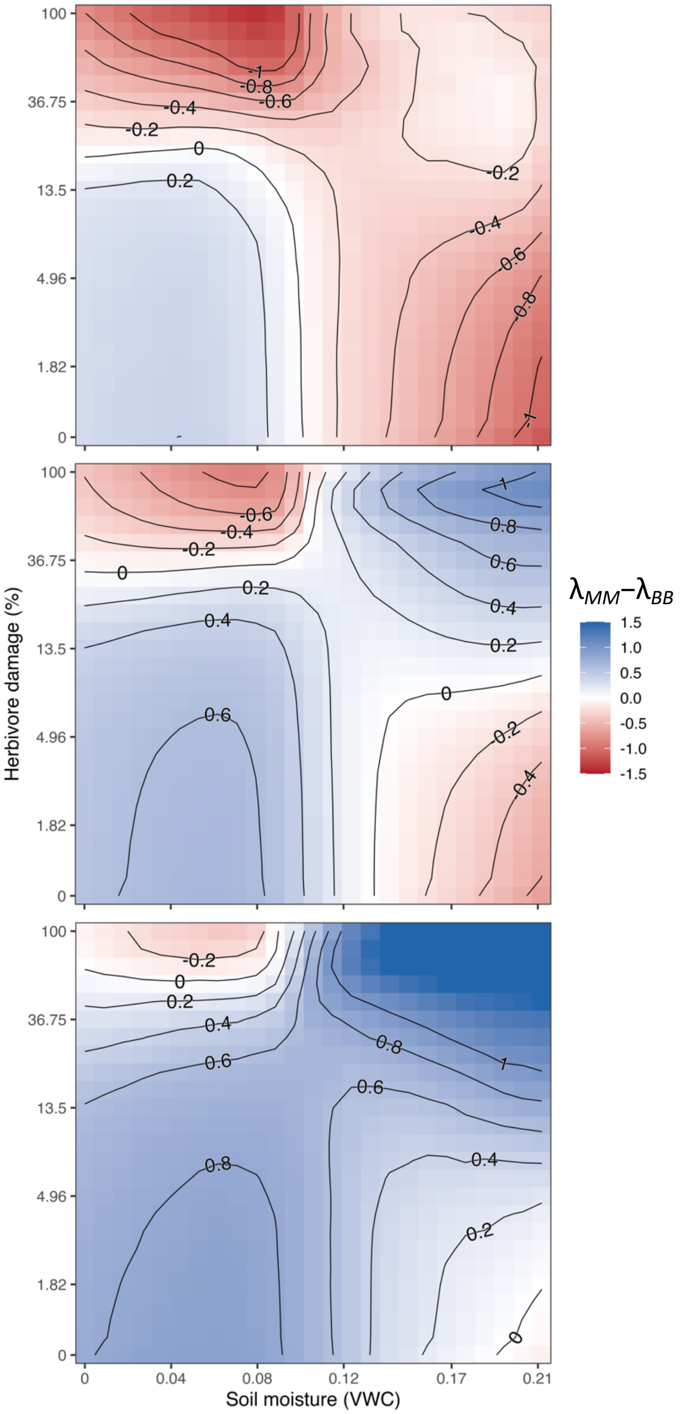

Supplementary Figure 11. Heatmaps showing variation in the lower 95th confidence limit (top), median (middle) and lower 95th confidence limit (bottom) of the difference in lifetime fitness between *MM* and *BB* genotypes in each environment space. Confidence limits were calculated from 1,000 bootstrap simulations sampling vital rate parameters from their distributions. Either genotype may have higher lifetime fitness in wet soils, but in drier environments, *MM* has consistently higher lifetime fitness at low herbivory and *BB* has consistently higher lifetime fitness at high herbivory.

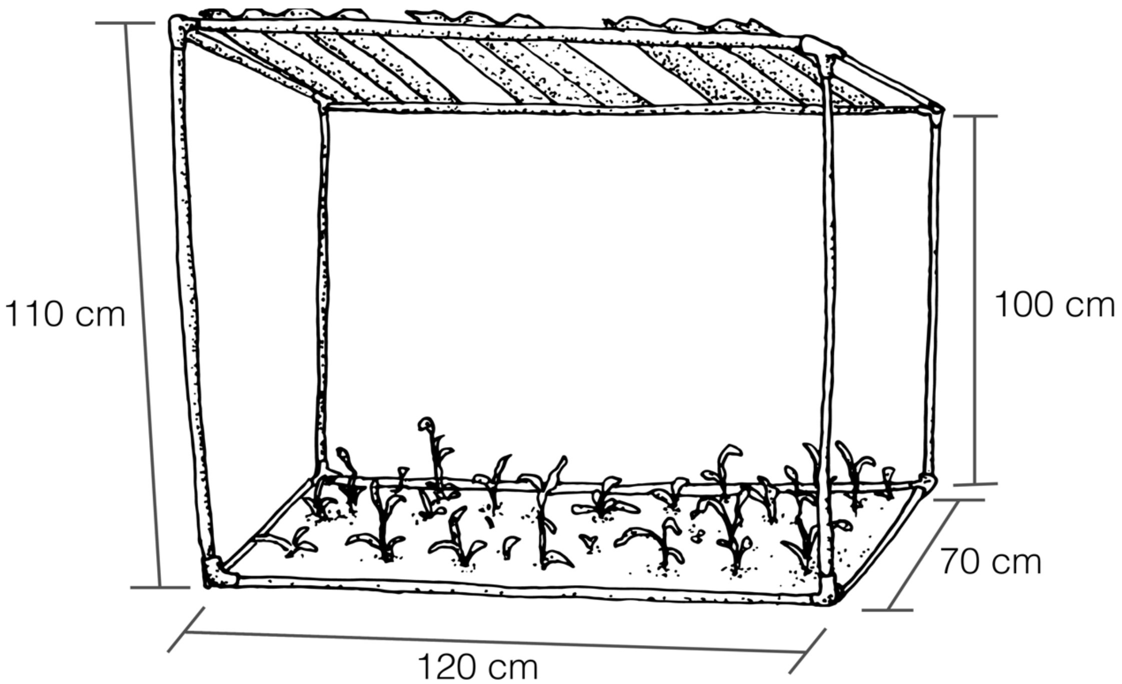

Supplementary Figure 12. Rainout shelter design. Shelters covered 100 x 50 cm garden blocks plus a 10 cm buffer on all sides. Narrow trenches lined with a plastic moisture barrier (not shown) also extended 10 cm belowground to reduce lateral water flow among blocks (not shown). Illustration by Erin Fox.

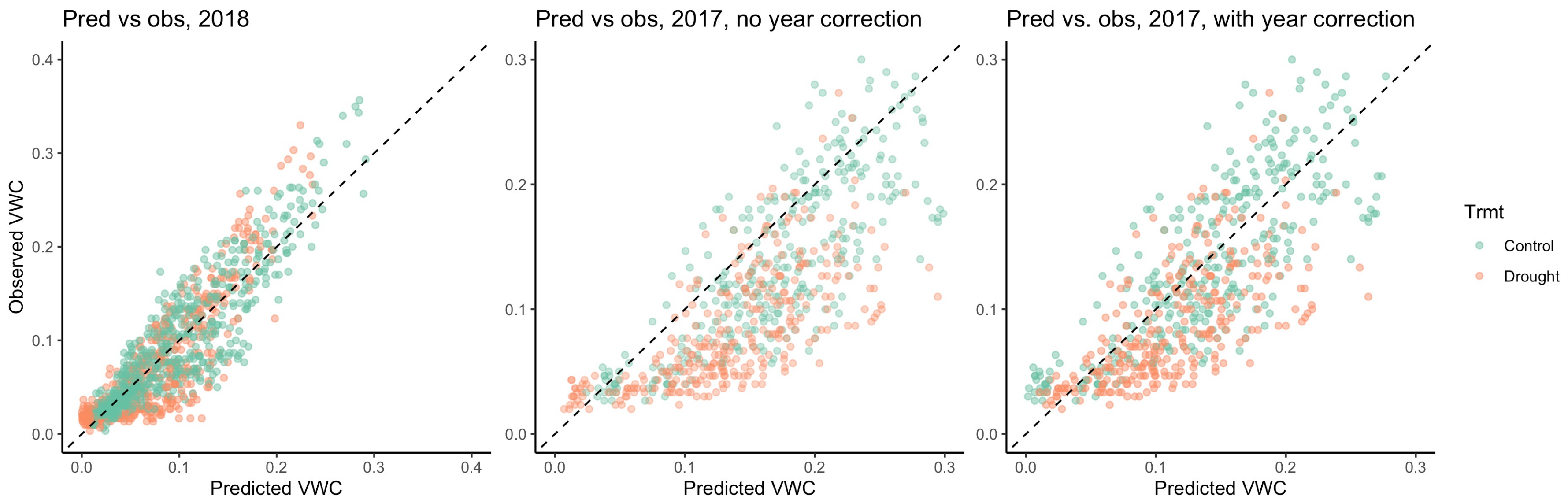

Supplementary Figure 13. Predicted vs. observed soil moisture values in 2018 (left) and 2017, with (right) and without (center) correcting the intercept with the difference in mean soil moisture across years (Appendix S1).

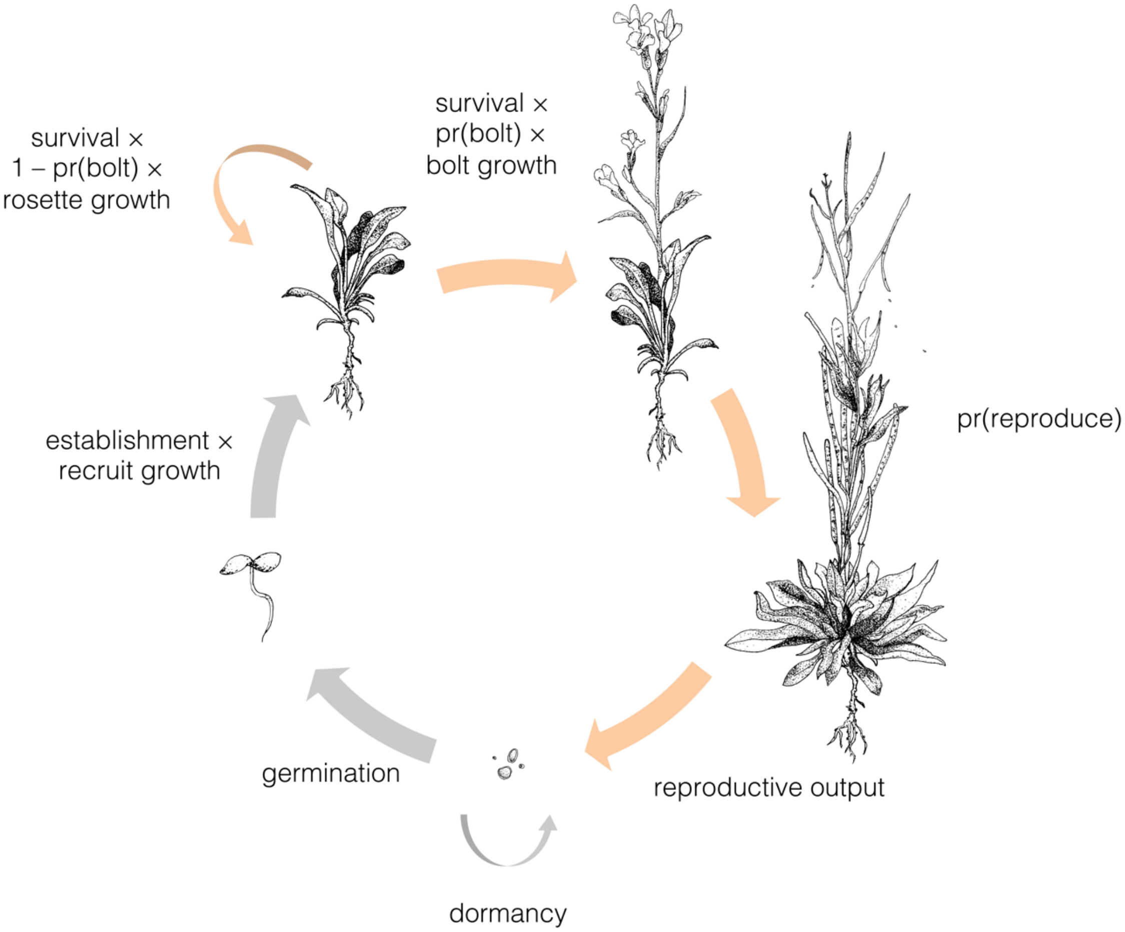

Supplementary Figure 14. Visualization of the demographic model. Orange arrows represent vital rates that we modeled as size- and driver-dependent. Gray arrows represent vital rates that we modeled as size- and driver-independent. We modeled all driver-dependent vital rates, as well as driver-independent recruit size, separately for each genotype to incorporate effects of genetic variation on fitness. An additional vital rate, variance in growth, is not shown in this schematic but was modeled as size- and driver- dependent. Illustrations by Erin Fox.

Supplementary Tables

Supplementary Table 1. Summary of genotypes and family lines (**Methods; Appendix S1**) used in the demographic field experiment.

Genotype	Family line	Cohort		Total
		2015	2016	
BB	9	150	0	150
	13	150	0	150
	14	0	100	100
	15	150	0	150
	19	150	0	150
	35	150	100	250
	36	0	100	100
	91	0	100	100
MM	8	150	0	150
	20	150	0	150
	21	150	0	150
	23	150	100	250
	25	150	100	250
	31	0	100	100
	38	0	100	100
**TOTALS:**		**1,500**	**800**	**2,300**

Supplementary Table 2. Ambient environmental conditions during the demographic common garden experiment.

Year	Summary	Winter precip. (cm)[1]	Max snow water eq. (cm)[2]	Melt date (ordinal)[3]	Average summer air temp. (°C)[4]			Summer precip. (cm)[4]	Summer soil moisture (m^3/m^3)[5]		
					min	mean	max		min	mean	max
2016	Moderate winter, wet summer	90.7	70.1	161	5.4	10.9	18.5	9.9	0.0424	0.1029	0.2128
2017	Wet winter, wet summer	134.6	126.5	164	5.4	10.9	19.0	10.2	0.0552	0.1251	0.2274
2018	Dry winter, dry summer	74.4	72.9	145	5.3	11.6	19.8	5.6	0.0437	0.0940	0.2010
2019	Wet winter, dry summer	134.1	128.5	176	6.0	11.9	19.9	3.8	0.0406	0.0937	0.2596

[1]Sum of daily precipitation between September 1 of the preceding year and the day of snowmelt in the current year. Data from SNOTEL sensor site #737, located 2.25 km SW of the experimental garden at 3261m elevation (USDA NRCS 2020).
[2]Maximum value of snow water equivalent (theoretical volume of water released if all snowpack melted simultaneously) occurring between September 1 of the preceding year and day of snowmelt in the current year (USDA NRCS 2020).
[3]Snowmelt date in Schofield Park, rounded to nearest ordinal day estimated in Anderson & Wadgymar 2020 (2016-2018) or via personal observation (2019).
[4]Daily air temperature (averages) and total precipitation (sum) occurring between the snowmelt date and September 1 of each year (USDA NRCS 2020).
[5]Soil moisture data from Schofield Park taken using Decagon 5TM probes deployed at 10cm depth from the date of snowmelt until September of the current year (J. T. Anderson, *unpubl. data*).

Supplementary Table 3. Experimental design for driver manipulations. Because manipulations began in year 2 of the study, plants which had already died prior to June 2017 (436 *BB* and 259 *MM* individuals) were unmanipulated and experienced ambient conditions in their only year of life. This left 1,605 of 2,300 *B. stricta* transplants to experience driver manipulations, across all treatments and genotypes. N_{BB} and N_{MM} refer to the number of replicate individuals of the homozygous branched-chain glucosinolate and homozygous methionine glucosinolate NILs, respectively, in each treatment combination.

		**Soil moisture manipulation**	
		**Ambient (23 blocks)**	**Reduced (23 blocks)**
**Herbivory manipulation (individual plants)**	**Ambient**	N_{BB} = 180<br>N_{MM} = 223	N_{BB} = 176<br>N_{MM} = 223
	**Reduced**	N_{BB} = 182<br>N_{MM} = 227	N_{BB} = 176<br>N_{MM} = 218

Supplementary Table 4. Number of replicates per genotype per treatment included in the demographic experiment.

Planting cohort	Garden block	Drought manipulation	Herbivory manipulation				Not manipulated (died before June 2017)	
			BT spray		Control			
			BB	MM	BB	MM	BB	MM
2015	1	Rainout	6	3	5	5	13	18
	2	Control	9	7	8	7	9	10
	3	Rainout	11	11	12	11	3	2
	4	Control	8	12	8	12	8	2
	5	Control	7	9	7	8	12	7
	6	Rainout	10	11	11	11	3	4
	7	Control	11	10	11	9	7	2
	8	Rainout	7	11	8	11	6	7
	9	Rainout	8	7	7	7	11	10
	10	Control	9	9	10	9	5	8
	11	Rainout	11	13	11	12	2	1
	12	Control	8	11	9	11	9	2
	13	Control	5	6	5	6	14	14
	14	Rainout	11	11	10	12	5	1
	15	Control	12	11	11	11	5	0
	16	Rainout	8	10	8	10	6	8
	17	Rainout	11	8	11	8	6	6
	18	Control	8	11	8	11	6	6
	19	Rainout	7	11	8	11	11	2
	20	Control	7	11	7	11	10	4
	21	Control	8	12	7	12	7	4
	22	Rainout	10	7	8	8	10	7
	23	Control	8	10	8	11	10	3
	24	Rainout	5	7	6	6	13	13
	25	Rainout	11	6	9	9	7	8
	26	Control	8	12	8	11	7	4
	27	Rainout	5	9	6	8	12	10
	28	Control	4	5	5	5	18	13
	29	Rainout	6	11	6	11	9	8
	30	Control	8	8	8	7	13	4
2016	1	Control	10	10	10	11	9	0
	2	Rainout	10	10	10	10	6	4
	3	Control	9	12	8	10	5	6
	4	Rainout	8	13	8	14	7	0
	5	Rainout	7	11	7	11	10	4
	6	Control	6	10	5	10	15	4
	7	Rainout	4	11	5	11	11	8
	8	Control	9	8	8	8	13	4
	9	Control	6	10	6	10	14	4
	10	Rainout	5	7	5	7	16	10
	11	Control	6	13	7	12	10	2
	12	Rainout	8	11	8	12	9	2
	13	Rainout	4	11	4	11	16	4
	14	Control	8	10	8	10	7	7
	15	Rainout	3	8	3	7	19	10
	16	Control	8	10	8	11	12	1

Supplementary Table 5. Best-fit vital rate regressions retained after model selection. Driver- and size-sensitive models may include effects of size (*z*), herbivory (*h*), soil moisture (*w*), and interactions, or not, depending on model selection outcomes. Vital rate functions that were not driver-sensitive were estimated either from genotype-specific summary statistics (recruit size) or published values for this species under comparable field conditions (all others). For functions dependent on bolting, 0 and 1 represent “did not bolt” and “did bolt” in the indicated year, respectively. Intercept estimates are β_0, and $\beta_{i>0}$ are coefficients linking size and/or driver values to each vital rate response.

Fitness component (form)	Vital rate function	Geno.	Best-supported model	N
Adult survival (Binomial GLM)	$s(z_t, h, w)$	*BB*	$surv_{t+1} \sim \beta_0 + \beta_1 z_t + \beta_2 w_t$	1962
		MM	$surv_{t+1} \sim \beta_0 + \beta_1 z_t + \beta_1 w_t + \beta_2 h_t + \beta_3 w_t z_t + \beta_4 h_t z_t$	2187
Probability of bolting (Binomial GLM)	$b_0(z_t, h, w)$	*BB*	$bolt_{t+1} \mid bolt_t = 0 \sim \beta_0 + \beta_1 z_t + \beta_2 w_t + \beta_3 w_t z_t$	381
		MM	$bolt_{t+1} \mid bolt_t = 0 \sim \beta_0 + \beta_1 z_t$	366
	$b_1(z, h, w)$	*BB*	$bolt_{t+1} \mid bolt_t = 1 \sim \beta_0 + \beta_1 z_t + \beta_2 w_t + \beta_3 h_t + \beta_4 w_t z_t$	579
		MM	$bolt_{t+1} \mid bolt_t = 1 \sim \beta_0 + \beta_1 z_t + \beta_2 w_t + \beta_3 h_t + \beta_4 w_t z_t$	750
Growth (Gaussian LM)	$g_{00}(z_{t+1}, z_t, h, w)$	*BB*	$z_{t+1} \mid bolt_t = 0 \;\&\; bolt_{t+1} = 0 \sim \beta_0 + \beta_1 z_t + \beta_2 w_{t+1} + \beta_3 h_{t+1} + \beta_4 h_{t+1} w_{t+1}$	367
		MM	$z_{t+1} \mid bolt_t = 0 \;\&\; bolt_{t+1} = 0 \sim \beta_0 + \beta_1 z_t + \beta_2 w_{t+1} + \beta_3 h_{t+1}$	357
	$g_{01}(z_{t+1}, z_t, h, w)$	*BB*	$z_{t+1} \mid bolt_t = 0 \;\&\; bolt_{t+1} = 1 \sim \beta_0 + \beta_1 z_t + \beta_2 w_{t+1} + \beta_3 h_{t+1} + \beta_4 w_{t+1} z_t$	357
		MM	$z_{t+1} \mid bolt_t = 0 \;\&\; bolt_{t+1} = 1 \sim \beta_0 + \beta_1 z_t + \beta_2 h_{t+1} + \beta_3 h_{t+1} z_t$	369
	$g_{10}(z_{t+1}, z_t, h, w)$	*BB*	$z_{t+1} \mid bolt_t = 1 \;\&\; bolt_{t+1} = 0 \sim \beta_0 + \beta_1 w_{t+1}$	340
		MM	$z_{t+1} \mid bolt_t = 1 \;\&\; bolt_{t+1} = 0 \sim \beta_0 + \beta_1 z_t$	399
	$g_{11}(z_{t+1}, z_{t+1}, h, w)$	*BB*	$z_{t+1} \mid bolt_t = 1 \;\&\; bolt_{t+1} = 1 \sim \beta_0 + \beta_1 z_t + \beta_2 w_{t+1} + \beta_3 h_{t+1} + \beta_4 h_{t+1} w_{t+1} + \beta_5 h_{t+1} z_t$	239
		MM	$z_{t+1} \mid bolt_t = 1 \;\&\; bolt_{t+1} = 1 \sim \beta_0 + \beta_1 w_{t+1} + \beta_2 h_{t+1} + \beta_3 h_{t+1} w_{t+1}$	350
Variance in growth (Gaussian LM)	$v_{00}(z_t, h, w)$	*BB*	$Resid(g_{00})^2 \sim \beta_0$	367
		MM	$Resid(g_{00})^2 \sim \beta_0 + \beta_1 z_t + \beta_2 w_{t+1} + \beta_3 h_{t+1}$	357
	$v_{01}(z_t, h, w)$	*BB*	$Resid(g_{01})^2 \sim \beta_0 + \beta_1 w_{t+1}$	357
		MM	$Resid(g_{01})^2 \sim \beta_0$	369
	$v_{10}(z_t, h, w)$	*BB*	$Resid(g_{10})^2 \sim \beta_0 + \beta_1 z_t + \beta_2 w_{t+1} + \beta_3 w_{t+1} z_t$	340
		MM	$Resid(g_{10})^2 \sim \beta_0$	399
	$v_{11}(z_t, h, w)$	*BB*	$Resid(g_{11})^2 \sim \beta_0$	239
		MM	$Resid(g_{11})^2 \sim \beta_0 + \beta_1 z_t + \beta_2 w_{t+1} + \beta_3 h_{t+1} + \beta_4 w_t z_t + \beta_5 h_t z_t + \beta_6 h_{t+1} w_{t+1} + \beta_7 h_{t+1} w_{t+1} z_t$	350
Probability of reproduction (Binomial GLM)	$r(z_t, h, w)$	*BB*	$r_{t+1} \mid bolt_{t+1} = 1 \sim \beta_0 + \beta_1 z_{t+1}$	829
		MM	$r_{t+1} \mid bolt_{t+1} = 1 \sim \beta_0 + \beta_1 z_{t+1} + \beta_2 w_{t+1} + \beta_3 h_{t+1} + \beta_4 w_{t+1} z_{t+1} + \beta_5 h_{t+1} w_{t+1}$	1129
Seed set (Gaussian LM)	$f(z_t, h, w)$	*BB*	$seeds_{t+1} \mid Pr_{t+1} = 1 \sim \beta_0 + \beta_1 z_{t+1} + \beta_2 w_{t+1} + \beta_3 h_{t+1} + \beta_4 h_{t+1} z_{t+1} + \beta_5 w_{t+1} z_{t+1}$	693
		MM	$seeds_{t+1} \mid Pr_{t+1} = 1 \sim \beta_0 + \beta_1 z_{t+1} + \beta_2 w_{t+1} + \beta_3 h_{t+1} + \beta_4 h_{t+1} z_{t+1} + \beta_5 w_{t+1} z_{t+1}$	976
Dormancy/1-year seed survival* (μ and σ estimated from literature)	δ	both	0.0318	4
Germination** (Binomial distribution estimated from literature	γ	both	0.113	1220
Germinant survival** (Binomial distribution estimated from literature)	σ	both	0.645	138
Recruit size (Normal distribution)	$C(z_{t+1})$	*BB*	N(μ = 4.17 , σ = 0.88)	400
		MM	N(μ = 4.40, σ = 0.72)	400

*Mean dormancy proportion estimated separately in each of four experimental gardens across the Gunnison Valley (Anderson & Wadgymar 2020a)

**Empirical germination proportion and germinant survival for five seed sources in one experimental garden adjacent to this focal study (Anderson & Wadgymar 2020b)

Supplementary Table 6. Coefficient estimates from best-fit size- and driver-dependent vital rate regressions (VRRs) following model selection. Driver- and size-sensitive models may include effects of size (*z*), herbivory (*h*), soil moisture (*w*), and interactions, or not, depending on model selection outcomes. VRR names and functional forms are defined in Supplementary Table 5, and full outcomes of model selection are provided in Appendix S3. Cells filled in in red, blue, and gray shading represent positive, negative, and neutral effects of each driver on a given vital rate following model selection.

Genotype	VRR	Coefficient estimate from best-fit model							
		Int.	*h*	*w*	*z*	*h*×*w*	*h*×*z*	*w*×*z*	*w*×*h*×*z*
BB	*s*	-1.498	NA	20.589	0.017	NA	NA	NA	NA
MM	*s*	-1.398	-0.051	22.08	0.015	NA	0.005	-0.172	NA
BB	b_0	0.957	NA	-50.613	-2.429	NA	NA	33.062	NA
MM	b_0	-6.308	NA	NA	1.894	NA	NA	NA	NA
BB	b_1	-5.96	0.037	28.412	0.146	NA	NA	-0.535	NA
MM	b_1	-5.621	0.041	25.923	0.175	NA	NA	-0.635	NA
BB	g_{00}	1.007	-0.051	-2.088	0.383	0.466	NA	NA	NA
MM	g_{00}	0.972	-0.008	-2.208	0.425	NA	NA	NA	NA
BB	g_{01}	-14.492	-0.534	169.528	10.75	NA	NA	-35.138	NA
MM	g_{01}	7.907	0.513	NA	5.44	NA	-0.207	NA	NA
BB	g_{10}	1.085	NA	3.198	NA	NA	NA	NA	NA
MM	g_{10}	1.317	NA	NA	0.005	NA	NA	NA	NA
BB	g_{11}	4.301	1.034	78.187	0.004	-14.488	0.026	NA	NA
MM	g_{11}	16.447	-1.272	-4.625	NA	10.748	NA	NA	NA
BB	v_{00}	0.277	NA	NA	NA	NA	NA	NA	NA
MM	v_{00}	0.494	-0.007	-3.866	0.13	NA	NA	NA	NA
BB	v_{01}	-15.783	NA	900.636	NA	NA	NA	NA	NA
MM	v_{01}	90.197	NA	NA	NA	NA	NA	NA	NA
BB	v_{10}	-0.109	NA	3.695	0.023	NA	NA	-0.206	NA
MM	v_{10}	0.283	NA	NA	NA	NA	NA	NA	NA
BB	v_{11}	51.078	NA	NA	NA	NA	NA	NA	NA
MM	v_{11}	175.773	-55.586	-1173.025	-4.492	581.325	1.789	39.68	-17.851
BB	*r*	-2.425	NA	NA	0.294	NA	NA	NA	NA
MM	*r*	-4.078	-0.251	18.706	0.601	1.573	NA	-2.765	NA
BB	*f*	-45.659	-0.971	233.35	7.619	NA	0.08	-18.094	NA
MM	*f*	-39.964	-1.094	314.501	6.797	NA	0.102	-19.79	NA

References occurring in the Supporting Information

Anderson, J. T. & Wadgymar, S. M. (2020) Climate change disrupts local adaptation and favours upslope migration. *Ecology Letters* 23: 181-192.

Anderson, J. (2019a). Provenance trial experiments. figshare. Dataset. https://doi.org/10.6084/m9.figshare.10010168.v1

Anderson, J. (2019b). Integral projection models from reciprocal transplant experiments. figshare. Dataset. https://doi.org/10.6084/m9.figshare.10010141.v1

Carley, L. N., J. P. Mojica, B. Wang, *et al.* (2021) Ecological factors influence balancing selection on leaf chemical profiles in a wildflower. *Nature Ecology & Evolution* 5: 1135-1144.

Carley, L. N., Morris, W. F., Walsh, R., Riebe, D., & Mitchell-Olds, T. (2022) Are genetic variation and demographic performance linked? *Evolutionary Applications* 15: 1888-1906.

Dennis, B., Munholland, P. L., & Scott, M. J. (1991) Estimation of growth and extinction parameters for endangered species. *Ecological Monographs* 61: 115–143.

Ellner, S. P., & Rees, M. (2006) Integral projection models for species with complex demography. *The American Naturalist* 167: 410–428.

Merow, C., Dahlgren, J. P., Metcalf, C. J. E., Childs, D. Z., Evans, M. E. K., Jongejans, E., Record, S., Rees, M., Salguero-Gómez, R., & McMahon, S. M. (2014) Advancing population ecology with integral projection models: A practical guide. *Methods in Ecology and Evolution* 5: 99–110.

Visser, M. D., Schnitzer, S. A., Muller-Landau, H. C., Jongejans, E., de Kroon, H., Comita, L. S., Hubbell, S. P., & Wright, S. J. (2018) Tree species vary widely in their tolerance for liana infestation: A case study of different host response to generalist parasites. *Journal of Ecology* 106: 781–794.

Wilbur, M. Q., Langwig, K. E., Kilpatrick, A. M., McCallum, H. I., & Briggs, C. J. (2016) Integral projection models for host–parasite systems with an application to amphibian chytrid fungus. *Methods in Ecology and Evolution* 7: 1182–1194.
